# Migrant and Non-migrant Views on Immigration in Europe

**DOI:** 10.1007/s10680-025-09736-8

**Published:** 2025-06-02

**Authors:** Michaela Šedovič, Lenka Dražanová

**Affiliations:** 1 Centre for Longitudinal Studies, UCL Social Research Institute, University College London, 20 Bedford Way, London, WC1H 0AL UK; 2 Migration Policy Centre, European University Institute, Fiesole, Italy

**Keywords:** Attitudes toward immigration, Interethnic relations, Group membership, Diversity, Immigrants' integration

## Abstract

Attitudes toward immigration are usually investigated from the non-migrant residents’ perspective. Much less is known about how perceptions of immigration policy and immigrants vary across immigration background lines, especially in the wider European context, and whether migrants’ attitudes toward immigration are affected by the same factors and in the same way as those of the non-migrant population. With still-growing populations of migrants and their descendants in Europe, it is, however, crucial to study interethnic relations not only between migrant and non-migrant populations but also among different immigrant groups. Firstly, we investigate whether immigration attitudes among European migrants are based on intergroup solidarity or, rather, an intergroup threat toward new immigrants and whether minority-specific characteristics have differential effects across the (non-)migrant populations. We uncover that first- and second-generation immigrants' attitudes toward immigration are mostly guided by intergroup solidarity with other immigrants. Our results also show that first-generation immigrants' attitudes become more negative the longer they stay in the host country. Finally, we demonstrate that minority-specific characteristics work differently across our three subsamples and across different destination countries. Our analysis is based on nine rounds of the European Social Survey from 20 European countries and by estimating multilevel regression models of individual factors affecting (non-)migrants' attitudes. The findings contribute to our broader understanding of social cohesion, social inclusion and intergroup conflict.

**Supplementary Information:**

The online version contains supplementary material available at 10.1007/s10680-025-09736-8.

## Introduction

Many European societies have become more ethnically and nationally diverse in recent decades. The number of people residing in EU Member States with citizenship of a non-member country in 2021 was 23.7 million, in addition to 13.7 million persons with citizenship of another EU Member State (Eurostat, 2022). Attitudes toward immigration have, therefore, become a salient electoral issue in the last decade in Europe. However, they are usually investigated from the perspective of the majority population (Dražanová, 2022; Schneider, 2008; Semyonov et al. 2006). Nevertheless, it is crucial to study interethnic relations not only between migrant and non-migrant populations but also among different migrant groups, such as migrants already settled in and newcomers. Studying attitudes to immigration also from the immigrants' perspective can provide valuable insights into migrants’ view of a salient political issue, inform policy decisions, contribute to our deeper understanding of social inclusion, promote social cohesion and reduce intergroup conflict.

This article investigates attitudes toward immigration among first- and second-generation immigrants as well as non-migrants in 20 countries (including Eastern and Southern European ones) with three goals. Firstly, we investigate whether first- and second-generation immigrants tend to be pro- or anti-immigration, and how these attitudes compare to the non-migrant population. Compared to the plentiful studies regarding the majority’s attitudes toward immigration, there is still much less research on migrants’ immigration attitudes. Notwithstanding, those studies that, in fact, study immigrants’ attitudes toward immigration (for example, Hindriks et al., 2014; Meeusen et al., 2019) usually do so on exclusively migrant samples, without comparing them to natives. Theoretically, immigrants’ attitudes toward immigration can be attributed to intergroup solidarity or competition (Meeusen et al., 2019). For instance, immigrants and ethnic and racial minorities may be more favorable to immigration than non-migrant individuals because they can identify more strongly with other immigrants due to their own history or similar out-group status (Becker, 2019). Moreover, Hindriks et al. (2014) show that immigrants’ attitudes toward other minorities vary and that those sharing the same religion and having more contact manifested more positive attitudes toward each other.

On the other hand, when immigrants perceive a scarcity of or competition for resources in the economic or cultural sphere from other immigrant groups, they may, similarly to the majority, start to police national boundaries (Just & Anderson, 2015; Kolbe & Crepaz, 2016). Thus, we test which of the two theoretical frameworks generating two opposing predictions regarding immigrants’ attitudes toward immigration find support empirically in the European context. This includes examining whether positive or negative attitudes vary with regard to different types of immigration attitudes and different types of immigrant out-groups.

Secondly, we consider it essential to investigate whether the determinants of these attitudes may work differently for diverse segments of increasingly multicultural societies. Research explicitly looking at differences between non-migrants’ and immigrants’ attitudes (Becker, 2019; Huber & Oberdabernig, 2016; Sarrasin et al., 2018) has found that they might be shaped by distinct factors. Our findings can help policymakers design policies that better meet the needs of majority populations and immigrants, reflecting views of an important segment of the country’s society. Understanding immigrants’ attitudes can also help to create a more welcoming and inclusive society, promote social cohesion and reduce tensions between different groups. We examine whether, apart from factors usually theorized to affect the non-migrant population’s attitudes (e.g., education), minority-specific factors (e.g., belonging to a discriminated group, language) affect first and second generations’ attitudes toward immigration differently than those of non-migrants who also share these attributes. While there have already been studies regarding how stigma and perceived discrimination (Craig & Richeson, 2012; Gaertner & Dovidio, 2000; Mustafa & Richards, 2019) affect interminority attitudes, explicit comparisons of how these factors might work differently for the non-migrant versus migrant population are missing. Moreover, previous research set in Europe has mostly been conducted as single-country studies (see, for instance, Hindriks et al. (2014) for the Netherlands, Meeusen et al. (2019) for Belgium, Sarrasin et al. (2018) for Switzerland, Mustafa and Richards (2019) for the UK) or only at a handful of Western countries (e.g., Gaertner & Dovidio, 2000). It is, however, crucial to test the validity of these studies’ findings in a cross-country design as well as broaden our findings beyond the Western European focus as we do here.

Finally, the naturalization of an important share of immigrants and their descendants and the expansion of voting rights to non-nationals (Schmid et al., 2019) has led to a considerable size of a new electorate in Europe. It is paramount to understand where a non-negligible size of the electorate stands on a salient political topic. For example, Neureiter and Schulte (2024) argue that the length of stay in a host country plays a pivotal role in shaping migrants' attitudes, with longer durations often correlating with increased alignment to host population norms. We therefore also examine whether first-generation immigrants’ attitudes toward immigration change depending on the length of their tenure.

In the following sections, we elaborate on how (and if) we expect non-migrants and migrants to differ and what might theoretically explain it. We then test these theoretical propositions based on cross-national survey data spanning 16 years (2002–2018) contained in the European Social Survey (ESS) across twenty European countries (European Social Survey 2021). We show that those with a migratory background are significantly more positive toward immigration. Nevertheless, the longer migrants stay in the country, the more their attitudes converge to those of the majority and become more negative. This applies to both migrant generations. Minority-related factors affect individuals differently based on their migratory background. Finally, we also demonstrate the need to examine different attitudes toward immigration separately rather than in indices combining multiple measures. We discuss the implications of our findings, especially regarding cohesion and intergroup relations.

Our article makes several notable contributions to the study of immigration attitudes. Firstly, our findings offer significant insights into the study of immigration attitudes and their determinants, highlighting that immigrants' perspectives on immigration differ substantially from those of individuals without a migration background. Moreover, considerable variation exists within the migrant community itself, influenced by factors such as generational status and length of stay. We expand the scope of analysis to include intra-migrant variations, such as generational status, length of stay and linguistic diversity, revealing demographic complexities often overlooked in prior studies. Secondly, we add to the theoretical debates on interminority relations by elucidating the conditions under which solidarity logic prevails over threat logic and vice versa. We extend existing theoretical frameworks by integrating group empathy theory and common in-group identity models, which complement the minority solidarity and intergroup threat perspectives. Thirdly, our results demonstrate that factors such as length of residency and language dissimilarity work differently among immigrants compared to the native population.

## Theoretical Background

The theoretical framework predominantly used in explaining immigration opposition is the “competitive threat” theoretical model based on realistic conflict (Sherif, 1966) or social identity (Tajfel & Turner, 1979). According to these theories, anti-immigrant sentiment should be understood as a reaction to the threat of competition (real or perceived) with immigrants either in the economic sphere (labor, welfare) or in the cultural sphere (cultural homogeneity, social values). Within this framework, a distinction between the in-group and the (migrant) out-group is made. The in-group is mostly defined as those born in the country and/or with its citizenship, often even further restricted to individuals with parents and grandparents born in the same country. The migrant out-group can be broadly defined, as, for example, those who do not hold the country of residence’s citizenship or as those born outside of the country and/or with at least one of their parents born outside of that country.^1^ However, as Sarrasin et al. (2018) point out, it is necessary to go beyond the “us” (non-migrant) versus “them” (migrants) dichotomy when analyzing attitudes toward immigration due to the complexities of modern societies and differences between immigrants (e.g., their history, ties with destinations).

Although opposition to immigration is mostly studied from the in-group perspective, existing empirical research has shown that anti-immigration attitudes can also be prevalent among minorities, and well-established immigrants may not be exempt from being prejudiced (Hindriks et al., 2014; Mustafa & Richards 2019; Sarrasin et al., 2018). This is usually due to the perceived scarcity of resources. Based on realistic conflict theory and similarly to non-migrants, immigrants already in the country may clash with new immigrants on economic interests. They may perceive incomers as threatening their jobs if new immigrants can be hired at lower wages and unemployed individuals may perceive that the presence of such immigrants makes their job search more difficult (Gerber et al., 2017; Margalit, 2019; Scheve & Slaughter, 2001). Immigration can be regarded as additional pressure on the welfare system in terms of social benefits (Huber & Oberdabernig, 2016; Valentino et al., 2019), education (Ohinata & van Ours, 2013; Schneeweis, 2015) and public safety (Bell et al., 2013). Thus, the users of these services (non-migrant and immigrants alike) might oppose new immigration. These perceived threats may be even more prevalent among immigrants compared to non-migrants due to their higher integration and accompanying higher opportunities due to their insider status. Migrants, particularly first-generation immigrants, often occupy a more precarious socioeconomic position compared to non-migrants. This vulnerability might heighten their sensitivity to economic competition, especially from newer migrant groups. It should be, however, emphasized that these perceptions often do not reflect the real-world situation regarding immigrants’ receipt of welfare benefits or being given priority in employment before natives (Barrett & Maître, 2013; Huber & Oberdabernig, 2016). Moreover, studies have found mixed evidence for the role of realistic conflict theory in shaping non-migrants’ opinions on immigration. It appears that employment status and income have less significant effects on attitudes to immigration than measures such as perceived threats to the economy (Hainmueller & Hopkins 2014; Hendriks et al., 2022). While realistic conflict theory finds mixed support in explaining non-migrants' attitudes, it may have stronger explanatory power among migrants due to their higher dependence on overlapping economic spaces with new arrivals. This intersection of economic vulnerability and shared labor markets strengthens the salience of perceived economic competition within migrant communities.

Based on social identity theory and similarly to the members of the majority population, apart from the threat to economic interests, immigrants may perceive newcomers as a threat to the cultural character of the society. Depending on the national and cultural background of already established migrants and the level of their assimilation, new immigrants may be perceived as a threat to cultural homogeneity, value system, national and cultural identity, purity of language, etc. (Sniderman et al., 2004). Moreover, even among some of the most marginalized and stigmatized groups in Europe such as Roma migrants, Vacca et al. (2021) show that ethnic solidarity, sociodemographic homophily and network closure do not adequately explain the way migrants obtain social support. For migrants, cultural integration often represents a delicate balance between maintaining their heritage and adopting elements of the host society's culture. New arrivals, particularly those from culturally distinct backgrounds, may challenge this balance, intensifying cultural threat perceptions. Established migrants may feel that the cultural distinctiveness they have worked to preserve or integrate is undermined by newcomers who are perceived as exacerbating cultural distance from the host society (Fetzer, 2000). While non-migrants may perceive newcomers as a threat to national identity or cultural cohesion, the cultural threat posed by new groups might also be seen as a challenge to migrants’ fragile integration and acceptance within the host society. Thus, established immigrants might not be interested in supporting the arrival of new immigrants. Consequently, intergroup threat theory predicts that migrants are likely to perceive prospective immigrants as a challenge to their symbolic status and material well-being, leading to more negative attitudes compared to non-migrants.

Instead, the intergroup solidarity theoretical framework suggests that immigrants may feel solidarity (Sirin et al., 2016; Sirin et al., 2017; Eklund et al., 2009) toward newcomers due to shared experiences such as moving to another country. These shared experiences and kinship ties with other immigrants shall lead to more positive attitudes toward other immigrants (Just & Anderson, 2015). Moreover, as immigrants tend to cluster in ethnically diverse neighborhoods (Semyonov & Glikman, 2009), there might be less social distance between different immigrant groups as well as more interethnic contact and friendships (Lancee & Hartung, 2012; Lubbers et al., 2007). Potential immigration may also be viewed as enabling immigrants to form links with people who share the same culture, heritage or simply to bring their families to the country in the future (Braakmann et al., 2017).

Similarly to Meeusen et al. (2019) in the case of Belgians of Turkish and Moroccan descent, we first ask which of the two theoretical expectations is prevalent and whether immigrants’ attitudes toward immigration are shaped by intergroup threat or solidarity. Therefore, our first research question asks:

### RQ1

 Are (first- and second-generation) immigrants’ attitudes toward immigration the same or significantly different from those of the non-migrant population?

Based on the two theories emphasizing either intergroup solidarity or competition, we put forward two competing hypotheses. Based on intergroup solidarity we would expect:

### H1a

First- and second-generation immigrants hold significantly more positive attitudes toward immigration overall than the non-migrants in the same country of residence in the same time period.

Members of a specific immigrant group may worry that their cultural cohesion and status could be compromised by the growing influence of other immigrant groups. Immigrants, typically having lower socioeconomic status compared to the broader population, may also find themselves competing with prospective immigrants for manual labor jobs and welfare resources such as government housing, subsidized health care and other assistance (Collier, 2013). Therefore, immigrants are likely to be less supportive of immigration than those without a migration background, viewing prospective immigrants as a threat to their group's symbolic status and economic position.

Based on the predictions of competitive threat theory, we hypothesize:

### H1b

 First- and second-generation immigrants hold significantly more negative attitudes toward immigration overall than the non-migrants in the same country of residence in the same time period.

According to the common in-group identity model, in-group bias can be reduced when members of different groups consider themselves to belong to a shared, inclusive superordinate category (Gaertner & Dovidio, 2000). Thus, first-generation immigrants might identify themselves as belonging to the superordinate category of “immigrant,” while the second generation is likely to identify more with the population of their birth country. Previous research regarding first- and second-generation migrants' political attitudes also shows that first-generation migrants have distinct attitudes while those of non-migrant and second-generation migrant origin are similar (Maxwell, 2010; Pessin & Arpino, 2018). This is possibly due to the second generation's lower language barriers in communicating with the majority population and more intergroup contact and friendships (Martinović, 2013) or early life socialization into and formative experience with the culture of the new country (Pessin & Arpino, 2018). At the same time, we would expect the second generation to still hold more positive attitudes toward immigration compared to the country’s non-migrant population due to their personal heritage and transmission of immigration experience through their parents, albeit not as positive as the first generation’s. Based on intergroup solidarity, we would expect:

### H2a

First-generation immigrants hold more favorable attitudes toward immigration than second-generation immigrants.

Contrarily, based on competitive threat theory, we would expect:

### H2b

First-generation immigrants hold more negative attitudes toward immigration than second-generation immigrants.

Immigrants' attitudes might be explained by standard theories usually hypothesized for non-migrants as well as more minority-specific theories. There is no reason to believe that factors known to affect the non-migrant’s attitudes shall not work similarly for both migrant generations. Thus, we might expect factors such as gender, education, employment and socialization (Dražanová, 2022) to affect immigrants’ attitudes toward immigration correspondingly as they affect those of non-migrants'. Similarly, we expect non-migrants as well as both migrant generations who are citizens of the residing country to have more negative attitudes than those without citizenship. Research has shown that, across countries, immigrants who acquire citizenship become more opposed to immigration compared to those who do not (Just & Anderson, 2015; Kolbe & Crepaz, 2016; Sarrasin et al., 2018). This is thought to be due to the naturalized citizens’ increased bonds with the country’s population (Kolbe & Crepaz, 2016) or the willingness to distance themselves from “stigmatized” immigrants since immigrants often report experiences of prejudice (Jones et al., 2019; Vang & Chang, 2019; Zschirnt & Fibbi, 2019).

However, there might be specific minority-related factors playing a role in developing attitudes differently for non-migrants compared to immigrant generations. Therefore, we also ask:

### RQ2

Do factors associated with more or less stigmatization of one’s social identity (e.g., perceived discrimination, ethnic minority, speaking a non-national language) translate into intergroup solidarity or, rather, an intergroup threat toward new immigrants? Do these factors affect (non-)immigrants’ attitudes toward immigration differently?

Theoretically, there might be differential effects of perceived discrimination for different groups based on their immigration background. If public opposition to immigration is driven mostly by concerns about economic or existential threats as group conflict argues (Sherif, 1966), we would expect the most vulnerable groups (e.g., those discriminated against) to be most opposed to immigration. Contrarily, historically disadvantaged groups might find it easier to imagine themselves in the position of people being unfairly treated due to their group membership, even when those people are from a group with which they have little in common (Sirin et al., 2021). Therefore, we would expect those who feel discriminated against to be more positive toward immigrants regardless of their immigration background. We argue that there might be divergent reactions of native versus immigrant groups to immigration threats.

Based on the common in-group identity model (Gaertner & Dovidio, 2000), previous research has shown that one’s own group’s experience of discrimination might invoke a common, superordinate identity with other groups that experience prejudice and discrimination (Cortland et al. 2017). Sirin et al. (2017)’s group empathy theory showed based on a survey experiment conducted in the USA, that African-Americans and Latinos are significantly more likely to side with minority detainees and support pro-civil rights policies and actions compared to the *Anglos* majority. Craig and Richeson (2012) showed that Asian-American and Latino American participants who read about anti-Asian and anti-Latino discrimination reported more positive attitudes toward Black Americans compared to those who read a neutral article. This effect was mediated by increased feelings of similarity with Black Americans. These findings suggest that awareness of in-group disadvantage can enhance perceived similarity with other disadvantaged groups, leading to more positive attitudes toward them. We would expect empathy for immigrants to be stronger among groups with an immigration background who have experienced discrimination directly, and whose group histories resonate with the burden of living as the “other.” Accordingly, we predict that first and second generations are more likely than non-migrants to exhibit empathy for immigrants regardless of their own backgrounds.

On the other hand, since individuals derive part of their self-concept from their group memberships, when non-migrants experience discrimination, their social identity is threatened, leading to heightened sensitivity to perceived out-group threats (Tajfel & Turner, 1986). This can manifest as more negative attitudes toward immigrants, who are seen as additional out-group members potentially exacerbating their marginalization. For example, Craig et al. (2012) show that reminding White women about widespread sexism in the USA appears to have negative effects on their assessments of racial and ethnic minorities. Similarly, reading about discrimination toward their own racial in-groups caused straight Black and Latino participants to express more negative attitudes toward gay men and lesbians and reduced their support for policies benefiting sexual minorities (Craig & Richeson, 2014). Therefore, we would expect that feelings of discrimination would lead to existential and material threats and thus to more opposition toward immigration (Bai & Federico, 2020) among non-migrants.

Especially relevant for Europe is also ethnic minority membership. This membership can lead to two different theoretical expectations based on migratory background. We make a distinction between individuals with and without a migratory background in the last two generations. Those respondents that we classify to be without a migratory background (in the last two generations) and settled in Europe,^2^ being an ethnic minority should lead to more negative attitudes toward immigration due to seeing newcomers as a potential competitive threat to their already precarious position. Additionally, ethnic minorities may fear that an influx of additional ethnically distinct individuals could exacerbate their own marginalization. On the contrary, ethnic minorities with migratory backgrounds in the last two generations who went through the immigration process should hold more positive attitudes toward immigration based on solidarity with prospective immigrants and, as a result, be more supportive of immigration.

Many European countries have linguistic diversity and the language spoken at home is not necessarily national. While most people in Europe speak their national language, including nearly everyone in Poland, Greece, Hungary and France (Pew Research, 2019), the share varies substantially by country. The share of adults who speak the national language at home is smaller in, e.g., Germany, Slovakia, Spain and Bulgaria (Pew Research, 2019). These more diverse linguistic environments reflect immigration patterns, but also unique local conditions. For instance, in Germany, 2% of adults speak Turkish at home, “largely due to immigration of workers from Turkey in the 1960s” and Turkish is spoken by 14% of adults in Bulgaria (Pew Research, 2019). Similarly, Romani, a language spoken by Europe’s Roma people, appears as a language primarily used at home in Bulgaria (6%), the Czech Republic (2%), Hungary (2%) and Slovakia (1%) (Pew Research, 2019). Therefore, speaking non-national languages at home is not strictly connected to immigration.

Non-migrants who speak foreign languages at home may already feel a sense of marginalization or lack of full belonging within the broader national identity. This heightened awareness of their distinctiveness can lead to stronger in-group identification and more pronounced out-group derogation. As a result, these individuals might attempt to uplift their group esteem by devaluing other vulnerable groups, such as prospective immigrants (Brylka et al., 2016). Finally, non-migrants speaking foreign languages at home might face societal pressures to assimilate and adopt the dominant culture. The presence of new immigrants who maintain their cultural and linguistic distinctiveness can intensify these pressures, leading to a backlash against immigration. Their resistance to immigration may be driven by a desire to preserve their own cultural identity while feeling threatened by the increasing cultural diversity brought by new immigrants. For this reason, we expect differential effects, again, for non-migrants and immigrants. While we hypothesize that speaking foreign languages at home would lead to more negative attitudes among the non-migrant population, we expect the opposite to happen with immigrants.^3^

These relationships capture the differential immigrants’ position compared to non-migrants regarding objective and subjective group positions. Therefore, we hypothesize:

### H3a

Due to intergroup solidarity, perception of discrimination, being an ethnic minority and/or speaking a non-national language leads to more positive immigration attitudes among first- and second-generation immigrants.

In summary, non-migrants who feel discriminated against, are an ethnic minority and/or speak a foreign language at home may develop more negative attitudes toward immigration due to perceived threats to their social identity, competition over resources, feelings of relative deprivation and pressures to assimilate into the dominant culture. Salient in-group discrimination or identity different from the majority seems to have a negative effect on evaluations of out-groups that are disadvantaged on a dimension that differs from the in-group (Cortland et al. 2017).

Thus, we hypothesize:

### H3b

Due to competitive threat, perception of discrimination, being part of an ethnic minority and/or speaking a non-national language leads to more negative immigration attitudes among non-migrants.

Finally, there might be differences not only between the migrant generations but also within the first generation. Earlier and recent immigrants may hold different views on further immigration. Similarly, to the theoretical arguments regarding the second generation, longer-staying first-generation immigrants may have already integrated into the host society to a larger extent (Manning & Roy, 2010) in the form of language acquisition and intergroup friendships, and their attitudes toward immigration might start converging to (more or less favorable) attitudes held by the population in their new country of residence. Neureiter and Schulte (2024) highlighted that the length of stay in a host country influences migrants' attitudes toward immigration, often aligning with majority norms over time. Our study extends this framework by examining how these dynamics play out in Eastern and Southern European contexts, where immigration histories and integration policies differ significantly. We also explore whether this dynamic differs across types of attitudes, such as openness to immigration versus perceptions of immigration’s cultural impact.

Thus, we ask:

### RQ3

Do first-generation immigrants’ attitudes toward immigration converge with those of the non-migrant population of their country of residence after they live long enough in their host country?

To answer this question, we restricted our sample to the first-generation immigrants. We compared those with a longer tenure in the destination to those arriving recently and expect the first generation’ attitudes toward immigration to be more comparable to those of non-migrants' with time:

### H4

The more years the first-generation immigrants stay in a destination, the more their attitudes toward immigration differ (whether they are more or less favorable) from those of the first-generation immigrants arriving recently.

Overall immigration attitudes entail several attitudes toward immigrants measures considering different groups and attitudes toward various effects of immigration on the country. Although these generally correlate, some individuals may, in fact, hold diverse, positive or negative, opinions depending on the specific immigration attitude in question. For instance, an individual might be against allowing more immigrants into the country, while simultaneously advocating for (welfare or other) rights of the immigrants already in the country. Moreover, a recent meta-analysis of factors affecting attitudes toward immigration (Dražanová et al., 2022) shows that factors influencing these attitudes differ based on the immigration attitudes in question. Moreover, we might expect that particular attitudes might converge at different speeds depending on various factors affecting them. Thus, we hypothesize that there is a variation in how particular attitudes toward immigration develop across first-generation immigrants and that different attitudinal responses among them may occur.

## Data and Methods

### Data

This research uses data from the European Social Survey (ESS) collected between 2002 and 2018 in 20 European countries. The ESS is a cross-sectional survey conducted biennially since 2002 and includes most European countries. We employ all currently fully available rounds of data collection. Our data includes Austria, Belgium, Croatia, Czechia, Denmark, Estonia, Finland, France, Germany, Greece, Ireland, Latvia, Lithuania, the Netherlands, Norway, Portugal, Slovenia, Sweden, Switzerland and the UK. The country sample is restricted by the minimal number of observations per country and round for each generation (*N* > 29, see Tables A1-A5 in Online Appendix).

While studying the attitudes of the migrant population is not the primary purpose of the ESS, it is widely used for investigating them Lo (Safi, 2010; Kogan et al., 2018; Gonnot & Lo Polito, 2021). The dataset allows us to compare the attitudes toward immigrants among non-migrant and migrant populations while recognizing different generations and groups. The sample we use (*N* = 265,698) comprises three subpopulations. First, it is the non-migrant population (*N* = 222,336), which we define as individuals born in their country of residency (and of the interview) with both of their parents born there. Second, it is first-generation immigrants, consisting of all individuals born outside of the country of the interview with both parents also born outside (*N* = 22,273). Lastly, it is the second generation, including individuals born in the country of the interview but to one parent who was not born there (*N* = 21,089).^4^ We are aware these measures and definitions of non-migrants and migrant generations are not without flaws. There certainly can be individuals who are born outside of the country but could be considered non-migrants. However, apart from these nuances, we follow the standard methodological distinction made by most researchers studying migrant populations (Hooghe & Quintelier, 2013; Johnston et al., 2015; Safi, 2010).

### Key Response Variables

Our response variables are 6 indicators of attitudes toward immigration, which are included in the ESS questionnaire in each wave. Three measures cover attitudes toward immigration policy and open borders and distinguish between different types of immigrant groups. The other three ask about respondents’ opinions regarding immigration’s effect on the country, culture and economy. Table A6 in Online Appendix reports the question wordings and scales. Three of the indicators are measured on a 4-point Likert scale, while the rest is measured on an 11-point scale. To ease the comparison between the two sets of indicators, we rescale the 11-point scales to range from 1 to 4. We ran a series of multilevel hierarchical models for each of them separately. This allows us to compare how different (non-)migrant generations vary in their attitudes toward different aspects of immigration as well as to see what determines it.

### Explanatory and Control Variables

Our main explanatory variables are indicators of the migratory background of an individual (two binary variables—one for first-generation immigrants and one for second-generation immigrants—while respondents with a non-immigration background are the reference category). However, immigrants are not a homogeneous group, for example in terms of their time since arrival, educational attainment, employment, etc. To capture this heterogeneity and to disentangle significant differences in the determinants of different immigration attitudes, we include several independent variables.

As established in the theory section, we employ determinants previously associated with non-migrants' attitudes toward immigration (Ceobanu & Escandell, 2010; Dražanová, 2022; Hainmueller & Hopkins, 2014) and immigrants' attitudes (Gonnot & Lo Polito 2021; Rustenbach, 2010; Meuleman et al., 2009; Becker, 2019). We control for gender, age, employment status, educational attainment, self-assessed income, frequency of socialization, religious denomination, religiosity and citizenship. We also include measures indicating experienced discrimination,^5^ belonging to an ethnic minority, and the first and second language spoken at home^6^ as we are interested in the potentially different associations with the immigrant and non-migrant groups.^7^ However, controlling for variables such as employment status, income or socialization frequency can introduce bias if these variables are affected by the independent variable of interest (e.g., migratory background). For example, migrants may have lower employment rates or incomes due to structural barriers in the labor market, and these characteristics could influence their attitudes toward immigration. This may lead to an underestimation of the total effect of migratory background on immigration attitudes. In models assessing the effect of lengths of stay on first-generation immigrants we also include the first generation’s time since arrival (5 categories) and their region of origin.^8^ Tables A7 and A8 in Online Appendix report all descriptive statistics for the independent variables. Since we employ a multilevel hierarchical regression model, which controls for both time and country effects, we forego the inclusion of specific national-level controls. There are 9,7% of cases with missing data overall, which we deleted listwise.

### Method

Our main objectives in this research are: (1) to empirically analyze the differences in attitudes toward immigration among non-migrants and immigrant generations, (2) to analyze and compare the individual-level determinants influencing these attitudes, especially factors associated with more or less stigmatization of one’s social identity, and (3) to investigate whether immigrants’ attitudes toward immigration converge toward those of the non-migrant population as they stay longer in the host country. Thus, our first goal is to summarize attitude patterns among the three samples. We are particularly interested in the variation depending on the migrant generation and the differences between migrant generations and non-migrants. Our second focus is on the variation in the association between specific explanatory variables and attitudes toward immigration for the three samples. Thirdly, we analyze, within the first-generation sample, how lengths of stay may affect immigrants' attitudes toward immigration. Considering the hierarchical structure of our data indicated by its multinational character and several data collection time points, when countries are nested in country-years, we employ a three-level multilevel hierarchical regression model. This model groups individuals into country-years, which are nested within countries. The model recognizes that respondents from the same country are more similar than respondents from different countries while also recognizing that respondents observed in the same country in the same year have more in common than respondents observed in the same country across years (Schmidt-Catran & Fairbrother, 2016). As we are not interested in the effects of any year-level variables, a three-level hierarchical model is the most parsimonious (Schmidt-Catran & Fairbrother, 2016). In models with the full sample, we also include a random slope for each generation (first- and second-generation immigrants) to allow the intercepts to vary across generations, as we do not expect the same effects of migrant generations on attitudes across countries. We use a three-level hierarchical model where respondents (level 1) are nested within country-years (level 2), which are further nested within countries (level 3). To account for broader temporal dynamics affecting all countries, we include year fixed effects in the model. This combination allows us to simultaneously model clustering effects at the country-year and country levels while controlling for global time trends across survey years.

However, we also test the fit of less complex fixed effects models, and multilevel hierarchical regression models are a better fit (see Tables A9b and A10b in Appendix).

## Results

We begin our empirical analysis using multilevel modeling by estimating the null model for each dependent variable. The null model provides an assessment of whether significant between-group variation exists and thus whether a multilevel analysis is necessary. It also serves as a useful baseline model for subsequently evaluating explained variance. This model is referred to as “null” because it has no predictors at any level and includes only an intercept, country-year effects and country effects. It, therefore, provides a predicted value for the mean, which is not conditional on any covariates.

Tables 1 and 2 report the null models (Model 0) for each dependent variable.^9^ The random effects reported for each model show the variance components in multilevel models, which can be interpreted with the variance partition coefficients (VPCs). VPCs report the proportion of the observed response variation that lies at each level of the model hierarchy. They, therefore, allow us to establish the relative importance of countries, country-periods and individual respondents as sources of variation in respondents’ mean attitudes toward immigration. The respective percentages of variation at each level are shown in Table 3. Most of the variation in attitudes lies within countries at the individual level (about 90% of the overall variation for each dependent variable), around 3–11% of the variation lies between countries and around 1–3% lies within country-periods. Thus, there are relatively small differences in individual attitudes between countries and between time periods. In summary, the VPCs show that there is a relatively low degree of clustering in the data.^10^

**Table 1:** Results for a three-level multilevel hierarchical model for attitudes regarding open borders in the full sample

	Allow different race						Allow same race						Allow poor countries					
	Model 0		Model 1		Model 2		Model 0		Model 1		Model 2		Model 0		Model 1		Model 2	
	Coef	S.E	Coef	S.E	Coef	S.E	Coef	S.E	Coef	S.E	Coef	S.E	Coef	S.E	Coef	S.E	Coef	S.E
Intercept	2.531***	(0.059)	2.643***	(0.060)	2.625***	(0.061)	2.827***	(0.050)	2.727***	(0.051)	2.714***	(0.052)	2.440***	(0.062)	2.711***	(0.064)	2.695***	(0.066)
*Individual level*																		
First generation			0.127***	**(0.008)**	0.162***	**(0.034)**			0.123***	**(0.007)**	0.151***	**(0.029)**			0.095***	**(0.007)**	0.126***	**(0.031)**
Second generation			0.098***	**(0.006)**	0.108***	**(0.013)**			0.094***	**(0.005)**	0.098***	**(0.011)**			0.093***	**(0.006)**	0.099***	**(0.012)**
Age			− 0.008***	(0.001)	− 0.007***	(0.0005)			− 0.006***	(0.001)	− 0.005***	(0.004)			− 0.009***	(0.005)	− 0.009***	(0.001)
Male			− 0.018***	(0.003)	− 0.018***	(0.003)			0.002	(0.003)	0.001	(0.003)			− 0.036***	(0.003)	− 0.036***	(0.003)
Upper Secondary			0.099***	(0.004)	0.098***	(0.004)			0.106***	(0.004)	0.104***	(0.004)			0.066***	(0.004)	0.064***	(0.004)
Non-university tertiary			0.179***	(0.008)	0.180***	(0.008)			0.175***	(0.007)	0.175***	(0.007)			0.133***	(0.008)	0.132***	(0.008)
University degree			0.377***	(0.004)	0.377***	(0.004)			0.347***	(0.004)	0.346***	(0.004)			0.320***	(0.004)	0.319***	(0.004)
Employed			− 0.015***	(0.003)	− 0.016***	(0.004)			− 0.026***	(0.003)	− 0.027***	(0.003)			− 0.014***	(0.004)	− 0.015***	(0.004)
Coping on income			− 0.092***	(0.004)	− 0.090***	(0.003)			− 0.098***	(0.003)	− 0.096***	(0.003)			− 0.080***	(0.003)	− 0.079***	(0.003)
Difficult on income			− 0.186***	(0.005)	− 0.184***	(0.005)			− 0.189***	(0.005)	− 0.187***	(0.005)			− 0.163***	(0.005)	− 0.161***	(0.005)
Very difficult on income			− 0.278***	(0.008)	− 0.278***	(0.008)			− 0.294***	(0.007)	− 0.294***	(0.007)			− 0.244***	(0.008)	− 0.244***	(0.008)
Socializing Monthly			0.125***	(0.013)	0.125***	(0.013)			0.175***	(0.013)	0.175***	(0.013)			0.128***	(0.014)	0.127***	(0.014)
Socializing Weekly			0.185***	(0.013)	0.185***	(0.013)			0.245***	(0.013)	0.244***	(0.013)			0.179***	(0.014)	0.179***	(0.014)
Socializing Daily			0.238***	(0.013)	0.238***	(0.013)			0.290***	(0.013)	0.289***	(0.013)			0.230***	(0.014)	0.229***	(0.014)
Discriminated group			0.033***	(0.009)	0.027**	(0.009)			0.037***	(0.009)	0.030**	(0.009)			0.045***	(0.010)	0.039***	(0.010)
Ethnic minority			0.066***	(0.008)	0.077***	(0.008)			0.033***	(0.008)	0.041***	(0.008)			0.058***	(0.008)	0.062***	(0.008)
Citizenship			− 0.008***	(0.001)	− 0.007***	(0.001)			− 0.007***	(0.001)	− 0.006***	(0.001)			− 0.008***	(0.001)	− 0.007***	(0.001)
Minority language			− 0.023*	(0.009)	− 0.014	(0.009)			− 0.004	(0.008)	0.002	(0.009)			− 0.034***	(0.009)	− 0.034***	(0.009)
Religiosity			0.006***	(0.001)	0.006***	(0.001)			0.006***	(0.001)	0.007***	(0.001)			0.010***	(0.001)	0.010***	(0.001)
*Random effects estimates*																		
Country	**0.067**	**(0.022)**	**0.0436**	**(0.014)**	**0.047**	**(0.016)**	**0.047**	**(0.016)**	**0.027**	**(0.009)**	**0.029**	**(0.009)**	**0.074**	**(0.024)**	**0.054**	**(0.017)**	**0.058**	**(0.019)**
Period (in country)	**0.020**	**(0.002)**	**0.0146**	**(0.001)**	**0.014**	**(0.002)**	**0.020**	**(0.002)**	**0.012**	**(0.001)**	**0.013**	**(0.002)**	**0.020**	**(0.002)**	**0.014**	**(0.001)**	**0.014**	**(0.001)**
Individual	**0.667**	**(0.002)**	**0.6106**	**(0.001)**	**0.609**	**(0.002)**	**0.625**	**(0.002)**	**0.586**	**(0.001)**	**0.585**	**(0.002)**	**0.698**	**(0.001)**	**0.647**	**(0.001)**	**0.646**	**(0.001)**
First generation					**0.022**	**(0.007)**					**0.016**	**(0.005)**					**0.017**	**(0.005)**
Second generation					**0.002**	**(0.001)**					**0.002**	**(0.001)**					**0.002**	**(0.001)**

The full model includes religious denomination and year/survey fixed effect as a control variable, which is not shown here. Please refer to Appendix Table A9a. Please refer to Appendix Table A10c to see Model 0 with the first and the second generation only

alpha(0.001, 0.01, 0.05) symbol(***, **, *)

**Table 2:** Results for a three-level multilevel hierarchical model for attitudes regarding the effects of immigration in the full sample

	Immigration's effect on country						Immigration’s effect on economy						Immigration’s effect on culture					
	Model 0		Model 1		Model 2		Model 0		Model 1		Model 2		Model 0		Model 1		Model 2	
	Coeff	S.E	Coeff	S.E	Coeff	S.E	Coeff	S.E	Coeff	S.E	Coeff	S.E	Coeff	S.E	Coeff	S.E	Coeff	S.E
Intercept	2.467***	**(0.044)**	2.465***	**(0.042)**	2.452***	**(0.043)**	2.487***	**(0.037)**	2.411***	**(0.038)**	2.390***	**(0.040)**	2.647***	**(0.055)**	2.631***	**(0.052)**	2.615***	**(0.055)**
*Individual level*																		
First generation			0.183***	(0.006)	0.192***	(0.027)			0.169***	(0.006)	0.182***	(0.034)			0.158***	(0.006)	0.176***	(0.034)
Second generation			0.076***	(0.004)	0.073***	(0.010)			0.072***	(0.005)	0.075***	(0.013)			0.099***	(0.005)	0.099***	(0.012)
Age			− 0.004***	(0.001)	− 0.004***	(0.001)			− 0.003***	(0.001)	− 0.003***	(0.001)			− 0.0007	(0.001)	− 0.0006	(0.001)
Male			− 0.004	(0.002)	− 0.004	(0.002)			0.066***	(0.002)	0.066***	(0.002)			− 0.039***	(0.002)	− 0.039***	(0.002)
Upper Secondary			0.071***	(0.003)	0.069***	(0.003)			0.091***	(0.003)	0.089***	(0.003)			0.099***	(0.003)	0.098***	(0.003)
Non-university tertiary			0.138***	(0.006)	0.139***	(0.006)			0.156***	(0.006)	0.157***	(0.006)			0.175***	(0.006)	0.176***	(0.006)
University degree			0.280***	(0.004)	0.279***	(0.004)			0.350***	(0.004)	0.348***	(0.004)			0.361***	(0.004)	0.360***	(0.003)
Employed			− 0.003	(0.003)	− 0.003	(0.003)			− 0.009**	(0.003)	− 0.010**	(0.003)			− 0.003	(0.003)	− 0.004	(0.003)
Coping on income			− 0.089***	(0.003)	− 0.088***	(0.003)			-0.106***	(0.003)	− 0.104***	(0.003)			− 0.089***	(0.003)	− 0.088***	(0.003)
Difficult on income			− 0.180***	(0.004)	− 0.178***	(0.004)			− 0.204***	(0.004)	− 0.202***	(0.004)			− 0.162***	(0.004)	− 0.161***	(0.004)
Very difficult on income			− 0.274***	(0.006)	− 0.273***	(0.006)			− 0.297***	(0.006)	− 0.296***	(0.006)			− 0.231***	(0.006)	− 0.231***	(0.006)
Socializing Monthly			0.103***	(0.011)	0.102***	(0.011)			0.123***	(0.012)	0.122***	(0.012)			0.120***	(0.012)	0.119***	(0.012)
Socializing Weekly			0.158***	(0.010)	0.157***	(0.011)			0.193***	(0.011)	0.192***	(0.012)			0.180***	(0.012)	0.178***	(0.012)
Socializing Daily			0.181***	(0.011)	0.180***	(0.011)			0.208***	(0.011)	0.208***	(0.012)			0.215***	(0.012)	0.214***	(0.012)
Discriminated group			− 0.044***	(0.007)	− 0.049***	(0.007)			− 0.006	(0.008)	− 0.016	(0.008)			− 0.024**	(0.008)	− 0.033***	(0.008)
Ethnic minority			0.075***	(0.006)	0.082***	(0.006)			0.063***	(0.007)	0.075***	(0.007)			0.083***	(0.007)	0.089***	(0.007)
Citizenship			− 0.011***	(0.001)	− 0.010***	(0.001)			− 0.012***	(0.001)	− 0.011***	(0.001)			− 0.009***	(0.001)	− 0.008***	(0.001)
Minority language			0.003	(0.007)	0.014	(0.007)			− 0.007	(0.007)	0.010	(0.008)			− 0.068***	(0.007)	− 0.059***	(0.008)
Religiosity			0.012***	(0.001)	0.012***	(0.001)			0.009***	(0.001)	0.009***	(0.001)			0.008***	(0.001)	0.008***	(0.001)
*Random effects estimates*																		
Country	**0.037**	**(0.012)**	**0.022**	**(0.007)**	**0.025**	**(0.008)**	**0.026**	**(0.009)**	**0.014**	**(0.005)**	**0.016**	**(0.005)**	**0.061**	**(0.019)**	**0.042**	**(0.013)**	**0.047**	**(0.015)**
Period (in country)	**0.008**	**(0.001)**	**0.005**	**(0.001)**	**0.005**	**(0.001)**	**0.012**	**(0.002)**	**0.006**	**(0.001)**	**0.007**	**(0.001)**	**0.006**	**(0.001)**	**0.005**	**(0.001)**	**0.005**	**(0.001)**
Individual	**0.418**	**(0.001)**	**0.385**	**(0.001)**	**0.384**	**(0.001)**	**0.478**	**(0.001)**	**0.439**	**(0.001)**	**0.438**	**(0.001)**	**0.498**	**(0.001)**	**0.456**	**(0.013)**	**0.455**	**(0.001)**
First generation					**0.013**	**(0.004)**					**0.023**	**(0.007)**					**0.022**	**(0.007)**
Second generation					**0.001**	**(0.001)**					**0.003**	**(0.001)**					**0.002**	**(0.001)**
Observations	**257,906**		**257,801**		**257,801**		**257,277**		**257,174**		**257,174**		**258,330**		**258,229**		**258,229**	

The full model includes religious denomination and year/survey fixed effect as a control variable, which is not shown here. Please refer to Appendix Table A10a. Please refer to Appendix Table A10c to see Model 0 with the first and the second generation only

alpha(0.001, 0.01, 0.05) symbol(***, **, *)

**Table 3:** Variance partition coefficients

Model	Allow different race			Allow same race			Allow from poorer countries			Immigration effect on country			Immigration effect on economy			Immigration effect on culture		
	0	1	2	0	1	2	0	1	2	0	1	2	0	1	2	0	1	2
Country (%)	8.9	6.5	7	6.8	4.3	4.6	9.3	7.5	8	8	5.3	6	5	3	3.5	10.8	8.3	9.3
Period (%)	2.7	2.2	2	2.9	2	2	2.6	2	2	1.7	1.3	1.2	2.3	1.3	1.5	1.1	1	1
Individual (%)	**88.4**	**91.3**	**91**	**90.3**	**93.7**	**93.4**	**88.1**	**90.5**	**90**	**90.3**	**93.4**	**92.8**	**92.7**	**95.7**	**95**	**88.1**	**90.7**	**89.7**

In the next stage, we estimate models with individual-level predictors and the multilevel specification. Models 1 in Tables 1 and 2 show the results for each dependent variable. We are mainly interested in the independent variables referring to the respondents’ migration status (first and second generation). In the case of all dependent variables, first- and second-generation respondents are significantly more pro-immigration compared to non-migrant respondents. Those belonging to the first generation hold more pro-immigration attitudes compared to the second generation, as documented by the higher estimated effects of the respective variables. This confirms our first hypotheses H1a and H2a. Therefore, first- and second-generation immigrants’ attitudes toward immigration seem to be driven by intergroup solidarity rather than threat (refuting our alternative hypotheses). Nevertheless, it is worth highlighting that differences between immigrant generations are more pronounced for attitudes regarding immigration’s effect on the country. Differences between generations regarding allowing immigrants into the country are smaller, and the differences between the two generations in allowing people from poorer countries to come are negligible.

While the differences between migrant and non-migrant groups may appear modest in size, they are substantively significant given the demographic weight of these populations in Europe. The observed patterns align with theoretical expectations of intergroup solidarity and competitive threat and reveal important nuances in attitudes across groups and contexts.

Models 1 in Tables 1 and 2 enable attitudes to depend on individuals’ characteristics, their country of residence as well as the period (within the country) when respondents were surveyed. However, this type of model assumes that the effects of individuals’ characteristics such as their migratory background on attitudes toward immigration are the same in each country. That is to say, the coefficients of all explanatory variables are fixed across countries. In the next set of models (Models 2 in Tables 1 and 2), we allow both the intercept and the coefficients for first and second generation to vary randomly, thus employing a random coefficient model. Our choice to let the coefficients for the two independent variables vary at the country level rather than country-period level is driven by theoretical and empirical reasons. Firstly, while it is theoretically reasonable to expect that the effect of migratory background might affect attitudes differently in each country, it is less so to expect that these effects would significantly vary from year to year within one country. Secondly, this is empirically confirmed by the minimal unexplained variance in individual attitudes toward immigration at the country-period level. Since our dependent variable does not vary a lot at this level, it is reasonable to expect that also relationships between independent variables and the dependent one would vary more across countries. Likelihood ratio tests confirmed that the first and second generations’ effects differ across countries.

As previously mentioned, the model assumes that the effects of all variables in the model, except first and second generation, are fixed across countries and their estimated effects are very similar for both, the random intercept and random coefficient model. The first-generation and second-generation coefficients have a fixed component, representing contrasts with the reference category (non-migrants) on average, and a country-specific component. For example, after accounting for the effects of control variables, first-generation immigrants in country *c* are expected to have their willingness to allow immigrants of a different race 0.184^11^ points higher than non-migrants in the same country, while second-generation migrants are expected to have their willingness 0.11^12^ points higher compared to non-migrants.

**Fig. 1:**
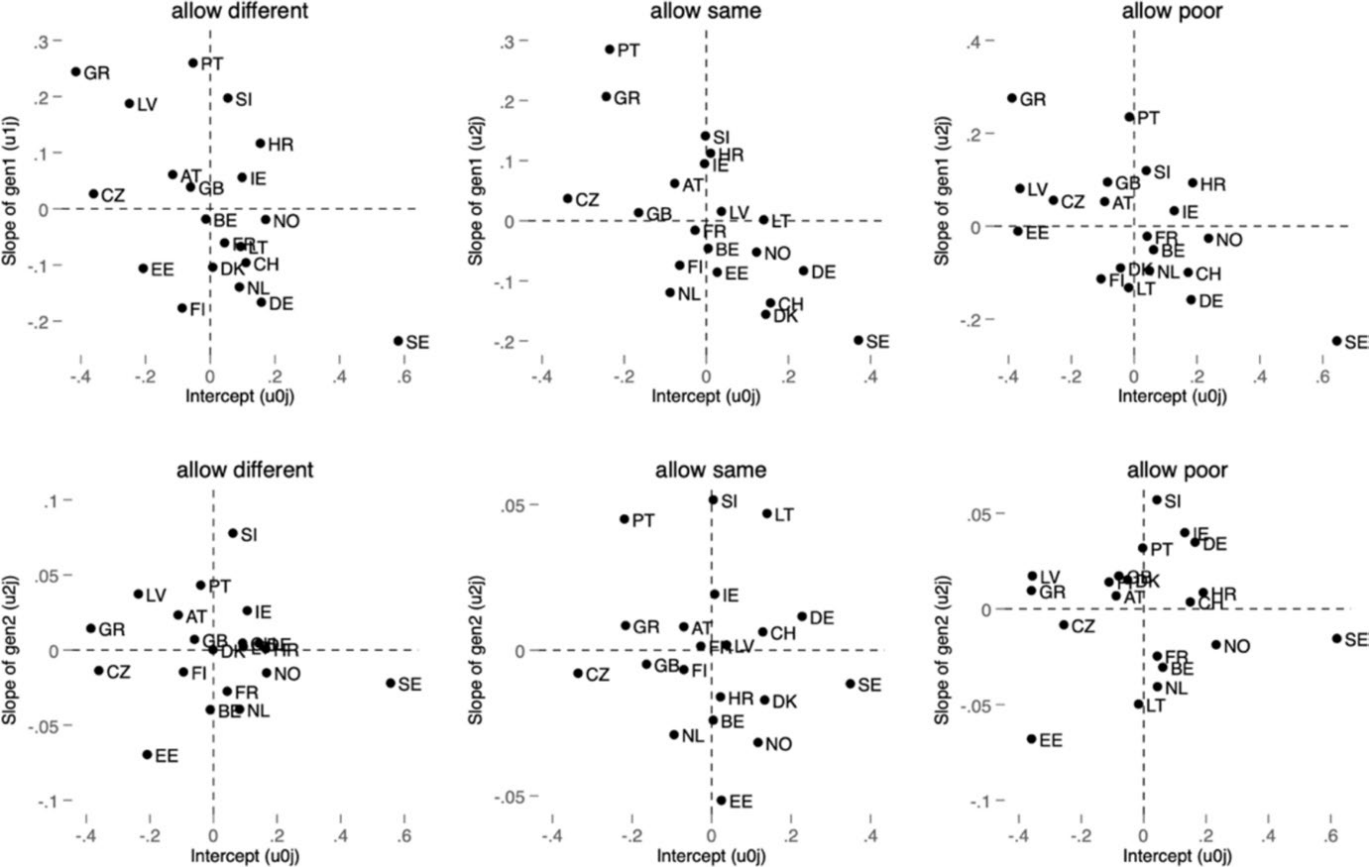
Plot of random slopes and random intercepts for first- and second-generation immigrant across countries on attitudes toward immigration policy

**Fig. 2:**
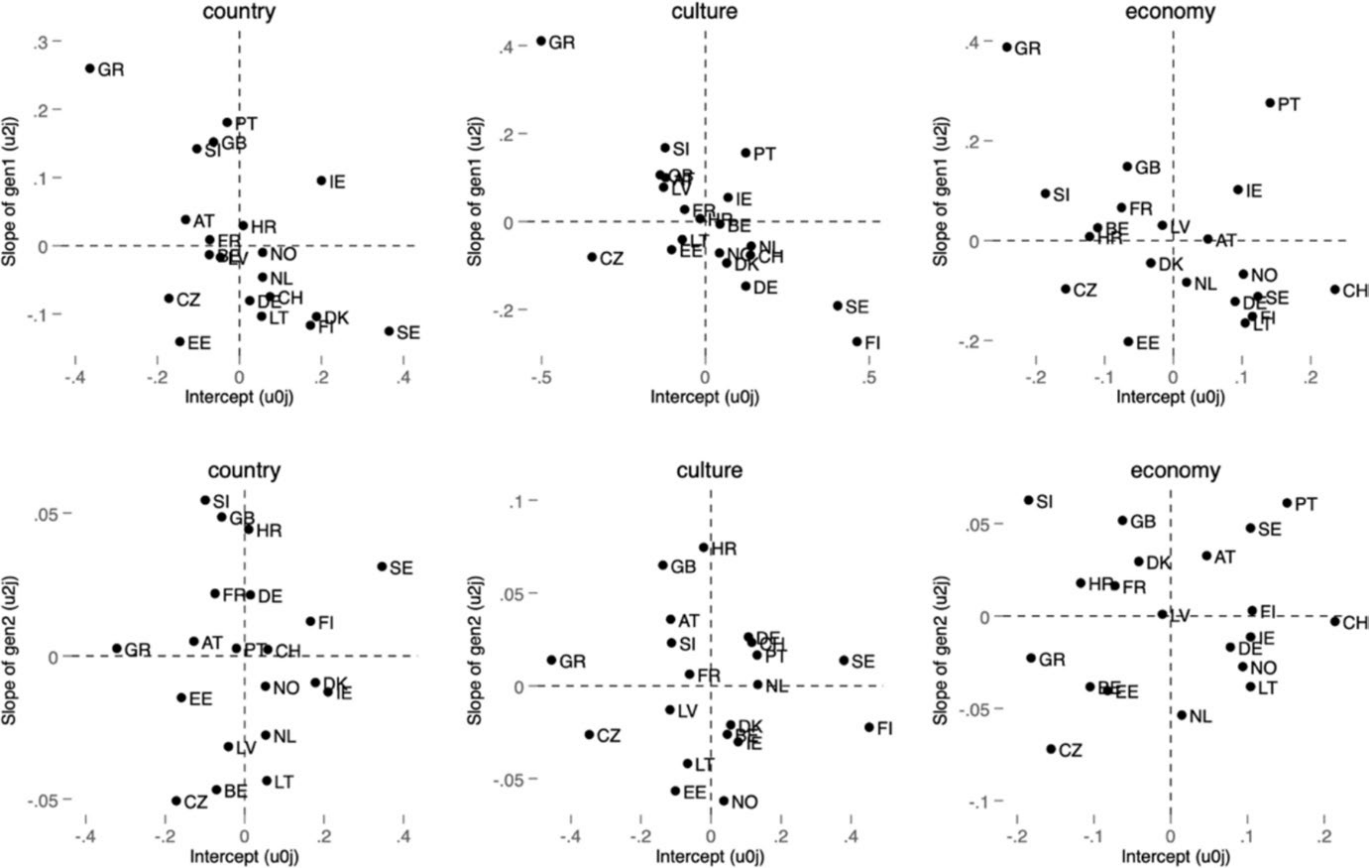
Plot of random slopes and random intercepts for first- and second-generation immigrant across countries on attitudes toward the effect of immigration

Figures 1 and 2 show the plot of the generation slope residuals versus the country intercept residuals for all dependent variables from Models 2 in Tables 1 and 2. From these plots, it is possible to identify, e.g., countries with lower-than-average positive attitudes (all those on the left side from the 0 on the x-axis) or higher-than-average positive attitudes toward immigration (all those on the right of the x-axis). These are, for example, Sweden in all types of attitudes and Germany and Switzerland (especially for immigrants’ economic contribution). Moreover, the plots show countries with the strongest relationship between having an immigration background and positive attitudes (the top quadrants). Therefore, countries in the top right quadrant are countries with the highest proportion of positive attitudes and at the same time the strongest relationship between a migratory background and attitudes toward immigration. On the other hand, countries in the top left quadrant have a below average positive attitude to immigration, but the effect of an immigrant background is stronger than average.

These figures highlight the importance of distinguishing between different attitudes toward immigration. While Sweden, in line with previous research, appears to be above average in all measures compared to other countries, the effect of immigration background is weak regarding attitudes toward immigration policy but much stronger in the evaluation of immigration’s effect. Notwithstanding, the above average effect compared to other countries with regard to immigration’s contribution is pronounced only with respect to the second generation. In contrast, the largest negative intercept and slope for the first-generation immigrants belong to Greece. These figures emphasize that countries may differ not only in the effect of immigration background on attitudes but also in the strengths of the effects of different generations within one country. While it is not an aim of this paper to describe each studied country, we recognize that the relationship between independent and dependent variable differs across countries, especially among regions with a longer and shorter history of immigration (e.g., Western vs. Easter Europe). This has implications for the interpretation of our results, but also beyond this paper. The diversity within Europe in this issue needs to be recognized in all comparative analyses including all or most of the European countries. For example, Figs. 1 and 2 show that while the 2004 enlargement countries are below the European average on most immigration attitudes, the positive effects of first-generation immigrants are especially pronounced in some of these countries (in Czechia, Slovenia, Latvia) with regard to immigration policy. In contrast, the effect of first generation on attitudes toward immigrant contribution is below the average. Similarly, the effect of the second generation is below average for most of the countries in Central and Eastern Europe. Figures 1 and 2 illustrate that while the average differences between groups (e.g., first-generation migrants, second-generation migrants and non-migrants) may appear modest in magnitude, these effects provide valuable insights into the contextual variability of attitudes, highlighting the role of diverse national contexts in shaping attitudes toward immigration.

**Fig. 3:**
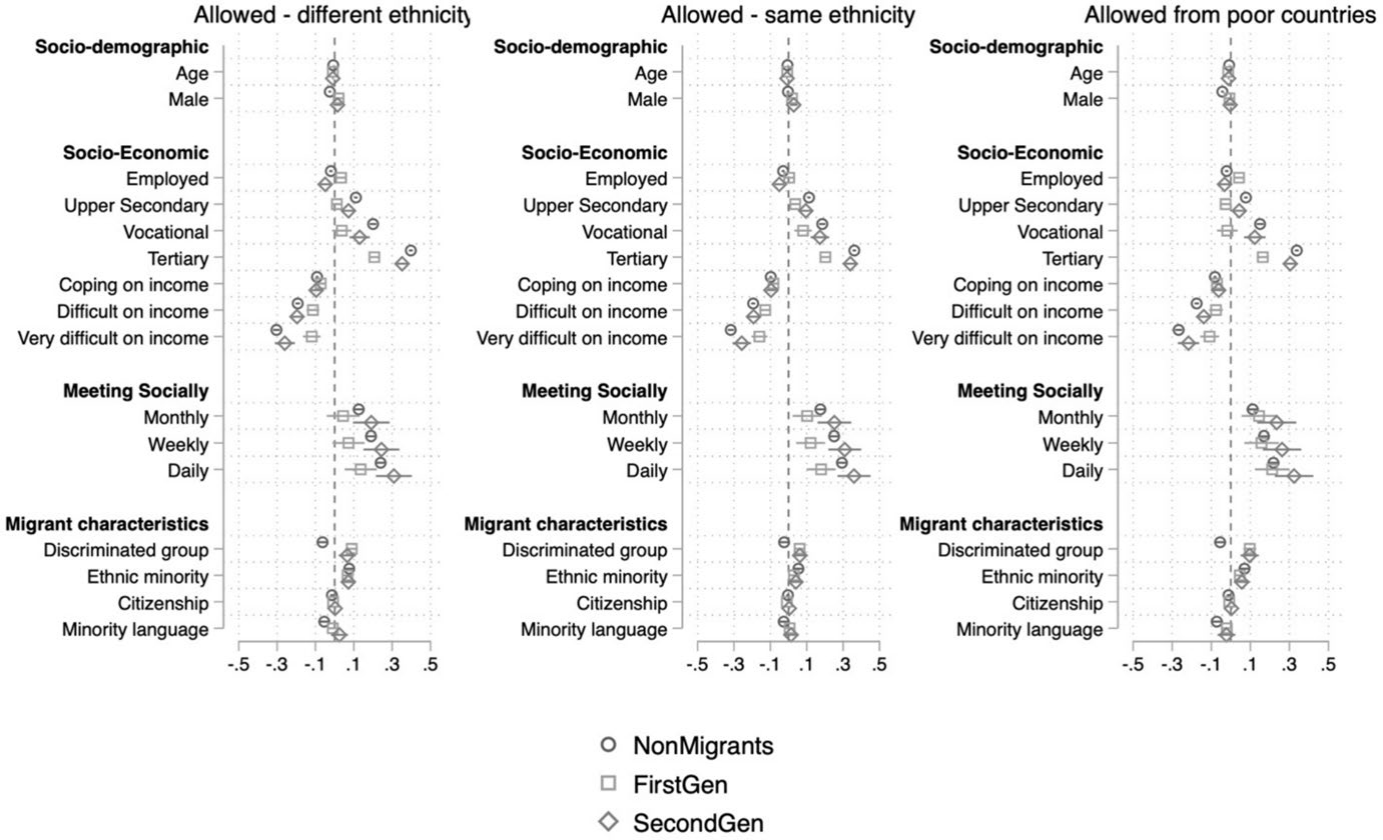
Determinants of attitudes toward immigration policy among non-migrants, first-generation immigrants and second-generation immigrants

**Fig. 4:**
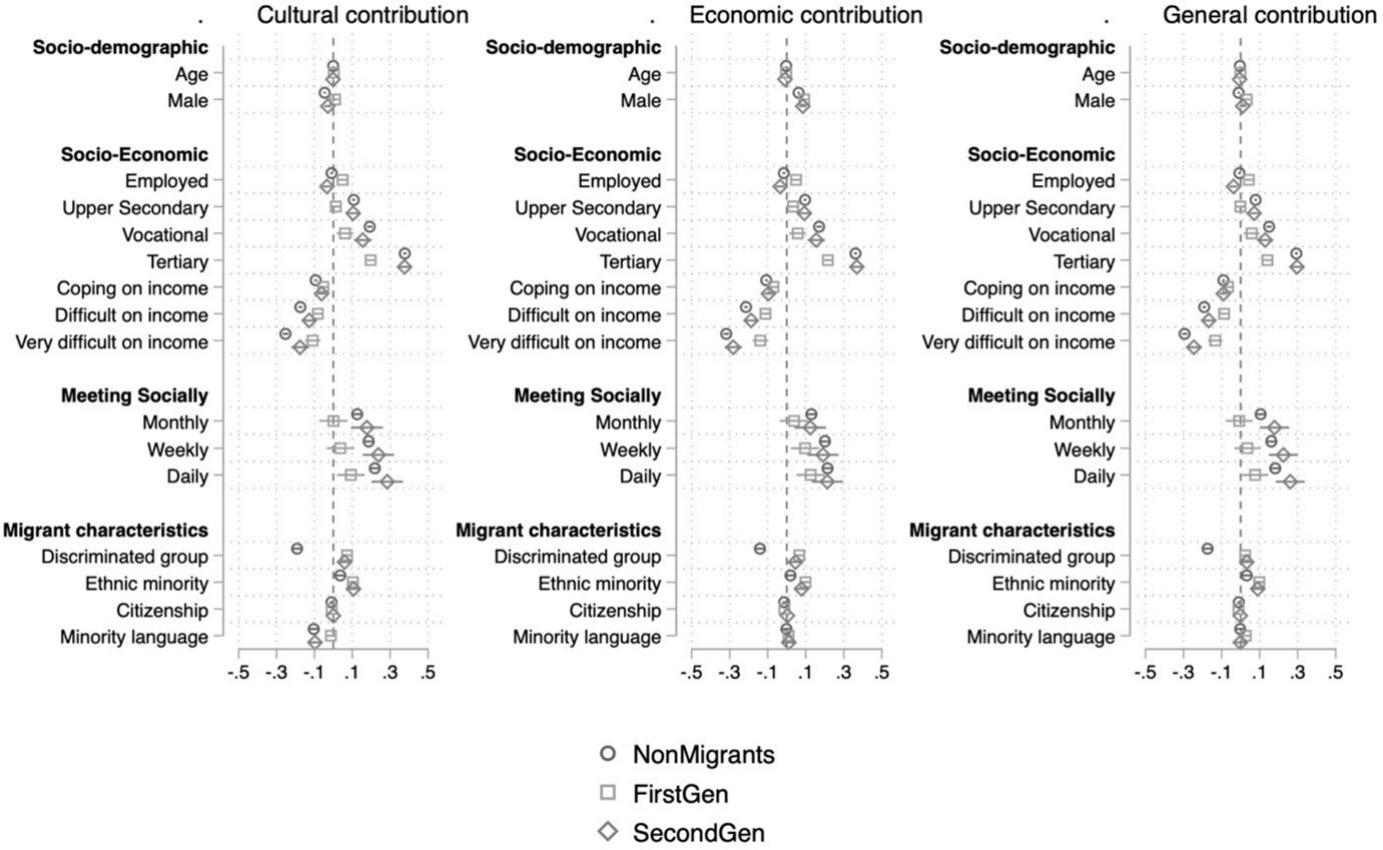
Determinants of attitudes toward the effect of immigration among non-migrants, first-generation immigrants and second-generation immigrants

Models 1 and 2 in Tables 1 and 2 show that younger, more educated, more religious respondents who do not have income difficulties, hold residing country’s citizenship and socialize more hold significantly more pro-immigration attitudes. Those who are in paid work are significantly less likely to be in favor of allowing immigrants to come into the country and also view immigration’s effect on the economy significantly more negatively. However, as shown in Figs. 3 and 4 and Tables A11-A16 in Online Appendix, these effects are driven by non-migrants and the second generation entirely. In short, those in paid work among non-migrants and second-generation immigrants are significantly more negative toward immigration compared to those non-working within their respective groups. On the contrary, working first-generation migrants are significantly more positive toward immigration compared to non-working first-generation immigrants. Women are significantly more willing to allow immigrants of different races and from poorer countries to come compared to men. In contrast, while women also evaluate immigration’s effect on the country’s culture more positively, men view immigration’s effect on the economy significantly more positively. There are also significant differences between the association of respondents’ religious denominations and individuals’ attitudes. While Roman Catholics, Protestants and Eastern Orthodox church members are significantly more negative in their attitudes compared to non-religious respondents, Muslims^13^ and the followers of Eastern religions are significantly more positive.

We now turn to minority-related characteristics. In order to disentangle the effects of these variables to the fullest, we split the sample into three groups based on the immigrant generation and rerun the models within the split samples as shown in Figs. 3 and 4. This allows us to disentangle the effect of specific characteristics we hypothesized would work differently for different samples. For all dependent variables, respondents who have an immigration background and subjectively feel part of a discriminated group (in terms of nationality, religion, race, ethnicity and language) are significantly more positive toward immigration. Contrarily, non-migrants who feel discriminated against are significantly more negative toward immigration. This is fully consistent with our hypotheses H3a and H3b.

Although we hypothesized that being part of an ethnic minority would have a differential effect on non-migrants compared to immigrants, the data has not supported this expectation. First- and second-generation immigrants who are part of an ethnic minority are significantly more positive toward immigration compared to those who are not a minority (confirming our hypothesis H3a based on the intergroup solidarity framework). However, the analysis shows that even among non-migrants those who are part of an ethnic minority are significantly more positive toward immigration compared to ethnic majorities.

The story appears slightly more complicated for those who speak a minority language at home. For the three dependent variables that measure attitudes to open borders, those non-migrants who speak foreign languages at home are significantly more negative toward allowing immigrants of different races and from poorer countries,^14^ while there is no significant language effect among first- and second-generation immigrants. Nevertheless, those who speak a foreign language at home among non-migrants and second-generation migrants are significantly more negative regarding their opinion of immigration’s contribution to the country’s culture. There is no effect of speaking a foreign language at home on attitudes toward immigration’s effect on the economy for none of the samples. Finally, those first-generation immigrants who speak a foreign language at home are significantly more likely to view immigration’s overall effect on the country positively compared to those who do not, while there is no significant effect of language for non-migrants and second-generation immigrants.

Our final research question asked whether first-generation immigrants' attitudes converge to those of non-migrants’ the longer they stay in the country. We hypothesized that as more years pass, the first generation’s attitudes toward immigration would become more different to those recently arriving, which would be, considering our findings, more negative. Table 4 shows the results of a multilevel regression for the sample of first-generation migrants. After controlling for all the relevant variables, first-generation immigrants become significantly more negative compared to those that came to the country last year (the reference category) only after 11 or more years in the country (this is valid for allowing immigrants of the same and different race and for attitudes regarding immigration’s effect on the economy). The same effect can be found for immigrants staying 21 years or more in the country (this group is also significantly more negative regarding immigration’s effect in general). Interestingly, there is no significant difference in attitudes toward those from poorer countries nor toward immigration’s effect on culture. Moreover, as Table 4 shows, most immigrants from other parts of the world are significantly more negative toward immigration compared to those from old EU countries. Nevertheless, these effects differ based on the attitude in question. Although we consider, overall, hypothesis H4 confirmed, these results once again highlight the necessity to distinguish between different attitudes when investigating immigrants’ attitudes toward immigration. We also recognize that a longitudinal data would be more fitting to confirm H4, as cross-sectional data might be biased by changing trends in migration. Nevertheless, considering the lack of international longitudinal data collection on immigrants, we believe that our approach, to include varied control variables and fixed effects controls in the model, is able to capture the association between the time spent in the country and immigration attitudes.

**Table 4:** Multilevel hierarchical regression coefficients for the six dependent variables for the first-generation immigrants, including years since arrival and region of origin as independent variables in the model

	Allow poor	Allow different	Allow same	Effect on culture	Effect on economy	General effect
Age	− 0.013	− 0.008	− 0.005	0.003	0.001	0.002
	(0.002)**	(0.002)**	(0.002)**	(0.002) +	(0.002)	(0.002)
Age squared	0.000	0.000	0.000	− 0.000	− 0.000	− 0.000
	(0.000)**	(0.000)	(0.000)	(0.000)**	(0.000)	(0.000)**
Male	− 0.011	0.018	0.020	0.008	0.092	0.030
	(0.012)	(0.011)+	(0.010)+	(0.010)	(0.010)**	(0.009)**
In paid work	0.045	0.037	0.007	0.051	0.053	0.047
	(0.013)**	(0.013)**	(0.012)	(0.011)**	(0.011)**	(0.011)**
Secondary education	− 0.022	0.014	0.035	0.015	0.030	− 0.002
*(Ref Elementary)*	(0.015)	(0.014)	(0.014)*	(0.012)	(0.013)*	(0.012)
Vocational education	− 0.016	0.038	0.075	0.061	0.051	0.059
	(0.027)	(0.026)	(0.024)**	(0.022)**	(0.023)*	(0.021)**
Tertiary education	0.163	0.202	0.191	0.194	0.201	0.132
	(0.016)**	(0.015)**	(0.014)**	(0.013)**	(0.013)**	(0.012)**
Coping on income	− 0.066	− 0.067	− 0.074	− 0.052	− 0.070	− 0.068
*(Ref Satisfied)*	(0.014)**	(0.014)**	(0.013)**	(0.012)**	(0.012)**	(0.011)**
Difficult on income	− 0.074	− 0.108	− 0.117	− 0.084	− 0.115	− 0.091
	(0.018)**	(0.017)**	(0.016)**	(0.015)**	(0.015)**	(0.014)**
Very difficult on income	− 0.106	− 0.113	− 0.148	− 0.110	− 0.141	− 0.136
	(0.025)**	(0.023)**	(0.022)**	(0.021)**	(0.021)**	(0.019)**
Monthly socializing	0.149	0.046	0.103	0.006	0.044	− 0.002
*(Ref Never)*	(0.046)**	(0.043)	(0.041)*	(0.038)	(0.039)	(0.036)
Weekly socializing	0.163	0.075	0.122	0.044	0.106	0.046
	(0.045)**	(0.043)+	(0.040)**	(0.037)	(0.038)**	(0.035)
Daily socializing	0.217	0.139	0.180	0.101	0.142	0.091
	(0.045)**	(0.043)**	(0.041)**	(0.037)**	(0.038)**	(0.035)**
Discriminated	**0.097**	**0.093**	**0.069**	**0.071**	**0.068**	**0.024**
*(Ref Non-discrim)*	(0.017)**	(0.016)**	(0.015)**	(0.014)**	(0.014)**	**(0.013)+**
Minority	**0.042**	**0.068**	**0.046**	**0.094**	**0.090**	**0.082**
*(Ref Non-minor)*	(0.014)**	(0.013)**	(0.013)**	(0.012)**	(0.012)**	(0.011)**
Citizenship	**− 0.006**	**− 0.005**	**− 0.005**	**− 0.007**	**− 0.008**	**− 0.008**
	(0.001)**	(0.001)**	(0.001)**	(0.001)**	(0.001)**	(0.001)**
Roman Catholic	− 0.100	− 0.087	− 0.025	− 0.106	− 0.043	− 0.081
*(Ref No religion)*	(0.017)**	(0.016)**	(0.015)	(0.014)**	(0.014)**	(0.013)**
Protestant	− 0.065	− 0.061	0.023	− 0.094	− 0.047	− 0.068
	(0.022)**	(0.021)**	(0.020)	(0.019)**	(0.019)*	(0.018)**
Eastern Orthodox	− 0.106	− 0.086	− 0.052	− 0.098	− 0.065	− 0.075
	(0.023)**	(0.022)**	(0.020)*	(0.019)**	(0.019)**	(0.018)**
Other Christian religions	− 0.012	− 0.042	0.021	− 0.083	− 0.057	− 0.098
	(0.037)	(0.035)	(0.034)	(0.031)**	(0.031)+	(0.029)**
Other Non-Christian religions	0.007	− 0.004	− 0.030	− 0.118	− 0.004	− 0.079
	(0.053)	(0.051)	(0.049)	(0.045)**	(0.045)	(0.043)+
Islam	0.077	0.067	0.028	0.091	0.065	0.085
	(0.023)**	(0.022)**	(0.021)	(0.019)**	(0.019)**	(0.018)**
Eastern Religions	− 0.053	− 0.038	− 0.057	− 0.025	− 0.004	− 0.049
	(0.044)	(0.041)	(0.040)	(0.036)	(0.037)	(0.034)
Religiosity	0.007	0.003	− 0.002	0.012	0.006	0.014
	(0.002)**	(0.002)	(0.002)	(0.002)**	(0.002)**	(0.002)**
Language spoken at home	**− 0.005**	**0.003**	**0.014**	**− 0.006**	**0.005**	**0.021**
	**(0.016)**	**(0.015)**	**(0.014)**	**(0.013)**	**(0.013)**	**(0.012)+**
1–5 years in destination	**0.015**	**− 0.103**	**− 0.079**	**− 0.024**	**− 0.061**	**0.017**
*(Ref Came last year)*	**(0.056)**	**(0.054)+**	**(0.051)**	**(0.046)**	**(0.047)**	**(0.045)**
6–10 years in destination	**0.004**	**− 0.122**	**− 0.099**	**− 0.016**	**− 0.069**	**− 0.009**
	**(0.056)**	(0.054)*	**(0.051)+**	**(0.047)**	**(0.048)**	**(0.045)**
11–20 years in destination	**− 0.010**	**− 0.142**	**− 0.138**	**− 0.038**	**− 0.135**	**− 0.040**
	**(0.056)**	(0.053)**	(0.051)**	**(0.046)**	(0.047)**	**(0.045)**
21 + years in destination	**− 0.033**	**− 0.177**	**− 0.179**	**− 0.072**	**− 0.221**	**− 0.107**
	(0.057)	(0.054)**	(0.052)**	(0.047)	(0.048)**	(0.045)*
New EU	− 0.129	− 0.125	− 0.095	− 0.048	0.006	− 0.003
*(Ref Old EU)*	(0.019)**	(0.018)**	(0.017)**	(0.016)**	(0.016)	(0.015)
Global North and Australia	− 0.146	− 0.127	− 0.097	− 0.087	− 0.062	− 0.043
	(0.026)**	(0.025)**	(0.023)**	(0.022)**	(0.022)**	(0.021)*
Indian Subcontinent	− 0.038	− 0.036	− 0.086	− 0.028	− 0.028	0.071
	(0.040)	(0.038)	(0.036)*	(0.033)	(0.034)	(0.031)*
Asia + Oceania	− 0.071	− 0.029	− 0.153	0.001	− 0.017	0.084
	(0.034)*	(0.032)	(0.031)**	(0.028)	(0.028)	(0.027)**
Africa	0.043	0.011	− 0.075	0.047	0.074	0.050
	(0.024)+	(0.023)	(0.022)**	(0.020)*	(0.020)**	(0.019)**
Middle East	− 0.078	− 0.106	− 0.194	− 0.035	− 0.080	0.033
	(0.028)**	(0.027)**	(0.025)**	(0.023)	(0.024)**	(0.022)
South America	0.027	− 0.035	− 0.121	0.099	0.034	0.100
	(0.030)	(0.028)	(0.027)**	(0.024)**	(0.025)	(0.023)**
Balkan	− 0.091	− 0.086	− 0.140	− 0.049	− 0.031	− 0.004
	(0.027)**	(0.026)**	(0.024)**	(0.022)*	(0.023)	(0.021)
Central Asia	− 0.181	− 0.179	− 0.104	− 0.114	− 0.057	− 0.019
	(0.041)**	(0.039)**	(0.037)**	(0.034)**	(0.035)+	(0.032)
Year 2004	− 0.018	0.059	0.046	− 0.045	− 0.051	− 0.029
*(Ref year 2002)*	(0.043)	(0.042)	(0.036)	(0.033)	(0.036)	(0.037)
Year 2006	− 0.093	− 0.025	0.045	− 0.069	0.006	0.006
	(0.043)*	(0.042)	(0.036)	(0.033)*	(0.036)	(0.038)
Year 2008	− 0.051	0.043	0.067	− 0.045	− 0.027	0.031
	(0.041)	(0.040)	(0.034)*	(0.032)	(0.034)	(0.036)
Year 2010	− 0.070	0.043	0.057	− 0.083	− 0.045	− 0.001
	(0.041)+	(0.040)	(0.034)+	(0.031)**	(0.034)	(0.035)
Year 2012	− 0.036	0.049	0.045	− 0.021	0.012	0.049
	(0.042)	(0.041)	(0.034)	(0.032)	(0.035)	(0.036)
Year 2014	− 0.127	0.069	0.088	− 0.123	− 0.072	0.000
	(0.041)**	(0.040)+	(0.034)**	(0.032)**	(0.035)*	(0.036)
Year 2016	− 0.046	0.014	0.079	− 0.148	− 0.039	− 0.009
	(0.042)	(0.041)	(0.035)*	(0.033)**	(0.036)	(0.037)
Year 2018	− 0.007	0.072	0.135	− 0.115	0.046	0.032
	(0.040)	(0.039)+	(0.033)**	(0.031)**	(0.034)	(0.035)
Intercept	3.029	3.120	3.215	2.872	2.720	2.669
	(0.098)**	(0.094)**	(0.085)**	(0.079)**	(0.079)**	(0.077)**
Country	− 1.662	− 1.688	− 2.010	− 2.020	− 2.118	− 2.014
	(0.172)**	(0.170)**	(0.174)**	(0.174)**	(0.175)**	(0.172)**
Period	− 2.500	− 2.508	− 2.779	− 2.822	− 2.678	− 2.577
	(0.116)**	(0.113)**	(0.133)**	(0.131)**	(0.114)**	(0.101)**
Individual	− 0.209	− 0.260	− 0.305	− 0.391	− 0.381	− 0.449
	(0.005)**	(0.005)**	(0.005)**	(0.005)**	(0.005)**	(0.005)**
	21,457	21,454	21,500	21,602	21,439	21,322

alpha(0.01, 0.05, 0.1) symbol(**, *,+)

Finally, as robustness checks, we also use 2 indices of attitudes toward immigration, both consisting of 3 items, one measuring attitudes toward immigration policy and one attitude toward the effects of immigration. Lastly, we include an analysis of a singular index in which we combined all six items. We created the indices by combining answers for all individuals who answered at least two or four questions, respective to the index, and computing an average value of their non-missing answers.^15^ The results for these indices can be found in Figure A1 and Tables A19-A21 in Online Appendix. The analysis revealed that broad results are generally consistent across different operationalizations of attitudes. However, nuances in the strength (and occasionally in the orientation) of the estimated effects of the explanatory factors for separate dimensions of attitudes and the indices are present. Some effects of the explanatory factors disappear in the indexed measures. This points to the importance of distinguishing between different immigration attitudes when analyzing factors affecting them (Dražanová et al., 2022), especially for non-migrant samples.

## Discussion

Attitudes toward immigration are typically investigated through the majority/non-migrant perspective. The literature examining immigrants’ support or opposition to immigration is scarce. Given the growing diversity of European societies and the broadening rights of minorities in their host countries when former immigrants typically have an equal vote in designing the immigration policy of tomorrow, research on attitudes toward immigration should not be restricted to the majority’s views as it is insufficient to systematically describe the countries’ populations (Ramos et al., 2019). We argue that a distinction can also be made among individuals with different migratory backgrounds between those who are part of an ethnic minority–majority, those who speak a non-national language at home and those who perceive themselves as being discriminated against. Moreover, subcategories exist among immigrants, such as between first- and second-generation immigrants and between those with citizenship in the residence country. Accordingly, we argue it is necessary to go beyond the non-migrant in-group vs. immigrant out-group distinction when studying attitudes toward immigration. Conventional concepts and methods in migration studies do not necessarily reflect the complex diverse reality of multicultural societies. In this paper, we address these needs in several ways.

Firstly, we investigate the effect of migratory background on attitudes toward immigration compared to the non-migrant population. Secondly, we analyze the association between attitudes and individual characteristics, particularly those associated with more or less stigmatization (such as perceived unfair treatment, being an ethnic minority or speaking non-national languages). Thirdly, we present variations in these associations across migratory backgrounds and across different types of attitudes. Lastly, we establish whether immigration attitudes of the first generation change the longer they stay in the country and whether this effect is distinct across diverse immigration attitudes. Our study extends theoretical frameworks on immigration attitudes by explicitly comparing the roles of intergroup solidarity and threat perceptions among first-generation migrants, second-generation migrants and non-migrants across multiple European countries. Unlike previous research, we focus on how minority-specific factors, such as perceived discrimination and linguistic diversity, operate differently across these groups.

When studying minority attitudes, previous studies predominantly employ data from Western Europe and the USA (Just & Anderson, 2015; Gonnot & Lo Polito, 2021, Becker, 2019. Neureiter & Schulte, 2024). In our research, we aimed to show the importance of broadening these perspectives. Thus, we analyzed data from countries across Europe including Southern and Eastern European countries. Especially after the Russian invasion of Ukraine, “non-traditional” destination countries such as Poland and Czechia have been facing unprecedented numbers of refugees. It is crucial to broaden our understanding of minority attitudes beyond Western European countries. Our study is among the first to employ a multicountry, multilevel analysis that spans 20 European countries, including Eastern and Southern Europe, providing a comprehensive and comparative perspective. Nevertheless, due to the comparative nature of our research design, we cannot provide detailed results for each country. We also included a broad range of individual minority-related characteristics, therefore allowing a comparison of how these factors affect non-migrant and migrant minorities’ attitudes. Finally, we distinguish between various dimensions of immigration attitudes (e.g., immigration policy, perceived economic impact) rather than relying on composite indices, revealing nuanced patterns and documenting the importance of analyzing dimensions of attitudes toward immigration separately.

We show immigrants’ attitudes toward immigration are on average more favorable compared to non-migrants’ attitudes. However, they tend to become more negative with time spent in the country, when we compare first-generation immigrants considering their tenure in the destination (Table 4). Specifically, for the first generation, considering six measures of attitudes separately, we show empirically the negative effect is differently paced with some attitudes (such as to open borders) converging faster than opinions on immigration’s effect on culture, a finding that nuances previous studies like Neureiter and Schulte (2024). Although still significantly more positive compared to non-migrants, second-generation migrants are less favorable to immigration than first generation (Tables 1 and 2). Our findings also highlight that while solidarity drives pro-immigration attitudes among first-generation migrants, its effects weaken for the second generation, with significant variation observed across national contexts. This contributes to the broader understanding of generational shifts in immigration attitudes, their context dependency and the implications for social cohesion.

Even modest differences in attitudes between groups can have significant policy and societal implications, particularly as migrant populations grow and their political and social influence increases. Understanding these nuances is essential for fostering social cohesion and informing evidence-based policymaking. The country-specific variation observed in Figs. 1 and 2 emphasizes the importance of contextualizing attitudes toward immigration. While aggregate differences between migrant generations and non-migrants may be relatively modest, the heterogeneity across countries suggests that national conditions—such as economic structures, social norms or integration policies—may influence these relationships. Though we do not explicitly test for the mechanisms driving this variation, these findings highlight the need for further research into the country-level factors that shape public attitudes toward immigration. Understanding these dynamics is crucial for designing policies that promote social cohesion in diverse national contexts.

The determinants of attitudes toward immigration differ for the three groups and across different dimensions of attitudes. Minority-related characteristics such as the language spoken at home and experience of discrimination differ in their effect according to one’s migratory background. While speaking a minority language and perceptions of discrimination are associated with more favorable attitudes among immigrants, it leads to less favorable attitudes toward immigration for non-migrants and in one dimension also for the second generation (Figs. 3 and 4). These findings are in line with explanations of anti-immigration sentiments as a reaction against cultural changes that threaten the worldview of once-predominant sectors of the population (Norris & Inglehart, 2019).

Our results support existing findings regarding the differences among immigrant generations (Becker, 2019) and the convergence of attitudes with the time spent in the country (Gonnot & Lo Polito, 2021). Similarly, our analysis confirms the estimated relationship between acquiring citizenship and less favorable immigration attitudes (Just & Anderson, 2015). Thus, we confirm previous studies’ validity in new contexts. We uncovered a variation in the direction of estimated associations between membership in ethnic and linguistic minority groups and perceived discrimination and attitudes for the migrant and non-migrant subsamples. This confirms the importance of diversifying the research on immigration attitudes to include minority and migrant samples to achieve a complex understanding of European populations’ opinions. Our further contribution is the comparison of different dimensions, which shows that the use of indices when studying immigration attitudes can yield inconsistent results for different subpopulations.

These findings, given that attitudes can influence behavior (Malloy et al., 2022), contribute to our understanding of the social and political actions of immigrants, including their voting patterns and support for various political parties. In the last decade, attitudes to immigration represent an important cleavage in European politics. Understanding variation in these attitudes also among subpopulations is therefore important. For example, Malloy et al. (2022) show that more pro-immigration individuals have a higher likelihood of voting for parties that are further left on the political spectrum, while those with anti-immigration views prefer parties further to the right. Since first- and especially second-generation immigrants form a potentially sizable electorate in many European countries, it is crucial to understand their immigration attitudes. On the other hand, since we show that generation membership and lengths of residency across immigrants can significantly decrease their support for immigration, our findings challenge the idea that greater diversity will universally lead to more welcoming attitudes toward immigrants in European societies. Nevertheless, these effects go both directions. (Nandi & Luthra, 2021) show how electoral events in turn impact immigrants and minorities. For example, Brexit exacerbated already higher levels of perceived discrimination among higher-educated minorities in the UK while reducing the buffering effect of residence in less socioeconomically deprived areas with lower levels of previous right-wing party support (Nandi & Luthra, 2021). It is therefore highly important for policymakers to understand the attitudes to immigration of first- and second-generation migrants across Europe and their determinants. This could help to create targeted educational campaigns and other intervention programs that could potentially prevent radicalization.

Arguably, due to the design of the used data, our study is observational, and therefore, our findings show associations. It is also worth mentioning that international surveys often use immigration question framings based on the assumption that the respondents are from the racial/religious majority group. Despite these limitations, our research explains variations in the migrants’ and non-migrants’ attitudes and delivers valuable information for the future research design for the analysis of anti-immigrant attitudes dimensions. Future research using longitudinal data on attitudes can further causally test the associations presented here. Moreover, to increase the internal validity of the analysis data including boosted samples of individuals from specific countries or regions would be needed.

Lastly, we recognize the role of media and social media forming individuals’ opinions and attitudes, including immigration attitudes (Erhard et al., 2021; Harteveld et al., 2018; Nasuto & Rowe, 2024), as well as importance of social media space in expressing these attitudes (Ekman, 2019; Rowe et al., 2021). Our current research design and data employed do not allow to control for individual’s use and consumption of media and social media content. Including these dimensions of attitudes' determinants will be a welcomed avenue of future research, which can build on our results. Similarly, we would also like to acknowledge that thanks to the salience of immigration discussion on social media, digital traces represent a valuable source of data for research on attitudes toward immigrants, which is currently on raise (Nasuto & Rowe, 2024; Freire-Vidal et al., 2021). The use of digital traces is complementary to the research utilizing survey data (such as this study), which on the one hand offer more information about respondent expressing their attitudes, however, could not provide the temporal and geographical granularity and real-time accessibility of data gathered from social media.

## Supplementary Information

Below is the link to the electronic supplementary material.

Supplementary file1 (DOCX 287 KB)

## Data Availability

We confirm that data and replication materials will be made publicly available if this article is accepted for publication.

## Footnotes

1 See Data and Method section for the definition of migrants in this study.

2 Being an ethnic minority most likely means that some generations ago these individuals’ ancestors have migrated. However, this could have happened even centuries ago. Moreover, some ethnic minorities did not migrate, but what migrated were country boarders. This is the case, for example, of Hungarians in the Transylvania region of Romania, Austrians in the North of Italy, etc.

3 Arguably, we do not know from our data whether these individuals are third-generation immigrants or people with a foreign partner. Nevertheless, within Europe, we assume that these are autochthonous minorities living in regions that were possibly moved across borders throughout history, such as the Hungarian minority within Slovakia or the German-speaking minority in the North of Italy. These also include the Romani speakers throughout Europe. The methodology section provides more detailed description how these foreign speakers have been selected.

4 All survey respondents with missing data on their or their parents place of birth are excluded from the sample because it is impossible to categorize them.

5 Our measure of a perceived discrimination is based on a dichotomous measure combining whether the respondent replied they feel like they belong to the discriminated group in at least one of the following categories: nationality, religion, race, ethnicity and language.

6 ESS asks about the first and second language spoken at home. Out of these two variables we coded those speaking at least one official national language at home as those speaking the majority language. We consider an official language would be a language recognized on the whole territory as a second language. Therefore, in our dataset non-migrants speaking a minority language could be third-generation migrants, but also minorities whose languages are not (fully) recognized by their country of residence. To sum up, we consider everybody linguistically recognized by the national government as speaking an official language and the rest are minority language speakers.

7 In total, 5832 individuals (2.62%) of non-migrants speak a non-official national language, which represents 40% of all non-official national speakers in the whole sample. On the other hand, 1.45% (3226 individuals) of non-migrants see themselves as a minority member, which is 24,66% of minority members in the whole sample.

8 Categories are divided into bigger geographical units such as *the Middle East* in lieu of countries of origin due to low numbers of observations per country of origin.

9 All three-level models offer a significantly better fit to the data compared to single-level models. The likelihood ratio (LR) test which compared the current three-level models to single-level models with no country and no country-period effects (i.e., linear regression) were all significant. They were general threat (χ^2^ = 26,038.81, *p* < 0.0001), economic threat (χ^2^ = 18,793.89, *p* < 0.0001), cultural threat (χ^2^ = 31,682.60, *p* < 0.0001), same ethnicity (χ^2^ = 28,163.94, *p* < 0.0001), from poorer countries (χ^2^ = 31,334.46, *p* < 0.0001), different ethnicity (χ^2^ = 31,072.26, *p* < 0.0001), respectively.

10 It is interesting to note that even with this low degree of clustering the three-level model was significantly preferred to the single-level model and each of its two-level counterparts.

11 This is calculated by summing the beta coefficient for the first generation and the first generation’s random variance, i.e., ß_1_ + *û*_1_ = 0.162 + 0.022 = 0.184.

12 As was the case with the first generation, this is calculated by summing the beta coefficient for second generation and the second generation’s random variance, i.e., ß_2_ + *û*_2_ = 0.108 + 0.002 = 0.11.

13 The only indicators where there are, in fact, negative but statistically nonsignificant differences in attitudes toward immigration between Muslims and atheists is the question asking whether the respondent would allow immigrants from the same race to come and live into the country. The result is most likely due to the fact that the question refers to the country’s majority race; thus, Muslims are likely answering a question about a different race than themselves.

14 They are also significantly opposed to allowing immigrants of the same race, albeit only at the 0.1 level of significance.

15 See Appendix Table A17 and A18 for the correlation and factor analyses and Cronbach’s alphas.
