# Supplementary material for: Migrant and Non-migrant Views on Immigration in Europe
Source: Eur J Popul. 2025 Jun 2;41(1):13. doi: 10.1007/s10680-025-09736-8 (PMC12130419; doi:10.1007/s10680-025-09736-8)
Supplement: Supplementary file 1 — Supplementary file1 (DOCX 287 KB) [file 10680_2025_9736_MOESM1_ESM.docx]

**Online Appendix**

Table of contents

[Appendix 1. Coding, descriptive statistics of variables, and robustness checks 3](#_Toc185341263)

[Table A1. Number and percentage of respondents per country in each immigrant generation including cases with missing values. 3](#_Toc185341264)

[Table A2. Number of respondents per country in each ESS round 4](#_Toc185341265)

[Table A3. Number of non-migrant respondents per country in each ESS round 5](#_Toc185341266)

[Table A4. Number of first-generation immigrant respondents per country in each ESS round 6](#_Toc185341267)

[Table A5. Number of second-generation immigrant respondents per country in each ESS round 7](#_Toc185341268)

[Table A6. Dependent variable coding 8](#_Toc185341269)

[Table A7. Independent variables coding 9](#_Toc185341270)

[Table A8. Descriptive statistics (mean, standard deviation, minimum and maximum) of variables 10](#_Toc185341271)

[Table A9a. Results for a three-level multilevel hierarchical model for attitudes regarding open borders in the full sample 12](#_Toc185341272)

[Table A9b. Results for a regression model including fixed effect country and country/period variable for attitudes regarding open borders in the full sample. 14](#_Toc185341273)

[Table A10a. Results for a three-level multilevel hierarchical model for attitudes regarding the effects of immigration in the full sample 15](#_Toc185341274)

[Table A10b. Results for a regression model including fixed effect country and country/period variable for attitudes regarding effect on immigration in the full sample. 17](#_Toc185341275)

[Table A10c. Results for a three-level multilevel hierarchical model for attitudes regarding the effects of immigration and the attitudes regarding open borders with no controls. 19](#_Toc185341276)

[Table A11. Multilevel hierarchical regression coefficients for the dependent variable “allow immigrants of different race” for the three separate samples 20](#_Toc185341277)

[Table A12. Multilevel hierarchical regression coefficients for the dependent variable “allow immigrants of same race” for the three separate samples 22](#_Toc185341278)

[Table A13. Multilevel hierarchical regression coefficients for the dependent variable “allow immigrants from poor countries” for the three separate samples 24](#_Toc185341279)

[Table A14. Multilevel hierarchical regression coefficients for the dependent variable “immigration effect on country in general” for the three separate samples 26](#_Toc185341280)

[Table A15. Multilevel hierarchical regression coefficients for the dependent variable “immigration effect on economy” for the three separate samples 28](#_Toc185341281)

[Table A16. Multilevel hierarchical regression coefficients for the dependent variable “immigration effect on culture” for the three separate samples 30](#_Toc185341282)

[Figure A1. Determinants of attitudes to immigration within the three samples with different operationalization of the dependent variables 32](#_Toc185341283)

[Table A17. Correlation analysis of six measures of attitudes towards immigrants 33](#_Toc185341284)

[Table A18. Factor analysis (including factor loadings and rotated factor loadings) of six measures of attitudes towards immigrants 34](#_Toc185341285)

[Table A19. Multilevel hierarchical regression coefficients for the index “attitude toward the effects of immigration” for the three separate samples 35](#_Toc185341286)

[Table A20. Multilevel hierarchical regression coefficients for the index “attitude toward open borders” for the three separate samples 37](#_Toc185341287)

[Table A21. Multilevel hierarchical regression coefficients for the index of all six measures of attitudes for the three separate samples 39](#_Toc185341288)

# **Appendix 1. Coding, descriptive statistics of variables, and robustness checks**

## **Table A1.** Number and percentage of respondents per country in each immigrant generation including cases with missing values.

| **Country** | **Non-migrants** | **1st generation** | **2nd generation** | **Missing data on background** | **Total** |
| --- | --- | --- | --- | --- | --- |
| **Austria** | 10703 | 1172 | 1197 | 150 | 13222 |
| **%** | 80.95 | 8.86 | 9.05 | 1.13 | 100.00 |
| **Belgium** | 12595 | 1694 | 1602 | 219 | 16110 |
| **%** | 78.18 | 10.52 | 9.94 | 1.36 | 100.00 |
| **Switzerland** | 9814 | 3340 | 2105 | 143 | 15402 |
| **%** | 63.72 | 21.69 | 13.67 | 0.93 | 100.00 |
| **Czechia** | 15752 | 408 | 1148 | 306 | 17614 |
| **%** | 89.43 | 2.32 | 6.52 | 1.74 | 100.00 |
| **Germany** | 21029 | 2250 | 2099 | 322 | 25700 |
| **%** | 81.82 | 8.75 | 8.17 | 1.25 | 100.00 |
| **Denmark** | 10891 | 692 | 661 | 164 | 12408 |
| **%** | 87.77 | 5.58 | 5.33 | 1.32 | 100.00 |
| **Estonia** | 9583 | 2480 | 2777 | 474 | 15314 |
| **%** | 62.58 | 16.19 | 18.13 | 3.10 | 100.00 |
| **Finland** | 16948 | 486 | 323 | 198 | 17955 |
| **%** | 94.39 | 2.71 | 1.80 | 1.10 | 100.00 |
| **France** | 13261 | 1519 | 1983 | 298 | 17061 |
| **%** | 77.73 | 8.90 | 11.62 | 1.75 | 100.00 |
| **UK** | 15907 | 2047 | 1540 | 336 | 19830 |
| **%** | 80.22 | 10.32 | 7.77 | 1.69 | 100.00 |
| **Greece** | 8225 | 724 | 615 | 195 | 9759 |
| **%** | 84.28 | 7.42 | 6.30 | 2.00 | 100.00 |
| **Croatia** | 3975 | 430 | 454 | 84 | 4943 |
| **%** | 80.42 | 8.70 | 9.18 | 1.70 | 100.00 |
| **Ireland** | 16766 | 2217 | 826 | 654 | 20463 |
| **%** | 81.93 | 10.83 | 4.04 | 3.20 | 100.00 |
| **Lithuania** | 8907 | 270 | 612 | 204 | 9993 |
| **%** | 89.13 | 2.70 | 6.12 | 2.04 | 100.00 |
| **Latvia** | 1987 | 349 | 499 | 63 | 2898 |
| **%** | 68.56 | 12.04 | 17.22 | 2.17 | 100.00 |
| **Netherlands** | 14210 | 1316 | 1153 | 180 | 16859 |
| **%** | 84.29 | 7.81 | 6.84 | 1.07 | 100.00 |
| **Norway** | 12595 | 1230 | 666 | 163 | 14654 |
| **%** | 85.95 | 8.39 | 4.54 | 1.11 | 100.00 |
| **Portugal** | 14538 | 731 | 379 | 395 | 16043 |
| **%** | 90.62 | 4.56 | 2.36 | 2.46 | 100.00 |
| **Sweden** | 12638 | 1791 | 1354 | 146 | 15929 |
| **%** | 79.34 | 11.24 | 8.50 | 0.92 | 100.00 |
| **Slovenia** | 9934 | 821 | 1075 | 402 | 12232 |
| **%** | 81.21 | 6.71 | 8.79 | 3.29 | 100.00 |
| **Total** | 240258 | 25967 | 23068 | 5096 | 294389 |
| **%** | 81.61 | 8.82 | 7.84 | 1.73 | 100.00 |
|  | | | | | |

*Note: First row has frequencies and second row has percentages across immigrant generations within country.*

## Table A2. Number of respondents per country in each ESS round

|  | **ESS round** | | | | | | | | | |
| --- | --- | --- | --- | --- | --- | --- | --- | --- | --- | --- |
| **Country** | **1** | **2** | **3** | **4** | **5** | **6** | **7** | **8** | **9** | **Total** |
| **Austria** | 2026 | 2084 | 2223 | 0 | 0 | 0 | 1739 | 1934 | 2398 | 12404 |
| **%** | 7.27 | 7.81 | 8.92 | 0.00 | 0.00 | 0.00 | 5.73 | 6.72 | 7.55 | 4.67 |
| **Belgium** | 1661 | 1706 | 1737 | 1692 | 1664 | 1809 | 1714 | 1716 | 1703 | 15402 |
| **%** | 5.96 | 6.39 | 6.97 | 5.25 | 5.08 | 5.97 | 5.65 | 5.96 | 5.36 | 5.80 |
| **Croatia** | 0 | 0 | 0 | 1329 | 1453 | 0 | 0 | 0 | 1718 | 4500 |
| **%** | 0.00 | 0.00 | 0.00 | 4.12 | 4.43 | 0.00 | 0.00 | 0.00 | 5.41 | 1.69 |
| **Czechia** | 1195 | 2416 | 0 | 1869 | 2261 | 1647 | 1860 | 2174 | 2164 | 15586 |
| **%** | 4.29 | 9.05 | 0.00 | 5.80 | 6.90 | 5.43 | 6.13 | 7.55 | 6.81 | 5.87 |
| **Denmark** | 1412 | 1434 | 1407 | 1510 | 1523 | 1590 | 1463 | 0 | 1499 | 11838 |
| **%** | 5.07 | 5.37 | 5.65 | 4.68 | 4.65 | 5.24 | 4.82 | 0.00 | 4.72 | 4.46 |
| **Estonia** | 0 | 1837 | 1327 | 1357 | 1701 | 2195 | 1911 | 1939 | 1861 | 14128 |
| **%** | 0.00 | 6.88 | 5.33 | 4.21 | 5.19 | 7.24 | 6.29 | 6.74 | 5.86 | 5.32 |
| **Finland** | 1934 | 0 | 1833 | 2152 | 1830 | 2147 | 2041 | 1863 | 1670 | 15470 |
| **%** | 6.94 | 0.00 | 7.36 | 6.68 | 5.58 | 7.08 | 6.72 | 6.47 | 5.26 | 5.82 |
| **France** | 0 | 0 | 1862 | 1956 | 1638 | 1895 | 1833 | 1966 | 1897 | 13047 |
| **%** | 0.00 | 0.00 | 7.47 | 6.07 | 5.00 | 6.25 | 6.04 | 6.83 | 5.97 | 4.91 |
| **Germany** | 2824 | 2711 | 2659 | 2626 | 2772 | 2829 | 2939 | 2745 | 2265 | 24370 |
| **%** | 10.13 | 10.16 | 10.67 | 8.15 | 8.46 | 9.33 | 9.68 | 9.54 | 7.13 | 9.17 |
| **Greece** | 2280 | 2278 | 0 | 1905 | 2588 | 0 | 0 | 0 | 0 | 9051 |
| **%** | 8.18 | 8.54 | 0.00 | 5.91 | 7.90 | 0.00 | 0.00 | 0.00 | 0.00 | 3.41 |
| **Ireland** | 1811 | 2014 | 1432 | 1685 | 2396 | 2485 | 2200 | 2486 | 2010 | 18519 |
| **%** | 6.50 | 7.55 | 5.75 | 5.23 | 7.31 | 8.20 | 7.25 | 8.64 | 6.33 | 6.97 |
| **Latvia** | 0 | 0 | 0 | 1759 | 0 | 0 | 0 | 0 | 878 | 2637 |
| **%** | 0.00 | 0.00 | 0.00 | 5.46 | 0.00 | 0.00 | 0.00 | 0.00 | 2.76 | 0.99 |
| **Lithuania** | 0 | 0 | 0 | 0 | 1342 | 1915 | 1997 | 1891 | 1708 | 8853 |
| **%** | 0.00 | 0.00 | 0.00 | 0.00 | 4.10 | 6.32 | 6.58 | 6.57 | 5.38 | 3.33 |
| **Netherlands** | 2301 | 1820 | 1848 | 1731 | 1775 | 1784 | 1843 | 1637 | 1591 | 16330 |
| **%** | 8.25 | 6.82 | 7.42 | 5.37 | 5.42 | 5.88 | 6.07 | 5.69 | 5.01 | 6.15 |
| **Norway** | 1**%**966 | 1699 | 1699 | 1502 | 1488 | 1504 | 1315 | 1368 | 1325 | 13866 |
| **%** | 7.05 | 6.37 | 6.82 | 4.66 | 4.54 | 4.96 | 4.33 | 4.75 | 4.17 | 5.22 |
| **Portugal** | 1359 | 1780 | 1930 | 2215 | 2033 | 2026 | 1186 | 1165 | 960 | 14654 |
| **%** | 4.88 | 6.67 | 7.75 | 6.87 | 6.20 | 6.68 | 3.91 | 4.05 | 3.02 | 5.52 |
| **Slovenia** | 1283 | 941 | 1347 | 1165 | 1161 | 1196 | 1174 | 1237 | 1252 | 10756 |
| **%** | 4.60 | 3.53 | 5.41 | 3.61 | 3.54 | 3.94 | 3.87 | 4.30 | 3.94 | 4.05 |
| **Sweden** | 1936 | 1892 | 1862 | 1782 | 1459 | 1772 | 1709 | 1491 | 1426 | 15329 |
| **%** | 6.95 | 7.09 | 7.47 | 5.53 | 4.45 | 5.84 | 5.63 | 5.18 | 4.49 | 5.77 |
| **Switzerland** | 1921 | 2070 | 1753 | 1744 | 1455 | 1438 | 1483 | 1458 | 1450 | 14772 |
| **%** | 6.89 | 7.76 | 7.03 | 5.41 | 4.44 | 4.74 | 4.88 | 5.07 | 4.57 | 5.56 |
| **United Kingdom** | 1967 | 0 | 0 | 2259 | 2229 | 2087 | 1953 | 1706 | 1985 | 14186 |
| **%** | 7.06 | 0.00 | 0.00 | 7.01 | 6.80 | 6.88 | 6.43 | 5.93 | 6.25 | 5.34 |
| **Total** | 27876 | 26682 | 24919 | 32238 | 32768 | 30319 | 30360 | 28776 | 31760 | 265698 |
| **%** | 100.0 | 100.0 | 100.0 | 100.0 | 100.0 | 100.0 | 100.0 | 100.00 | 100.0 | 100.0 |
|  | | | | | | | | | | |

*Note: First row has frequencies and second row has percentages across ESS rounds within country.*

## Table A3. Number of non-migrant respondents per country in each ESS round

|  |  |  |  |  |  |  |  |  |  |
| --- | --- | --- | --- | --- | --- | --- | --- | --- | --- |
| **ESS round** | | | | | | | | | |
| **Country** | **1** | **2** | **3** | **4** | **5** | **6** | **7** | **8** | **9** |
| **Austria** | 1768 | 1875 | 2016 | 0 | 0 | 0 | 1417 | 1619 | 2008 |
| **Belgium** | 1569 | 1441 | 1481 | 1399 | 1353 | 1402 | 1338 | 1321 | 1291 |
| **Switzerland** | 1436 | 1488 | 1210 | 1170 | 949 | 935 | 899 | 885 | 842 |
| **Czechia** | 1199 | 2676 | 0 | 1827 | 2200 | 1748 | 1899 | 2051 | 2152 |
| **Germany** | 2500 | 2435 | 2484 | 2274 | 2454 | 2378 | 2460 | 2234 | 1810 |
| **Denmark** | 1348 | 1324 | 1317 | 1424 | 1362 | 1444 | 1304 | 0 | 1368 |
| **Estonia** | 0 | 1236 | 859 | 994 | 1223 | 1492 | 1144 | 1331 | 1304 |
| **Finland** | 1886 | 1934 | 1813 | 2106 | 1768 | 2063 | 1945 | 1807 | 1626 |
| **France** | 1163 | 1470 | 1564 | 1671 | 1362 | 1500 | 1420 | 1591 | 1520 |
| **UK** | 1716 | 1561 | 1982 | 1921 | 1944 | 1815 | 1747 | 1504 | 1717 |
| **Greece** | 2076 | 1986 | 0 | 1864 | 2299 | 0 | 0 | 0 | 0 |
| **Croatia** | 0 | 0 | 0 | 1220 | 1336 | 0 | 0 | 0 | 1419 |
| **Ireland** | 1807 | 2038 | 1482 | 1417 | 2067 | 2122 | 1965 | 2165 | 1703 |
| **Lithuania** | 0 | 0 | 0 | 0 | 1471 | 1884 | 1969 | 1913 | 1670 |
| **Latvia** | 0 | 0 | 0 | 1376 | 0 | 0 | 0 | 0 | 611 |
| **The Netherlands** | 2079 | 1583 | 1592 | 1502 | 1562 | 1540 | 1576 | 1412 | 1364 |
| **Norway** | 1841 | 1548 | 1541 | 1347 | 1310 | 1349 | 1195 | 1287 | 1177 |
| **Portugal** | 1389 | 1907 | 2014 | 2175 | 1949 | 1968 | 1123 | 1127 | 886 |
| **Sweden** | 1624 | 1611 | 1532 | 1454 | 1197 | 1420 | 1415 | 1227 | 1158 |
| **Slovenia** | 1276 | 1147 | 1190 | 1043 | 1163 | 1021 | 1004 | 1052 | 1038 |
| **Total** | 26677 | 29260 | 24077 | 28184 | 28969 | 26081 | 25820 | 24526 | 26664 |
|  |  |  |  |  |  |  |  |  |  |

## Table A4. Number of first-generation immigrant respondents per country in each ESS round

| **ESS round** | | | | | | | | | |
| --- | --- | --- | --- | --- | --- | --- | --- | --- | --- |
| **Country** | **1** | **2** | **3** | **4** | **5** | **6** | **7** | **8** | **9** |
| **Austria** | 189 | 163 | 152 | 0 | 0 | 0 | 201 | 196 | 271 |
| **Belgium** | 134 | 138 | 127 | 159 | 173 | 247 | 212 | 241 | 263 |
| **Switzerland** | 324 | 378 | 333 | 418 | 341 | 332 | 383 | 415 | 416 |
| **Czechia** | 54 | 86 | 0 | 33 | 43 | 45 | 42 | 50 | 55 |
| **Germany** | 207 | 232 | 210 | 208 | 273 | 277 | 284 | 278 | 281 |
| **Denmark** | 70 | 58 | 83 | 91 | 90 | 103 | 105 | 0 | 92 |
| **Estonia** | 0 | 354 | 300 | 288 | 262 | 366 | 379 | 276 | 255 |
| **Finland** | 42 | 27 | 39 | 41 | 52 | 74 | 82 | 63 | 66 |
| **France** | 132 | 125 | 173 | 146 | 141 | 192 | 202 | 189 | 219 |
| **UK** | 168 | 152 | 204 | 214 | 250 | 251 | 290 | 240 | 278 |
| **Greece** | 193 | 193 | 0 | 99 | 239 | 0 | 0 | 0 | 0 |
| **Croatia** | 0 | 0 | 0 | 112 | 156 | 0 | 0 | 0 | 162 |
| **Ireland** | 93 | 81 | 178 | 239 | 340 | 317 | 260 | 384 | 325 |
| **Lithuania** | 0 | 0 | 0 | 0 | 61 | 57 | 64 | 46 | 42 |
| **Latvia** | 0 | 0 | 0 | 254 | 0 | 0 | 0 | 0 | 95 |
| **The Netherlands** | 134 | 145 | 165 | 151 | 131 | 138 | 168 | 123 | 161 |
| **Norway** | 110 | 116 | 115 | 119 | 131 | 190 | 156 | 156 | 137 |
| **Portugal** | 45 | 67 | 90 | 106 | 113 | 98 | 58 | 57 | 97 |
| **Sweden** | 205 | 175 | 211 | 199 | 165 | 227 | 220 | 172 | 217 |
| **Slovenia** | 15 | 81 | 88 | 92 | 109 | 97 | 88 | 112 | 139 |
| **Total** | 2115 | 2571 | 2468 | 2969 | 3070 | 3011 | 3194 | 2998 | 3571 |

*Note:* Highlighted cluster is later dropped from the analysis due to the small sample size. We ran the analyses also including these respondents and the results were comparable.

## Table A5. Number of second-generation immigrant respondents per country in each ESS round

| **ESS round** | | | | | | | | | |
| --- | --- | --- | --- | --- | --- | --- | --- | --- | --- |
| **Country** | 1 | 2 | 3 | 4 | 5 | 6 | 7 | 8 | 9 |
| **Austria** | 247 | 193 | 206 | 0 | 0 | 0 | 164 | 179 | 208 |
| **Belgium** | 162 | 172 | 159 | 183 | 160 | 196 | 198 | 180 | 192 |
| **Switzerland** | 252 | 251 | 249 | 217 | 206 | 217 | 237 | 208 | 268 |
| **Czechia** | 96 | 170 | 0 | 136 | 122 | 133 | 169 | 157 | 165 |
| **Germany** | 191 | 170 | 186 | 230 | 269 | 262 | 265 | 300 | 226 |
| **Denmark** | 68 | 83 | 78 | 75 | 107 | 85 | 75 | 0 | 90 |
| **Estonia** | 0 | 328 | 300 | 286 | 269 | 448 | 457 | 365 | 324 |
| **Finland** | 43 | 43 | 22 | 30 | 40 | 36 | 38 | 35 | 36 |
| **France** | 181 | 188 | 209 | 228 | 202 | 242 | 259 | 248 | 226 |
| **UK** | 132 | 150 | 160 | 176 | 189 | 193 | 187 | 176 | 177 |
| **Greece** | 219 | 174 | 0 | 79 | 143 | 0 | 0 | 0 | 0 |
| **Croatia** | 0 | 0 | 0 | 126 | 127 | 0 | 0 | 0 | 201 |
| **Ireland** | 69 | 79 | 70 | 59 | 94 | 113 | 95 | 134 | 113 |
| **Lithuania** | 0 | 0 | 0 | 0 | 97 | 132 | 166 | 127 | 90 |
| **Latvia** | 0 | 0 | 0 | 304 | 0 | 0 | 0 | 0 | 195 |
| **The Netherlands** | 125 | 131 | 118 | 105 | 124 | 137 | 154 | 130 | 129 |
| **Norway** | 61 | 84 | 82 | 71 | 63 | 71 | 70 | 86 | 78 |
| **Portugal** | 25 | 24 | 58 | 48 | 54 | 50 | 44 | 42 | 34 |
| **Sweden** | 155 | 148 | 171 | 157 | 123 | 191 | 133 | 131 | 145 |
| **Slovenia** | 40 | 164 | 164 | 127 | 109 | 116 | 113 | 124 | 118 |
| **Total** | 2066 | 2552 | 2232 | 2637 | 2498 | 2622 | 2824 | 2622 | 3015 |

*Note:* Highlighted clusters are later dropped from the analysis due to the small sample size. We ran the analyses also including these respondents and the results were comparable.

## Table A6. Dependent variable coding

|  |  | | **Measure** | | **Original Scale** | | **Note** | |
| --- | --- | --- | --- | --- | --- | --- | --- | --- |
| **a** | | “To what extent do you think [country] should allow people of the same race or ethnic group as most [country]’s people to come and live here?” | | 4-point scale (1 - allow many to come and live here; 2 - allow some; 3 - allow few; 4 - allow none) | | Reverse coded, higher numbers more positive | |  |
| **b** | | “How about people of a different race or ethnic group from most [country] people?” | | 4-point scale | | Reverse coded | |  |
| **c** | | “And how about people from the poorer countries outside Europe?” | | 4-point scale | | Reverse coded | |  |
| **d** | | “Would you say that [country]’s cultural life is generally undermined or enriched by people coming to live here from other countries?” | | 11-point scale (0 – cultural life undermined … 10 – cultural life enriched) | | Recoded to 4-point scale, higher numbers more positive | |  |
| **e** | | “Would you say it is generally bad or good for [country]’s economy that people come to live here from other countries?” | | 11-point scale (0 – bad for the economy … 10 – good for the economy) | | Recoded to 4-point scale | |  |
| **f** | | “Is [country] made a worse or a better place to live by people coming to live here from other countries?” | | 11-point scale (0 –worse place to live… 10 – better place to live) | | Recoded to 4-point scale | |  |

## Table A7. Independent variables coding

| **Variable** | **Coding** | **Note** |
| --- | --- | --- |
| **Age** | Continuous measure of the age of respondent | Minimal value = , Maximal value = |
| **Age squared** | Square term of the continuous measure of the age of respondent |  |
| **Gender** | 0=Female, 1=Male |  |
| **Employment** | 0 = Unemployed, 1 = Employed |  |
| **Education** | 1 = Lower secondary or lower, 2 = Upper secondary, 3 = Vocational, 4 = Tertiary |  |
| **Income** | 1=Respondent is living comfortably, 2 = Respondent is coping on present income, 3 = Respondent finds it difficult to live on income, 4 = Respondents finds it very difficult to live on present income |  |
| **Meeting socially** | 0 = Never, 1 = Monthly, 2 = Weekly, 3 = Daily |  |
| **Feeling of being discriminated** | 0 = Not feeling discriminated against, 1 = Feeling discriminated against | Index consisting of 2 questions 1) Would you describe yourself as being a member of a group that is discriminated against in this country and 2) On what grounds is your group discriminated against. Respondents who answer yes to 1) and choose race, nationality, ethnicity, language, or religion are coded as 1. |
| **Ethnic minority belonging** | 0 = Not belonging to an ethnic minority group in the country 1 = Belonging to such a group |  |
| **Citizenship** | 0 = No citizenship in the country of interview, 1 = Citizenship |  |
| **Religiosity** | Continuous measure of the religiosity of respondent, 0 = Not at all religious, 10 = Very religious |  |
| **Religion** | 1 = Roman Catholic, 2 = Protestant, 3 = Eastern Orthodox, 4 = Other Christian denomination, 5 = Other non-Christian denominations & Jewish, 6 = Islam, 7 = Eastern religions | Jewish recoded as a part of Other non-Christian denominations due to the small sample size. |
| **Language most often spoken at home** | 0 = Speaking minority language, 1 = speaking majority language | Index of 2 questions: Two most often spoken language at home. If one of them is a (co)official in the country coded as 0. |
| **Region of origin** | 0 = Non-migrants, 1 = Old EU, 2 = New EU, 3 = Global North and Australia, 4 = Indian Subcontinent, 5 = South Asia, 7 = Africa, 8 = Middle East, 9 = South America, 11 = Balkan, 12 = Central Asia | East Asia (6) and Oceania (10) was recoded as South Asia due to the small sample size. |
| **Time since arrival to the destination** | 0 = Non-migrant, 1 = Less than a year, 2 = 1-5 years, 3 = 6-10 years, 4 = 11-20 years, 5= 21+ years | The categorisation is done based on the categories from ESS rounds 1 - 4. |

## Table A8. Descriptive statistics (mean, standard deviation, minimum and maximum) of variables

|  | | | | |
| --- | --- | --- | --- | --- |
|  |  | **N** | **%** |  |
| **Sex** | Female | 154074 | 53,27 |  |
|  | Male | 134963 | 46,66 |  |
|  | Missing | 219 | 0,08 |  |
| **Age** | Mean/S.D. | 48,71/0.03 |  |  |
|  | Missing | 1227 | 0,42 |  |
|  | Min | 16 |  |  |
|  | Max | 100 |  |  |
| **Employed** | Unemployed | 136627 | 47,23 |  |
|  | Employed | 152592 | 52,75 |  |
|  | Missing | 37 | 0,01 |  |
| **Education** | Less than lower secondary and secondary | 85393 | 29,52 |  |
|  | Upper Secondary | 108536 | 37,52 |  |
|  | Vocational | 12926 | 4,47 |  |
|  | Tertiary | 80864 | 27,96 |  |
|  | Missing | 1537 | 0,53 |  |
| **Income** | Living comfortably on income | 96594 | 33,39 |  |
|  | Coping on income | 128329 | 44,37 |  |
|  | Difficult on income | 43031 | 14,88 |  |
|  | Very difficult on income | 15010 | 5,19 |  |
|  | Missing | 6292 | 2,18 |  |
| **Meeting socially** | Never | 4183 | 1,45 |  |
|  | Monthly | 45503 | 15,73 |  |
|  | Weekly | 108313 | 37,45 |  |
|  | Daily | 130511 | 45,12 |  |
|  | Missing | 746 | 0,26 |  |
| **Discriminated group** | Not discriminated | 280046 | 96,82 |  |
|  | discriminated | 9173 | 3,17 |  |
|  | Missing | 37 | 0,01 |  |
| **Ethnic minority** | Not minority | 271293 | 93,79 |  |
|  | Belonging to Ethnic minority | 14607 | 5,05 |  |
|  | Missing | 3356 | 1,16 |  |
| **Citizenship** | No citizenship | 14665 | 5,07 |  |
|  | citizenship | 274472 | 94,89 |  |
|  | Missing | 119 | 0,04 |  |
| **Religiosity** | Mean/S.D. | 4,48/0.001 |  |  |
|  | Min | 0 |  |  |
|  | Max | 10 |  |  |
|  | Missing | 2214 | 0,77 |  |
| **Religion** | Non-religious | 124081 | 42,90 |  |
|  | Roman Catholic | 82688 | 28,59 |  |
|  | Protestant | 47972 | 16,58 |  |
|  | Eastern Orthodox | 13326 | 4,61 |  |
|  | Other Christian denomination | 3445 | 1,19 |  |
|  | Other non-Christian denominations | 1128 | 0,39 |  |
|  | Islam | 4746 | 1,64 |  |
|  | Eastern religions | 1143 | 0,40 |  |
|  | Missing | 10727 | 3,71 |  |
| **Minority language** | Speaking majority language | 271703 | 93,93 |  |
|  | Speaking minority language | 15872 | 5,49 |  |
|  | Missing | 1681 | 0,58 |  |
| **Region of origin** | Non-migrant | 263266 | 91,01 |  |
|  | Old EU | 6628 | 2,29 |  |
|  | New EU | 4697 | 1,62 |  |
|  | Global North and Australia | 3114 | 1,08 |  |
|  | Indian Subcontinent | 813 | 0,28 |  |
|  | South Asia | 888 | 0,31 |  |
|  | Africa | 2793 | 0,97 |  |
|  | Middle East | 1726 | 0,60 |  |
|  | South America | 1316 | 0,45 |  |
|  | Balkan | 2356 | 0,81 |  |
|  | Central Asia | 564 | 0,19 |  |
|  | Missing | 1095 | 0,38 |  |
| **Years since arrival** | Non-migrant | 263266 | 91,01 |  |
|  | Less than a year | 310 | 0,11 |  |
|  | 1-5 years | 3382 | 1,17 |  |
|  | 6-10 years | 3315 | 1,15 |  |
|  | 11-20 years | 5594 | 1,93 |  |
|  | 21+ years | 12945 | 4,48 |  |
|  | Missing | 444 | 0,15 |  |
| **Total** |  | 289256 | 100,00 |  |

## Table A9a. Results for a three-level multilevel hierarchical model for attitudes regarding open borders in the full sample

|  | **Allow different race** | | | | | | **Allow same race** | | | | | | **Allow poor countries** | | | | | |
| --- | --- | --- | --- | --- | --- | --- | --- | --- | --- | --- | --- | --- | --- | --- | --- | --- | --- | --- |
|  | **Model 0** | | **Model 1** | | **Model 2** | | **Model 0** | | **Model 1** | | **Model 2** | | **Model 0** | | **Model 1** | | **Model 2** | |
|  | **Coeff.** | **S.E.** | **Coeff.** | **S.E.** | **Coeff.** | **S.E.** | **Coeff.** | **S.E.** | **Coeff.** | **S.E.** | **Coeff.** | **S.E.** | **Coeff.** | **S.E.** | **Coeff.** | **S.E.** | **Coeff.** | **S.E.** |
| Intercept | 2.531*** | (0.059) | 2.643*** | (0.060) | 2.625*** | (0.061) | 2.827*** | (0.050) | 2.727*** | (0.051) | 2.714*** | (0.052) | 2.440*** | (0.062) | 2.711*** | (0.064) | 2.695*** | (0.066) |
| ***Individual-level*** | | | | | | | | | | | | | | | | | | |
| First generation |  |  | 0.127*** | (0.008) | 0.162*** | (0.034) |  |  | 0.123*** | (0.007) | 0.151*** | (0.029) |  |  | 0.095*** | (0.007) | 0.126*** | (0.031) |
| Second generation |  |  | 0.098*** | (0.006) | 0.108*** | (0.013) |  |  | 0.094*** | (0.005) | 0.098*** | (0.011) |  |  | 0.093*** | (0.006) | 0.099*** | (0.012) |
| Age |  |  | -0.008*** | (0.001) | -0.007*** | (0.0005) |  |  | -0.006*** | (0.001) | -0.005*** | (0.004) |  |  | -0.009*** | (0.005) | -0.009*** | (0.001) |
| Male |  |  | -0.018*** | (0.003) | -0.018*** | (0.003) |  |  | 0.002 | (0.003) | 0.001 | (0.003) |  |  | -0.036*** | (0.003) | -0.036*** | (0.003) |
| Upper Secondary |  |  | 0.099*** | (0.004) | 0.098*** | (0.004) |  |  | 0.106*** | (0.004) | 0.104*** | (0.004) |  |  | 0.066*** | (0.004) | 0.064*** | (0.004) |
| Non-university tertiary |  |  | 0.179*** | (0.008) | 0.180*** | (0.008) |  |  | 0.175*** | (0.007) | 0.175*** | (0.007) |  |  | 0.133*** | (0.008) | 0.132*** | (0.008) |
| University degree |  |  | 0.377*** | (0.004) | 0.377*** | (0.004) |  |  | 0.347*** | (0.004) | 0.346*** | (0.004) |  |  | 0.320*** | (0.004) | 0.319*** | (0.004) |
| Employed |  |  | -0.015*** | (0.003) | -0.016*** | (0.004) |  |  | -0.026*** | (0.003) | -0.027*** | (0.003) |  |  | -0.014*** | (0.004) | -0.015*** | (0.004) |
| Coping on income |  |  | -0.092*** | (0.004) | -0.090*** | (0.003) |  |  | -0.098*** | (0.003) | -0.096*** | (0.003) |  |  | -0.080*** | (0.003) | -0.079*** | (0.003) |
| Difficult on income |  |  | -0.186*** | (0.005) | -0.184*** | (0.005) |  |  | -0.189*** | (0.005) | -0.187*** | (0.005) |  |  | -0.163*** | (0.005) | -0.161*** | (0.005) |
| Very difficult on income |  |  | -0.278*** | (0.008) | -0.278*** | (0.008) |  |  | -0.294*** | (0.007) | -0.294*** | (0.007) |  |  | -0.244*** | (0.008) | -0.244*** | (0.008) |
| Socializing Monthly |  |  | 0.125*** | (0.013) | 0.125*** | (0.013) |  |  | 0.175*** | (0.013) | 0.175*** | (0.013) |  |  | 0.128*** | (0.014) | 0.127*** | (0.014) |
| Socializing Weekly |  |  | 0.185*** | (0.013 ) | 0.185*** | (0.013) |  |  | 0.245*** | (0.013) | 0.244*** | (0.013) |  |  | 0.179*** | (0.014) | 0.179*** | (0.014) |
| Socializing Daily |  |  | 0.238*** | (0.013) | 0.238*** | (0.013) |  |  | 0.290*** | (0.013) | 0.289*** | (0.013) |  |  | 0.230*** | (0.014) | 0.229*** | (0.014) |
| Discriminated group |  |  | 0.033*** | (0.009) | 0.027** | (0.009) |  |  | 0.037*** | (0.009) | 0.030** | (0.009) |  |  | 0.045*** | (0.010) | 0.039*** | (0.010) |
| Ethnic minority |  |  | 0.066*** | (0.008) | 0.077*** | (0.008) |  |  | 0.033*** | (0.008) | 0.041*** | (0.008) |  |  | 0.058*** | (0.008) | 0.062*** | (0.008) |
| Citizenship |  |  | -0.008*** | (0.001) | -0.007*** | (0.001) |  |  | -0.007*** | (0.001) | -0.006*** | (0.001) |  |  | -0.008*** | (0.001) | -0.007*** | (0.001) |
| Minority language |  |  | -0.023* | (0.009) | -0.014 | (0.009) |  |  | -0.004 | (0.008) | 0.002 | (0.009) |  |  | -0.034*** | (0.009) | -0.034 *** | (0.009) |
| religiosity |  |  | 0.006*** | (0.001) | 0.006*** | (0.001) |  |  | 0.006*** | (0.001) | 0.007*** | (0.001) |  |  | 0.010*** | (0.001) | 0.010*** | (0.001) |
| Roman Catholic |  |  | -0.101*** | (0.004) | -0.095*** | (0.004) |  |  | -0.064*** | (0.004) | -0.057*** | (0.004) |  |  | -0.094*** | (0.005) | -0.090*** | (0.005) |
| Protestant |  |  | -0.058*** | (0.005) | -0.062*** | (0.005) |  |  | -0.012* | (0.005) | -0.017*** | (0.005) |  |  | -0.064*** | (0.005) | -0.068*** | (0.005) |
| Eastern Orthodox |  |  | -0.131*** | (0.012) | -0.107*** | (0.012) |  |  | -0.074*** | (0.012) | -0.053*** | (0.012) |  |  | -0.149*** | (0.012) | -0.128*** | (0.013) |
| Other Christian denomination |  |  | 0.091*** | (0.014) | 0.085*** | (0.014) |  |  | 0.093*** | (0.014) | 0.0867*** | (0.014) |  |  | 0.096*** | (0.015) | 0.0916*** | (0.015) |
| Other non-Christian denominations |  |  | 0.062* | (0.024) | 0.064** | (0.024) |  |  | 0.040 | (0.024) | 0.040 | (0.024) |  |  | 0.087*** | (0.025) | 0.090*** | (0.025) |
| Islam |  |  | 0.101*** | (0.013) | 0.114*** | (0.014) |  |  | -0.0178 | (0.013) | -0.003 | (0.013) |  |  | 0.113*** | (0.014) | 0.128*** | (0.014) |
| Eastern religions |  |  | 0.123*** | (0.025) | 0.122*** | (0.025) |  |  | 0.0225 | (0.025) | 0.026 | (0.025) |  |  | 0.121*** | (0.026) | 0.117*** | (0.026) |
| ***Random effect estimates*** | | | | | | | | | | | | | | | | | | |
| Country | 0.067 | (0.022) | 0.0436 | (0.014) | 0.047 | (0.016) | 0.047 | (0.016) | 0.027 | (0.009) | 0.029 | (0.009) | 0.074 | (0.024) | 0.054 | (0.017) | 0.058 | (0.019) |
| Period (in country) | 0.020 | (0.002) | 0.0146 | (0.001) | 0.014 | (0.002) | 0.020 | (0.002) | 0.012 | (0.001) | 0.013 | (0.002) | 0.020 | (0.002) | 0.014 | (0.001) | 0.014 | (0.001) |
| Individual | 0.667 | (0.002) | 0.6106 | (0.001) | 0.609 | (0.002) | 0.625 | (0.002) | 0.586 | (0.001) | 0.585 | (0.002) | 0.698 | (0.001) | 0.647 | (0.001) | 0.646 | (0.001) |
| First generation |  |  |  |  | 0.022 | (0.007) |  |  |  |  | 0.016 | (0.005) |  |  |  |  | 0.017 | (0.005) |
| Second generation |  |  |  |  | 0.002 | (0.001) |  |  |  |  | 0.002 | (0.001) |  |  |  |  | 0.002 | (0.001) |

## Table A9b. Results for a regression model including fixed effect country and country/period variable for attitudes regarding open borders in the full sample.

|  | **Allow same race** | | | | | | **Allow different race** | | | | | **Allow poor countries** | | | | |
| --- | --- | --- | --- | --- | --- | --- | --- | --- | --- | --- | --- | --- | --- | --- | --- | --- |
|  | **Model 0** | | | | **Model 1** | | **Model 0** | | | **Model 1** | | **Model 0** | | | **Model 1** | |
|  | **Coef** | | **S.E.** | | **Coef** | **S.E.** | **Coef** | | **S.E.** | **Coef** | **S.E.** | **Coef** | **S.E.** | | **Coef** | **S.E.** |
| Constant | 2.480*** | | (0.018) | | 2.499*** | (0.026) | 2.326*** | | (0.019) | 2.727*** | (0.051) | 2.330*** | (0.019) | | 2.594*** | (0.028) |
| ***Individual-level*** |  | |  |  | |  |  | |  |  |  |  |  | |  |  |
| First generation |  | |  | 0.123*** | | (0.008) | |  |  | 0.126*** | (0.008) |  |  | | 0.0950*** | (0.008) |
| Second generation |  | |  | 0.0944*** | | (0.006) |  | |  | 0.0988*** | (0.006) |  | |  | 0.0937*** | (0.006) |
| Age |  | |  | -0.00602*** | | (0.001) |  | |  | -0.00777*** | (0.000) |  |  | | -0.00954*** | (0.001) |
| Male |  | |  | 0.00153 | | (0.003) |  | |  | -0.0177*** | (0.003) |  |  | | -0.036*** | (0.003) |
| Upper Secondary |  | |  | 0.105*** | | (0.004) |  | |  | 0.0995*** | (0.004) |  |  | | 0.0660*** | (0.004) |
| Non-university tertiary |  | |  | 0.175*** | | (0.008) |  | |  | 0.180*** | (0.008) |  |  | | 0.133*** | (0.009) |
| University degree |  | |  | 0.346*** | | (0.004) |  | |  | 0.377*** | (0.005) |  |  | | 0.320*** | (0.005) |
| Employed |  | |  | -0.0268*** | | (0.004) |  | |  | -0.0151*** | (0.004) |  |  | | -0.0146*** | (0.004) |
| Coping on income |  | |  | -0.0981*** | | (0.004) |  | |  | -0.0920*** | (0.004) |  |  | | -0.0806*** | (0.004) |
| Difficult on income |  | |  | -0.188*** | | (0.005) |  | |  | -0.186*** | (0.005) |  |  | | -0.162*** | (0.005) |
| Very difficult on income |  | |  | -0.294*** | | (0.008) |  | |  | -0.278*** | (0.008) |  |  | | -0.244*** | (0.008) |
| Socializing Monthly |  | |  | 0.175*** | | (0.013) |  | |  | 0.125*** | (0.014) |  |  | | 0.128*** | (0.014) |
| Socializing Weekly |  | |  | 0.245*** | | (0.013) |  | |  | 0.185*** | (0.014) |  |  | | 0.180*** | (0.014) |
| Socializing Daily |  | |  | 0.290*** | | (0.013) |  | |  | 0.238*** | (0.014) |  |  | | 0.230*** | (0.014) |
| Discriminated group |  | |  | 0.0371*** | | (0.010) |  | |  | 0.0333*** | (0.010) |  |  | | 0.0456*** | (0.010) |
| Ethnic minority |  | |  | 0.0333*** | | (0.008) |  | |  | 0.0658*** | (0.009) |  |  | | 0.0586*** | (0.009) |
| Citizenship |  | |  | -0.00726*** | | (0.001) |  | |  | -0.00786*** | (0.001) |  |  | | -0.00826*** | (0.001) |
| Minority language |  | |  | -0.00376 | | (0.009) |  | |  | -0.0229* | (0.009) |  |  | | -0.0341*** | (0.010) |
| religiosity |  | |  | 0.00671*** | | (0.001) |  | |  | 0.00626*** | (0.001) |  |  | | 0.0103*** | (0.001) |
| ***Fixed effect*** |  |  | |  | |  |  | |  |  |  |  |  | |  |  |
| Country | Yes |  | | Yes | |  | Yes | |  | Yes |  | Yes |  | | Yes |  |
| Period (in country) | Yes |  | | Yes | |  | Yes | |  | Yes |  | Yes |  | | Yes |  |

## Table A10a. Results for a three-level multilevel hierarchical model for attitudes regarding the effects of immigration in the full sample

|  | **Immigration‘s effect on country** | | | | | | | **Immigration‘s effect on economy** | | | | | | **Immigration‘s effect on culture** | | | | | |
| --- | --- | --- | --- | --- | --- | --- | --- | --- | --- | --- | --- | --- | --- | --- | --- | --- | --- | --- | --- |
|  | **Model 0** | | | **Model 1** | | **Model 2** | | **Model 0** | | **Model 1** | | **Model 2** | | **Model 0** | | **Model 1** | | **Model 2** | |
|  | **Coeff.** | | **S.E.** | **Coeff.** | **S.E.** | **Coeff.** | **S.E.** | **Coeff.** | **S.E.** | **Coeff.** | **S.E.** | **Coeff.** | **S.E.** | **Coeff.** | **S.E.** | **Coeff.** | **S.E.** | **Coeff.** | **S.E.** |
| Intercept | 2.467*** | | (0.044) | 2.465*** | (0.042) | 2.452*** | (0.043) | 2.487*** | (0.037) | 2.411*** | (0.038) | 2.390*** | (0.040) | 2.647*** | (0.055) | 2.631*** | (0.052) | 2.615*** | (0.055) |
| ***Individual-level*** | | | | | | | | | | | | | | | | | | | |
| First generation |  | |  | 0.183*** | (0.006) | 0.192*** | (0.027) |  |  | 0.169*** | (0.006) | 0.182*** | (0.034) |  |  | 0.158*** | (0.006) | 0.176*** | (0.034) |
| Second generation |  | |  | 0.076*** | (0.004) | 0.073*** | (0.010) |  |  | 0.072*** | (0.005) | 0.075*** | (0.013) |  |  | 0.099*** | (0.005) | 0.099*** | (0.012) |
| Age |  | |  | -0.004*** | (0.001) | -0.004*** | (0.001) |  |  | -0.003*** | (0.001) | -0.003*** | (0.001) |  |  | -0.0007 | (0.001) | -0.0006 | (0.001) |
| Male |  | |  | -0.004 | (0.002) | -0.004 | (0.002) |  |  | 0.066*** | (0.002) | 0.066*** | (0.002) |  |  | -0.039*** | (0.002) | -0.039*** | (0.002) |
| Upper Secondary |  | |  | 0.071*** | (0.003) | 0.069*** | (0.003) |  |  | 0.091*** | (0.003) | 0.089*** | (0.003) |  |  | 0.099*** | (0.003) | 0.098*** | (0.003) |
| Non-university tertiary |  | |  | 0.138*** | (0.006) | 0.139*** | (0.006) |  |  | 0.156*** | (0.006) | 0.157*** | (0.006) |  |  | 0.175*** | (0.006) | 0.176*** | (0.006) |
| University degree |  | |  | 0.280*** | (0.004) | 0.279*** | (0.004) |  |  | 0.350*** | (0.004) | 0.348*** | (0.004) |  |  | 0.361*** | (0.004) | 0.360*** | (0.003) |
| Employed |  | |  | -0.003 | (0.003) | -0.003 | (0.003) |  |  | -0.009** | (0.003) | -0.010** | (0.003) |  |  | -0.003 | (0.003) | -0.004 | (0.003) |
| Coping on income |  | |  | -0.089*** | (0.003) | -0.088*** | (0.003) |  |  | -0.106*** | (0.003) | -0.104*** | (0.003) |  |  | -0.089*** | (0.003) | -0.088*** | (0.003) |
| Difficult on income |  | |  | -0.180*** | (0.004) | -0.178*** | (0.004) |  |  | -0.204*** | (0.004) | -0.202*** | (0.004) |  |  | -0.162*** | (0.004) | -0.161*** | (0.004) |
| Very difficult on income |  | |  | -0.274*** | (0.006) | -0.273*** | (0.006) |  |  | -0.297*** | (0.006) | -0.296*** | (0.006) |  |  | -0.231*** | (0.006) | -0.231*** | (0.006) |
| Socializing Monthly |  | |  | 0.103*** | (0.011) | 0.102*** | (0.011) |  |  | 0.123*** | (0.012) | 0.122*** | (0.012) |  |  | 0.120*** | (0.012) | 0.119*** | (0.012) |
| Socializing Weekly |  | |  | 0.158*** | (0.010) | 0.157*** | (0.011) |  |  | 0.193*** | (0.011) | 0.192*** | (0.012) |  |  | 0.180*** | (0.012) | 0.178*** | (0.012) |
| Socializing Daily |  |  | | 0.181*** | (0.011) | 0.180*** | (0.011) |  |  | 0.208*** | (0.011) | 0.208*** | (0.012) |  |  | 0.215*** | (0.012) | 0.214*** | (0.012) |
| Discriminated group |  | |  | -0.044*** | (0.007) | -0.049*** | (0.007) |  |  | -0.006 | (0.008) | -0.016 | (0.008) |  |  | -0.024** | (0.008) | -0.033*** | (0.008) |
| Ethnic minority |  | |  | 0.075*** | (0.006) | 0.082*** | (0.006) |  |  | 0.063*** | (0.007) | 0.075*** | (0.007) |  |  | 0.083*** | (0.007) | 0.089*** | (0.007) |
| Citizenship |  | |  | -0.011*** | (0.001) | -0.010*** | (0.001) |  |  | -0.012*** | (0.001) | -0.011*** | (0.001) |  |  | -0.009*** | (0.001) | -0.008*** | (0.001) |
| Minority language |  | |  | 0.003 | (0.007) | 0.014 | (0.007) |  |  | -0.007 | (0.007) | 0.010 | (0.008) |  |  | -0.068*** | (0.007) | -0.059*** | (0.008) |
| religiosity |  | |  | 0.012*** | (0.001) | 0.012*** | (0.001) |  |  | 0.009*** | (0.001) | 0.009*** | (0.001) |  |  | 0.008*** | (0.001) | 0.008*** | (0.001) |
| Roman Catholic |  | |  | -0.075*** | (0.003) | -0.072*** | (0.004) |  |  | -0.056*** | (0.004) | -0.051*** | (0.004) |  |  | -0.101*** | (0.004) | -0.096*** | (0.004) |
| Protestant |  | |  | -0.048*** | (0.004) | -0.051*** | (0.004) |  |  | -0.024*** | (0.004) | -0.028*** | (0.004) |  |  | -0.063*** | (0.004) | -0.068*** | (0.004) |
| Eastern Orthodox |  | |  | -0.094*** | (0.010) | -0.058*** | (0.010) |  |  | -0.127*** | (0.011) | -0.074*** | (0.011) |  |  | -0.109*** | (0.011) | -0.077*** | (0.011) |
| Other Christian denomination |  | |  | 0.006 | (0.011) | 0.002 | (0.012) |  |  | 0.020 | (0.012) | 0.013 | (0.012) |  |  | 0.008 | (0.012) | 0.005 | (0.012) |
| Other non-Christian denominations |  | |  | 0.045* | (0.019) | 0.045* | (0.019) |  |  | 0.058** | (0.021) | 0.055** | (0.021) |  |  | 0.033 | (0.021) | 0.035 | (0.021) |
| Islam |  | |  | 0.201*** | (0.011) | 0.203*** | (0.011) |  |  | 0.145*** | (0.012) | 0.142*** | (0.012) |  |  | 0.205*** | (0.012) | 0.208*** | (0.012) |
| Eastern religions |  | |  | 0.118*** | (0.020) | 0.102*** | (0.020) |  |  | 0.101*** | (0.021) | 0.083*** | (0.021) |  |  | 0.139*** | (0.022) | 0.132*** | (0.022) |
| ***Random effect estimates*** | | | | | | | | | | | | | | | | | | | |
| Country | 0.037 | | (0.012) | 0.022 | (0.007) | 0.025 | (0.008) | 0.026 | (0.009) | 0.014 | (0.005) | 0.016 | (0.005) | 0.061 | (0.019) | 0.042 | (0.013) | 0.047 | (0.015) |
| Period (in country) | 0.008 | | (0.001) | 0.005 | (0.001) | 0.005 | (0.001) | 0.012 | (0.002) | 0.006 | (0.001) | 0.007 | (0.001) | 0.006 | (0.001) | 0.005 | (0.001) | 0.005 | (0.001) |
| Individual | 0.418 | | (0.001) | 0.385 | (0.001) | 0.384 | (0.001) | 0.478 | (0.001) | 0.439 | (0.001) | 0.438 | (0.001) | 0.498 | (0.001) | 0.456 | (0.013) | 0.455 | (0.001) |
| First generation |  | |  |  |  | 0.013 | (0.004) |  |  |  |  | 0.023 | (0.007) |  |  |  |  | 0.022 | (0.007) |
| Second generation |  | |  |  |  | 0.001 | (0.001) |  |  |  |  | 0.003 | (0.001) |  |  |  |  | 0.002 | (0.001) |
| Observations | 257,906 | |  | 257,801 |  | 257,801 |  | 257,277 |  | 257,174 |  | 257,174 |  | 258,330 |  | 258,229 |  | 258,229 |  |

## Table A10b. Results for a regression model including fixed effect country and country/period variable for attitudes regarding effect on immigration in the full sample.

|  | **Immigration's effect on country** | | | | | | **Immigration’s effect on economy** | | | | | **Immigration’s effect on culture** | | | | |
| --- | --- | --- | --- | --- | --- | --- | --- | --- | --- | --- | --- | --- | --- | --- | --- | --- |
|  | **Model 0** | | | | **Model 1** | | **Model 0** | | | **Model 1** | | **Model 0** | | | **Model 1** | |
|  | **Coef** | | **S.E.** | | **Coef** | **S.E.** | **Coef** | | **S.E.** | **Coef** | **S.E.** | **Coef** | **S.E.** | | **Coef** | **S.E.** |
| Constant | 2.421*** | | (0.015) | | 2.496*** | (0.021) | 2.676*** | | (0.016) | 2.652*** | (0.023) | 2.753*** | (0.016) | | 2.720*** | (0.023) |
| ***Individual-level*** |  | |  |  | |  |  | |  |  |  |  |  | |  |  |
| First generation |  | |  | 0.183*** | | (0.006) | |  |  | 0.169*** | (0.007) |  |  | | 0.157*** | (0.007) |
| Second generation |  | |  | 0.076*** | | (0.005) |  | |  | 0.073*** | (0.005) |  | |  | 0.099*** | (0.005) |
| Age |  | |  | -0.004*** | | (0.000) |  | |  | -0.004*** | (0.001) |  |  | | -0.001 | (0.000) |
| Male |  | |  | -0.005 | | (0.003) |  | |  | 0.067*** | (0.003) |  |  | | -0.040*** | (0.003) |
| Upper Secondary |  | |  | 0.0712*** | | (0.003) |  | |  | 0.091*** | (0.004) |  |  | | 0.099*** | (0.004) |
| Non-university tertiary |  | |  | 0.138*** | | (0.006) |  | |  | 0.156*** | (0.007) |  |  | | 0.175*** | (0.007) |
| University degree |  | |  | 0.279*** | | (0.004) |  | |  | 0.350*** | (0.004) |  |  | | 0.361*** | (0.004) |
| Employed |  | |  | -0.003 | | (0.003) |  | |  | -0.010** | (0.003) |  |  | | -0.003 | (0.003) |
| Coping on income |  | |  | -0.090*** | | (0.003) |  | |  | -0.105*** | (0.003) |  |  | | -0.090*** | (0.003) |
| Difficult on income |  | |  | -0.179*** | | (0.004) |  | |  | -0.204*** | (0.004) |  |  | | -0.162*** | (0.005) |
| Very difficult on income |  | |  | -0.273*** | | (0.006) |  | |  | -0.296*** | (0.007) |  |  | | -0.230*** | (0.007) |
| Socializing Monthly |  | |  | 0.103*** | | (0.011) |  | |  | 0.123*** | (0.012) |  |  | | 0.121*** | (0.012) |
| Socializing Weekly |  | |  | 0.158*** | | (0.011) |  | |  | 0.193*** | (0.012) |  |  | | 0.180*** | (0.012) |
| Socializing Daily |  | |  | 0.181*** | | (0.011) |  | |  | 0.208*** | (0.012) |  |  | | 0.216*** | (0.012) |
| Discriminated group |  | |  | -0.044*** | | (0.008) |  | |  | -0.006 | (0.008) |  |  | | -0.025** | (0.008) |
| Ethnic minority |  | |  | 0.075*** | | (0.00682) |  | |  | 0.063*** | (0.007) |  |  | | 0.084*** | (0.007) |
| Citizenship |  | |  | -0.011*** | | (0.001) |  | |  | -0.012*** | (0.001) |  |  | | -0.009*** | (0.001) |
| Minority language |  | |  | 0.005 | | (0.007) |  | |  | -0.008 | (0.008) |  |  | | -0.068*** | (0.008) |
| Religiosity |  | |  | 0.012*** | | (0.001) |  | |  | 0.010*** | (0.001) |  |  | | 0.009*** | (0.001) |
| Roman Catholic |  | |  | -0.075*** | | (0.004) |  | |  | -0.056*** | (0.004) |  |  | | -0.101*** | (0.004) |
| Protestant |  | |  | -0.048*** | | (0.004) |  | |  | -0.025*** | (0.004) |  |  | | -0.064*** | (0.004) |
| Eastern Orthodox |  | |  | -0.094*** | | (0.010) |  | |  | -0.126*** | (0.012) |  |  | | -0.108*** | (0.011) |
| Other Christian denomination |  | |  | 0.007 | | (0.012) |  | |  | 0.020 | (0.013) |  |  | | 0.008 | (0.013) |
| Other non-Christian denominations |  | |  | 0.045* | | (0.020) |  | |  | 0.057** | (0.021) |  |  | | 0.034 | (0.021) |
| Islam |  | |  | 0.201*** | | (0.011) |  | |  | 0.145*** | (0.012) |  |  | | 0.206*** | (0.012) |
| Eastern religions |  | |  | 0.118*** | | (0.020) |  | |  | 0.101*** | (0.022) |  |  | | 0.139*** | (0.022) |
| ***Fixed effect*** |  |  | |  | |  |  | |  |  |  |  |  | |  |  |
| Country | Yes |  | | Yes | |  | Yes | |  | Yes |  | Yes |  | | Yes |  |
| Period (in country) | Yes |  | | Yes | |  | Yes | |  | Yes |  | Yes |  | | Yes |  |

## Table A10c. Results for a three-level multilevel hierarchical model for attitudes regarding the effects of immigration and the attitudes regarding open borders with no controls.

|  | **Allow different race** | | **Allow same race** | | **Allow poor countries** | | **Immigrants' effect on country** | | **Immigrants' effect on economy** | | **Immigrants' effect on culture** | |
| --- | --- | --- | --- | --- | --- | --- | --- | --- | --- | --- | --- | --- |
|  | **Coeff.** | **S.E.** | **Coeff.** | **S.E.** | **Coeff.** | **S.E.** | **Coeff.** | **S.E.** | **Coeff.** | **S.E.** | **Coeff.** | **S.E.** |
| Intercept | 2.501 | (42.10)** | 2.803 | (56.47)** | 2.412 | (38.65)** | 2.434 | (55.05)** | 2.457 | (66.15)** | 2.615 | (47.04)** |
| ***Individual level*** |  |  |  |  |  |  |  |  |  |  |  |  |
| First generation | 0.206 | (34.73)** | 0.166 | (28.91)** | 0.182 | (29.96)** | 0.282 | (60.02)** | 0.252 | (50.21)** | 0.246 | (48.20)** |
| Second generation | 0.157 | (26.15)** | 0.127 | (21.73)** | 0.157 | (25.44)** | 0.119 | (25.07)** | 0.102 | (20.03)** | 0.142 | (27.44)** |
| ***Random effect estimates*** |  |  |  |  |  |  |  |  |  |  |  |  |
| Period (in country) | -1.349 | (8.11)** | /-1.539 | (9.15)** | -1.298 | (7.81)** | -1.639 | (9.99)** | -1.833 | (10.70)** | -1.401 | (8.70)** |
| Country | -1.965 | (30.64)** | /-1.956 | (30.58)** | -1.949 | (30.35)** | -2.390 | (36.89)** | -2.191 | (34.09)** | -2.528 | (38.42)** |
| Individual | -0.206 | (148.26)** | /-0.237 | (170.56)** | /-0.182 | (131.26)** | -0.443 | (317.80)** | -0.373 | (267.74)** | -0.353 | (253.92)** |

## Table A11. Multilevel hierarchical regression coefficients for the dependent variable “allow immigrants of different race” for the three separate samples

|  |  | **Non-migrants** | **Migrants** | **Second generation** |
| --- | --- | --- | --- | --- |
| **Allow different** | **Age** | -0.007 | -0.009 | -0.009 |
|  |  | (0.001)** | (0.002)** | (0.002)** |
|  | **Age squared** | 0.000 | 0.000 | 0.000 |
|  |  | (0.000)** | (0.000) | (0.000) |
|  | **Male** | -0.025 | 0.021 | 0.015 |
|  |  | (0.003)** | (0.011)+ | (0.011) |
|  | **In paid work** | -0.019 | 0.034 | -0.049 |
|  |  | (0.004)** | (0.013)** | (0.014)** |
|  | **Secondary education** | 0.111 | 0.012 | 0.072 |
|  | ***(Ref Elementary)*** | (0.005)** | (0.014) | (0.015)** |
|  | **Vocational education** | 0.200 | 0.038 | 0.131 |
|  |  | (0.009)** | (0.026) | (0.027)** |
|  | **Tertiary education** | 0.398 | 0.207 | 0.352 |
|  |  | (0.005)** | (0.015)** | (0.017)** |
|  | **Coping on income** | -0.092 | -0.072 | -0.096 |
|  | ***(Ref Satisfied)*** | (0.004)** | (0.013)** | (0.013)** |
|  | **Difficult on income** | -0.193 | -0.113 | -0.196 |
|  |  | (0.006)** | (0.017)** | (0.018)** |
|  | **Very difficult on income** | -0.303 | -0.120 | -0.260 |
|  |  | (0.009)** | (0.023)** | (0.027)** |
|  | **Monthly socialising** | 0.126 | 0.044 | 0.191 |
|  | ***(Ref Never)*** | (0.015)** | (0.043) | (0.049)** |
|  | **Weekly socialising** | 0.190 | 0.073 | 0.244 |
|  |  | (0.015)** | (0.043)+ | (0.048)** |
|  | **Daily socialising** | 0.240 | 0.136 | 0.309 |
|  |  | (0.015)** | (0.043)** | (0.048)** |
|  | **Discriminated** | -0.063 | 0.090 | 0.066 |
|  | ***(Ref Non-discrim)*** | (0.015)** | (0.016)** | (0.023)** |
|  | **Minority** | 0.076 | 0.068 | 0.072 |
|  | ***(Ref Non-minor)*** | (0.015)** | (0.013)** | (0.019)** |
|  | **Citizenship** | -0.014 | -0.008 | 0.002 |
|  | ***(Ref Non citizen)*** | (0.005)** | (0.001)** | (0.002) |
|  | **Roman Catholic** | -0.091 | -0.092 | -0.141 |
|  | ***(Ref No-religion)*** | (0.005)** | (0.016)** | (0.017)** |
|  | **Protestant** | -0.063 | -0.051 | -0.065 |
|  |  | (0.005)** | (0.021)* | (0.021)** |
|  | **Eastern Orthodox** | -0.096 | -0.106 | -0.110 |
|  |  | (0.021)** | (0.021)** | (0.029)** |
|  | **Other Christian religions** | 0.113 | -0.037 | 0.143 |
|  |  | (0.017)** | (0.035) | (0.048)** |
|  | **Other Non-Christian religions** | 0.138 | -0.010 | -0.086 |
|  |  | (0.031)** | (0.051) | (0.064) |
|  | **Islam** | 0.249 | 0.062 | 0.012 |
|  |  | (0.056)** | (0.020)** | (0.029) |
|  | **Eastern Religions** | 0.328 | -0.022 | -0.012 |
|  |  | (0.042)** | (0.039) | (0.062) |
|  | **Religiosity** | 0.006 | 0.004 | 0.009 |
|  | ***(Scale)*** | (0.001)** | (0.002)+ | (0.002)** |
|  | **Language spoken at home** | -0.054 | -0.010 | 0.025 |
|  |  | (0.015)** | (0.015) | (0.023) |
|  | **ESS round 2 / 2004** | -0.029 | 0.051 | 0.009 |
|  | ***(Ref ESS 1/2002)*** | (0.047) | (0.042) | (0.051) |
|  | **ESS round 3** **/ 2006** | -0.050 | -0.030 | -0.035 |
|  |  | (0.048) | (0.042) | (0.051) |
|  | **ESS round 4** **/ 2008** | 0.021 | 0.039 | 0.050 |
|  |  | (0.045) | (0.040) | (0.049) |
|  | **ESS round 5** **/ 2010** | 0.021 | 0.032 | 0.090 |
|  |  | (0.045) | (0.039) | (0.048)+ |
|  | **ESS round 6** **/ 2012** | 0.047 | 0.040 | 0.087 |
|  |  | (0.046) | (0.040) | (0.050)+ |
|  | **ESS round 7** **/ 2014** | 0.088 | 0.058 | 0.103 |
|  |  | (0.045)+ | (0.040) | (0.049)* |
|  | **ESS round 8** **/ 2016** | 0.072 | 0.003 | 0.091 |
|  |  | (0.046) | (0.041) | (0.049)+ |
|  | **ESS round 9** **/ 2018** | 0.115 | 0.062 | 0.131 |
|  |  | (0.045)* | (0.039) | (0.048)** |
|  | **Intercept** | 2.673 | 2.975 | 2.658 |
|  |  | (0.078)** | (0.081)** | (0.088)** |
| **Random effects** | **Country** | -1.524 | -1.616 | -1.563 |
|  |  | (0.168)** | (0.167)** | (0.169)** |
|  | **Period** | -2.079 | -2.516 | -2.212 |
|  |  | (0.065)** | (0.113)** | (0.094)** |
|  | **Individual** | -0.250 | -0.258 | -0.238 |
|  |  | (0.002)** | (0.005)** | (0.005)** |
| ***N*** |  | 217,365 | 21,454 | 20,631 |

## Table A12. Multilevel hierarchical regression coefficients for the dependent variable “allow immigrants of same race” for the three separate samples

|  |  | **Non-migrants** | **Migrants** | **Second generation** |
| --- | --- | --- | --- | --- |
| **Allow same** | **Age** | -0.005 | -0.007 | -0.008 |
|  |  | (0.001)** | (0.002)** | (0.002)** |
|  | **Age squared** | 0.000 | 0.000 | 0.000 |
|  |  | (0.000)** | (0.000)+ | (0.000)* |
|  | **Male** | -0.003 | 0.019 | 0.029 |
|  | ***(Ref Female)*** | (0.003) | (0.010)+ | (0.011)** |
|  | **In paid work** | -0.030 | 0.004 | -0.051 |
|  | **(Ref Unpaid)** | (0.004)** | (0.012) | (0.013)** |
|  | **Secondary education** | 0.113 | 0.037 | 0.095 |
|  | ***(Ref Elementary)*** | (0.005)** | (0.014)** | (0.015)** |
|  | **Vocational education** | 0.186 | 0.080 | 0.173 |
|  |  | (0.009)** | (0.024)** | (0.026)** |
|  | **Tertiary education** | 0.362 | 0.203 | 0.340 |
|  |  | (0.005)** | (0.014)** | (0.017)** |
|  | **Coping on income** | -0.098 | -0.083 | -0.096 |
|  | ***(Ref Satisfied)*** | (0.004)** | (0.013)** | (0.013)** |
|  | **Difficult on income** | -0.194 | -0.128 | -0.192 |
|  |  | (0.006)** | (0.016)** | (0.018)** |
|  | **Very difficult on income** | -0.318 | -0.160 | -0.257 |
|  |  | (0.009)** | (0.022)** | (0.026)** |
|  | **Monthly socialising** | 0.175 | 0.103 | 0.252 |
|  | ***(Ref Never)*** | (0.015)** | (0.041)* | (0.047)** |
|  | **Weekly socialising** | 0.251 | 0.122 | 0.309 |
|  |  | (0.015)** | (0.041)** | (0.046)** |
|  | **Daily socialising** | 0.294 | 0.179 | 0.360 |
|  |  | (0.015)** | (0.041)** | (0.046)** |
|  | **Discriminated** | -0.024 | 0.061 | 0.062 |
|  | ***(Ref Non-discrim)*** | (0.015) | (0.015)** | (0.022)** |
|  | **Minority** | 0.054 | 0.030 | 0.041 |
|  | ***(Ref Non-minor)*** | (0.014)** | (0.012)* | (0.019)* |
|  | **Citizenship** | -0.003 | -0.009 | 0.004 |
|  | ***(Ref Non citizen)*** | (0.005) | (0.001)** | (0.002)+ |
|  | **Roman Catholic** | -0.056 | -0.020 | -0.120 |
|  | ***(Ref No-religion)*** | (0.005)** | (0.015) | (0.016)** |
|  | **Protestant** | -0.022 | 0.040 | -0.017 |
|  |  | (0.005)** | (0.020)+ | (0.020) |
|  | **Eastern Orthodox** | -0.005 | -0.062 | -0.051 |
|  |  | (0.021) | (0.020)** | (0.027)+ |
|  | **Other Christian religions** | 0.109 | 0.024 | 0.075 |
|  |  | (0.017)** | (0.034) | (0.047) |
|  | **Other Non-Christian religions** | 0.094 | -0.049 | -0.000 |
|  |  | (0.031)** | (0.049) | (0.062) |
|  | **Islam** | 0.106 | -0.014 | -0.076 |
|  |  | (0.055)+ | (0.019) | (0.028)** |
|  | **Eastern Religions** | 0.231 | -0.076 | -0.116 |
|  |  | (0.041)** | (0.037)* | (0.060)+ |
|  | **Religiosity** | 0.007 | -0.002 | 0.010 |
|  | ***(Scale)*** | (0.001)** | (0.002) | (0.002)** |
|  | **Language spoken at home** | -0.026 | 0.006 | 0.014 |
|  |  | (0.015)+ | (0.014) | (0.022) |
|  | **ESS round 2 / 2004** | 0.017 | 0.043 | 0.039 |
|  | ***(Ref ESS 1/2002)*** | (0.045) | (0.036) | (0.045) |
|  | **ESS round 3** **/ 2006** | 0.015 | 0.047 | 0.049 |
|  |  | (0.045) | (0.036) | (0.046) |
|  | **ESS round 4** **/ 2008** | 0.039 | 0.066 | 0.091 |
|  |  | (0.043) | (0.034)+ | (0.044)* |
|  | **ESS round 5** **/ 2010** | 0.060 | 0.049 | 0.127 |
|  |  | (0.043) | (0.033) | (0.043)** |
|  | **ESS round 6** **/ 2012** | 0.060 | 0.039 | 0.099 |
|  |  | (0.044) | (0.034) | (0.044)* |
|  | **ESS round 7** **/ 2014** | 0.109 | 0.080 | 0.127 |
|  |  | (0.043)* | (0.034)* | (0.044)** |
|  | **ESS round 8** **/ 2016** | 0.135 | 0.072 | 0.121 |
|  |  | (0.044)** | (0.035)* | (0.044)** |
|  | **ESS round 9** **/ 2018** | 0.204 | 0.127 | 0.201 |
|  |  | (0.042)** | (0.033)** | (0.043)** |
|  | **Intercept** | 2.654 | 3.094 | 2.694 |
|  |  | (0.071)** | (0.069)** | (0.080)** |
| **Random effects** | **Country** | -1.744 | -2.038 | -1.790 |
|  |  | (0.169)** | (0.174)** | (0.170)** |
|  | **Period** | -2.132 | -2.785 | -2.360 |
|  |  | (0.065)** | (0.134)** | (0.099)** |
|  | **Individual** | -0.266 | -0.302 | -0.270 |
|  |  | (0.002)** | (0.005)** | (0.005)** |
| ***N*** |  | 217,542 | 21,500 | 20,647 |

## Table A13. Multilevel hierarchical regression coefficients for the dependent variable “allow immigrants from poor countries” for the three separate samples

|  |  | **Non-migrants** | **Migrants** | **Second generation** |
| --- | --- | --- | --- | --- |
| **Allow poor** | **Age** | -0.009 | -0.013 | -0.012 |
|  |  | (0.001)** | (0.002)** | (0.002)** |
|  | **Age squared** | 0.000 | 0.000 | 0.000 |
|  |  | (0.000)** | (0.000)** | (0.000)+ |
|  | **Male** | -0.043 | -0.007 | -0.004 |
|  |  | (0.004)** | (0.011) | (0.012) |
|  | **In paid work** | -0.021 | 0.042 | -0.032 |
|  |  | (0.004)** | (0.013)** | (0.014)* |
|  | **Secondary education** | 0.077 | -0.026 | 0.043 |
|  | ***(Ref Elementary)*** | (0.005)** | (0.015)+ | (0.016)** |
|  | **Vocational education** | 0.150 | -0.018 | 0.123 |
|  |  | (0.009)** | (0.027) | (0.028)** |
|  | **Tertiary education** | 0.338 | 0.164 | 0.304 |
|  |  | (0.005)** | (0.016)** | (0.018)** |
|  | **Coping on income** | -0.081 | -0.071 | -0.062 |
|  | ***(Ref Satisfied)*** | (0.004)** | (0.014)** | (0.014)** |
|  | **Difficult on income** | -0.175 | -0.076 | -0.139 |
|  |  | (0.006)** | (0.018)** | (0.019)** |
|  | **Very difficult on income** | -0.267 | -0.109 | -0.217 |
|  |  | (0.009)** | (0.025)** | (0.028)** |
|  | **Monthly socialising** | 0.112 | 0.144 | 0.235 |
|  | ***(Ref Never)*** | (0.016)** | (0.046)** | (0.051)** |
|  | **Weekly socialising** | 0.170 | 0.158 | 0.263 |
|  |  | (0.016)** | (0.045)** | (0.050)** |
|  | **Daily socialising** | 0.218 | 0.212 | 0.324 |
|  |  | (0.016)** | (0.045)** | (0.050)** |
|  | **Discriminated** | -0.055 | 0.097 | 0.096 |
|  | ***(Ref Non-discrim)*** | (0.016)** | (0.017)** | (0.024)** |
|  | **Minority** | 0.070 | 0.046 | 0.056 |
|  | ***(Ref Non-minor)*** | (0.015)** | (0.014)** | (0.020)** |
|  | **Citizenship** | -0.012 | -0.008 | 0.003 |
|  | ***(Ref Non citizen)*** | (0.005)* | (0.001)** | (0.002) |
|  | **Roman Catholic** | -0.085 | -0.103 | -0.139 |
|  | ***(Ref No-religion)*** | (0.006)** | (0.017)** | (0.018)** |
|  | **Protestant** | -0.068 | -0.054 | -0.081 |
|  |  | (0.006)** | (0.022)* | (0.022)** |
|  | **Eastern Orthodox** | -0.124 | -0.130 | -0.119 |
|  |  | (0.022)** | (0.023)** | (0.030)** |
|  | **Other Christian religions** | 0.121 | -0.002 | 0.072 |
|  |  | (0.018)** | (0.037) | (0.050) |
|  | **Other Non-Christian religions** | 0.163 | 0.007 | -0.039 |
|  |  | (0.032)** | (0.053) | (0.067) |
|  | **Islam** | 0.211 | 0.082 | 0.027 |
|  |  | (0.058)** | (0.021)** | (0.030) |
|  | **Eastern Religions** | 0.318 | -0.050 | 0.053 |
|  |  | (0.043)** | (0.041) | (0.065) |
|  | **Religiosity** | 0.011 | 0.008 | 0.011 |
|  | ***(Scale)*** | (0.001)** | (0.002)** | (0.002)** |
|  | **Language spoken at home** | -0.072 | -0.021 | -0.023 |
|  |  | (0.016)** | (0.016) | (0.023) |
|  | **ESS round 2 / 2004** | -0.085 | -0.028 | -0.070 |
|  | ***(Ref ESS 1/2002)*** | (0.048)+ | (0.043) | (0.045) |
|  | **ESS round 3** **/ 2006** | -0.105 | -0.099 | -0.067 |
|  |  | (0.048)* | (0.043)* | (0.046) |
|  | **ESS round 4** **/ 2008** | -0.067 | -0.057 | -0.044 |
|  |  | (0.045) | (0.041) | (0.044) |
|  | **ESS round 5** **/ 2010** | -0.084 | -0.080 | -0.052 |
|  |  | (0.045)+ | (0.040)* | (0.043) |
|  | **ESS round 6** **/ 2012** | -0.056 | -0.044 | -0.035 |
|  |  | (0.046) | (0.042) | (0.044) |
|  | **ESS round 7** **/ 2014** | -0.084 | -0.136 | -0.100 |
|  |  | (0.046)+ | (0.041)** | (0.043)* |
|  | **ESS round 8** **/ 2016** | 0.035 | -0.054 | 0.038 |
|  |  | (0.046) | (0.042) | (0.044) |
|  | **ESS round 9** **/ 2018** | 0.060 | -0.016 | 0.063 |
|  |  | (0.045) | (0.040) | (0.043) |
|  | **Intercept** | 2.729 | 2.986 | 2.685 |
|  |  | (0.082)** | (0.085)** | (0.093)** |
| **Random effects** | **Country** | -1.428 | -1.548 | -1.414 |
|  |  | (0.167)** | (0.168)** | (0.165)** |
|  | **Period** | -2.075 | -2.510 | -2.417 |
|  |  | (0.065)** | (0.116)** | (0.111)** |
|  | **Individual** | -0.223 | -0.207 | -0.194 |
|  |  | (0.002)** | (0.005)** | (0.005)** |
| ***N*** |  | 216,940 | 21,457 | 20,585 |

## Table A14. Multilevel hierarchical regression coefficients for the dependent variable “immigration effect on country in general” for the three separate samples

|  |  | **Non-migrants** | **Migrants** | **Second generation** |
| --- | --- | --- | --- | --- |
| **General effect** | **Age** | -0.004 | -0.001 | -0.005 |
|  |  | (0.000)** | (0.002) | (0.002)** |
|  | **Age squared** | 0.000 | -0.000 | 0.000 |
|  |  | (0.000)** | (0.000)+ | (0.000) |
|  | **Male** | -0.010 | 0.032 | 0.011 |
|  |  | (0.003)** | (0.009)** | (0.009) |
|  | **In paid work** | -0.006 | 0.043 | -0.037 |
|  |  | (0.003)+ | (0.011)** | (0.011)** |
|  | **Secondary education** | 0.079 | -0.002 | 0.071 |
|  | ***(Ref Elementary)*** | (0.004)** | (0.012) | (0.012)** |
|  | **Vocational education** | 0.150 | 0.058 | 0.129 |
|  |  | (0.007)** | (0.021)** | (0.022)** |
|  | **Tertiary education** | 0.293 | 0.141 | 0.297 |
|  |  | (0.004)** | (0.012)** | (0.014)** |
|  | **Coping on income** | -0.090 | -0.066 | -0.091 |
|  | ***(Ref Satisfied)*** | (0.003)** | (0.011)** | (0.011)** |
|  | **Difficult on income** | -0.191 | -0.087 | -0.168 |
|  |  | (0.005)** | (0.014)** | (0.015)** |
|  | **Very difficult on income** | -0.295 | -0.133 | -0.246 |
|  |  | (0.007)** | (0.019)** | (0.022)** |
|  | **Monthly socialising** | 0.105 | -0.007 | 0.178 |
|  | ***(Ref Never)*** | (0.012)** | (0.036) | (0.039)** |
|  | **Weekly socialising** | 0.162 | 0.036 | 0.225 |
|  |  | (0.012)** | (0.035) | (0.039)** |
|  | **Daily socialising** | 0.182 | 0.076 | 0.261 |
|  |  | (0.012)** | (0.035)* | (0.039)** |
|  | **Discriminated** | -0.174 | 0.024 | 0.032 |
|  | ***(Ref Non-discrim)*** | (0.012)** | (0.013)+ | (0.019)+ |
|  | **Minority** | 0.032 | 0.098 | 0.090 |
|  | ***(Ref Non-minor)*** | (0.012)** | (0.011)** | (0.016)** |
|  | **Citizenship** | -0.009 | -0.010 | -0.002 |
|  | ***(Ref Non citizen)*** | (0.004)* | (0.001)** | (0.002) |
|  | **Roman Catholic** | -0.068 | -0.083 | -0.107 |
|  | ***(Ref No-religion)*** | (0.004)** | (0.013)** | (0.014)** |
|  | **Protestant** | -0.049 | -0.068 | -0.040 |
|  |  | (0.004)** | (0.018)** | (0.017)* |
|  | **Eastern Orthodox** | -0.032 | -0.085 | -0.059 |
|  |  | (0.017)+ | (0.018)** | (0.023)* |
|  | **Other Christian religions** | 0.020 | -0.084 | 0.056 |
|  |  | (0.014) | (0.029)** | (0.039) |
|  | **Other Non-Christian religions** | 0.110 | -0.074 | -0.004 |
|  |  | (0.025)** | (0.043)+ | (0.051) |
|  | **Islam** | 0.366 | 0.092 | 0.168 |
|  |  | (0.044)** | (0.017)** | (0.023)** |
|  | **Eastern Religions** | 0.203 | -0.010 | 0.042 |
|  |  | (0.033)** | (0.032) | (0.049) |
|  | **Religiosity** | 0.011 | 0.015 | 0.011 |
|  | ***(Scale)*** | (0.001)** | (0.002)** | (0.002)** |
|  | **Language spoken at home** | -0.002 | 0.027 | -0.002 |
|  |  | (0.012) | (0.012)* | (0.018) |
|  | **ESS round 2 / 2004** | -0.013 | -0.032 | 0.004 |
|  | ***(Ref ESS 1/2002)*** | (0.028) | (0.038) | (0.034) |
|  | **ESS round 3** **/ 2006** | 0.002 | 0.005 | 0.055 |
|  |  | (0.028) | (0.038) | (0.034) |
|  | **ESS round 4** **/ 2008** | 0.031 | 0.030 | 0.067 |
|  |  | (0.027) | (0.036) | (0.033)* |
|  | **ESS round 5** **/ 2010** | 0.021 | -0.001 | 0.069 |
|  |  | (0.027) | (0.036) | (0.032)* |
|  | **ESS round 6** **/ 2012** | 0.064 | 0.049 | 0.106 |
|  |  | (0.027)* | (0.036) | (0.033)** |
|  | **ESS round 7** **/ 2014** | 0.052 | -0.001 | 0.098 |
|  |  | (0.027)+ | (0.036) | (0.032)** |
|  | **ESS round 8** **/ 2016** | 0.064 | -0.008 | 0.086 |
|  |  | (0.027)* | (0.037) | (0.033)** |
|  | **ESS round 9** **/ 2018** | 0.114 | 0.033 | 0.120 |
|  |  | (0.026)** | (0.035) | (0.032)** |
|  | **Intercept** | 2.438 | 2.728 | 2.411 |
|  |  | (0.057)** | (0.066)** | (0.068)** |
| **Random effects** | **Country** | -1.836 | -1.911 | -1.853 |
|  |  | (0.164)** | (0.169)** | (0.167)** |
|  | **Period** | -2.617 | -2.565 | -2.729 |
|  |  | (0.066)** | (0.101)** | (0.112)** |
|  | **Individual** | -0.486 | -0.447 | -0.456 |
|  |  | (0.002)** | (0.005)** | (0.005)** |
| ***N*** |  | 216,015 | 21,322 | 20,452 |

## Table A15. Multilevel hierarchical regression coefficients for the dependent variable “immigration effect on economy” for the three separate samples

|  |  | **Non-migrants** | **Migrants** | **Second generation** |
| --- | --- | --- | --- | --- |
| **Effect on economy** | **Age** | -0.003 | -0.004 | -0.009 |
|  |  | (0.000)** | (0.002)* | (0.002)** |
|  | **Age squared** | 0.000 | 0.000 | 0.000 |
|  |  | (0.000)** | (0.000) | (0.000)** |
|  | **Male** | 0.062 | 0.091 | 0.084 |
|  |  | (0.003)** | (0.010)** | (0.010)** |
|  | **In paid work** | -0.015 | 0.049 | -0.035 |
|  |  | (0.004)** | (0.011)** | (0.012)** |
|  | **Secondary education** | 0.096 | 0.033 | 0.093 |
|  | ***(Ref Elementary)*** | (0.004)** | (0.013)** | (0.013)** |
|  | **Vocational education** | 0.170 | 0.058 | 0.155 |
|  |  | (0.008)** | (0.023)* | (0.023)** |
|  | **Tertiary education** | 0.362 | 0.216 | 0.368 |
|  |  | (0.004)** | (0.013)** | (0.015)** |
|  | **Coping on income** | -0.108 | -0.070 | -0.096 |
|  | ***(Ref Satisfied)*** | (0.003)** | (0.012)** | (0.012)** |
|  | **Difficult on income** | -0.214 | -0.112 | -0.187 |
|  |  | (0.005)** | (0.015)** | (0.016)** |
|  | **Very difficult on income** | -0.318 | -0.139 | -0.281 |
|  |  | (0.008)** | (0.021)** | (0.023)** |
|  | **Monthly socialising** | 0.130 | 0.038 | 0.124 |
|  | ***(Ref Never)*** | (0.013)** | (0.039) | (0.042)** |
|  | **Weekly socialising** | 0.201 | 0.096 | 0.191 |
|  |  | (0.013)** | (0.038)* | (0.042)** |
|  | **Daily socialising** | 0.215 | 0.126 | 0.214 |
|  |  | (0.013)** | (0.038)** | (0.042)** |
|  | **Discriminated** | -0.141 | 0.066 | 0.046 |
|  | ***(Ref Non-discrim)*** | (0.013)** | (0.014)** | (0.020)* |
|  | **Minority** | 0.020 | 0.098 | 0.077 |
|  | ***(Ref Non-minor)*** | (0.012) | (0.012)** | (0.017)** |
|  | **Citizenship** | -0.014 | -0.012 | 0.003 |
|  | ***(Ref Non citizen)*** | (0.004)** | (0.001)** | (0.002)+ |
|  | **Roman Catholic** | -0.051 | -0.036 | -0.079 |
|  | ***(Ref No-religion)*** | (0.005)** | (0.014)* | (0.015)** |
|  | **Protestant** | -0.029 | -0.036 | -0.014 |
|  |  | (0.005)** | (0.019)+ | (0.018) |
|  | **Eastern Orthodox** | -0.042 | -0.071 | -0.095 |
|  |  | (0.018)* | (0.019)** | (0.025)** |
|  | **Other Christian religions** | 0.028 | -0.040 | 0.059 |
|  |  | (0.015)+ | (0.031) | (0.041) |
|  | **Other Non-Christian religions** | 0.110 | -0.010 | 0.012 |
|  |  | (0.026)** | (0.045) | (0.055) |
|  | **Islam** | 0.288 | 0.053 | 0.118 |
|  |  | (0.047)** | (0.018)** | (0.025)** |
|  | **Eastern Religions** | 0.176 | -0.011 | 0.059 |
|  |  | (0.035)** | (0.034) | (0.053) |
|  | **Religiosity** | 0.010 | 0.007 | 0.008 |
|  | ***(Scale)*** | (0.001)** | (0.002)** | (0.002)** |
|  | **Language spoken at home** | -0.002 | 0.009 | 0.011 |
|  |  | (0.013) | (0.013) | (0.020) |
|  | **ESS round 2 / 2004** | -0.055 | -0.054 | -0.011 |
|  | ***(Ref ESS 1/2002)*** | (0.034) | (0.037) | (0.038) |
|  | **ESS round 3** **/ 2006** | 0.016 | 0.010 | 0.059 |
|  |  | (0.034) | (0.037) | (0.039) |
|  | **ESS round 4** **/ 2008** | -0.001 | -0.024 | 0.048 |
|  |  | (0.032) | (0.035) | (0.037) |
|  | **ESS round 5** **/ 2010** | -0.032 | -0.045 | -0.001 |
|  |  | (0.032) | (0.034) | (0.037) |
|  | **ESS round 6** **/ 2012** | 0.007 | 0.012 | 0.054 |
|  |  | (0.033) | (0.035) | (0.037) |
|  | **ESS round 7** **/ 2014** | -0.020 | -0.072 | 0.007 |
|  |  | (0.032) | (0.035)* | (0.037) |
|  | **ESS round 8** **/ 2016** | 0.029 | -0.036 | 0.049 |
|  |  | (0.033) | (0.036) | (0.037) |
|  | **ESS round 9** **/ 2018** | 0.121 | 0.048 | 0.119 |
|  |  | (0.032)** | (0.034) | (0.036)** |
|  | **Intercept** | 2.395 | 2.713 | 2.438 |
|  |  | (0.057)** | (0.068)** | (0.070)** |
| **Random effects** | **Country** | -2.034 | -1.964 | -2.017 |
|  |  | (0.171)** | (0.170)** | (0.173)** |
|  | **Period** | -2.426 | -2.666 | -2.570 |
|  |  | (0.065)** | (0.114)** | (0.103)** |
|  | **Individual** | -0.421 | -0.377 | -0.383 |
|  |  | (0.002)** | (0.005)** | (0.005)** |
| ***N*** |  | 215,234 | 21,439 | 20,489 |

## Table A16. Multilevel hierarchical regression coefficients for the dependent variable “immigration effect on culture” for the three separate samples

|  |  | **Non-migrants** | **Migrants** | **Second generation** |
| --- | --- | --- | --- | --- |
| **Effect on culture** | **Age** | -0.001 | 0.002 | -0.002 |
|  |  | (0.000) | (0.002) | (0.002) |
|  | **Age squared** | -0.000 | -0.000 | -0.000 |
|  |  | (0.000)** | (0.000)** | (0.000) |
|  | **Male** | -0.046 | 0.009 | -0.029 |
|  |  | (0.003)** | (0.010) | (0.010)** |
|  | **In paid work** | -0.009 | 0.049 | -0.034 |
|  |  | (0.004)* | (0.011)** | (0.012)** |
|  | **Secondary education** | 0.108 | 0.013 | 0.105 |
|  | ***(Ref Elementary)*** | (0.004)** | (0.012) | (0.014)** |
|  | **Vocational education** | 0.192 | 0.062 | 0.155 |
|  |  | (0.008)** | (0.022)** | (0.024)** |
|  | **Tertiary education** | 0.378 | 0.197 | 0.376 |
|  |  | (0.004)** | (0.013)** | (0.015)** |
|  | **Coping on income** | -0.093 | -0.053 | -0.063 |
|  | ***(Ref Satisfied)*** | (0.003)** | (0.012)** | (0.012)** |
|  | **Difficult on income** | -0.174 | -0.082 | -0.127 |
|  |  | (0.005)** | (0.015)** | (0.016)** |
|  | **Very difficult on income** | -0.253 | -0.109 | -0.176 |
|  |  | (0.008)** | (0.020)** | (0.024)** |
|  | **Monthly socialising** | 0.126 | 0.001 | 0.177 |
|  | ***(Ref Never)*** | (0.013)** | (0.038) | (0.043)** |
|  | **Weekly socialising** | 0.187 | 0.037 | 0.238 |
|  |  | (0.013)** | (0.037) | (0.042)** |
|  | **Daily socialising** | 0.219 | 0.093 | 0.285 |
|  |  | (0.013)** | (0.038)* | (0.042)** |
|  | **Discriminated** | -0.192 | 0.072 | 0.059 |
|  | ***(Ref Non-discrim)*** | (0.013)** | (0.014)** | (0.020)** |
|  | **Minority** | 0.037 | 0.103 | 0.106 |
|  | ***(Ref Non-minor)*** | (0.013)** | (0.011)** | (0.017)** |
|  | **Citizenship** | -0.010 | -0.009 | 0.000 |
|  | ***(Ref Non citizen)*** | (0.004)* | (0.001)** | (0.002) |
|  | **Roman Catholic** | -0.091 | -0.104 | -0.155 |
|  | ***(Ref No-religion)*** | (0.005)** | (0.014)** | (0.015)** |
|  | **Protestant** | -0.065 | -0.088 | -0.061 |
|  |  | (0.005)** | (0.018)** | (0.019)** |
|  | **Eastern Orthodox** | -0.047 | -0.114 | -0.058 |
|  |  | (0.018)** | (0.019)** | (0.025)* |
|  | **Other Christian religions** | 0.024 | -0.072 | 0.051 |
|  |  | (0.015) | (0.031)* | (0.042) |
|  | **Other Non-Christian religions** | 0.138 | -0.121 | -0.099 |
|  |  | (0.027)** | (0.045)** | (0.057)+ |
|  | **Islam** | 0.408 | 0.088 | 0.156 |
|  |  | (0.048)** | (0.018)** | (0.025)** |
|  | **Eastern Religions** | 0.263 | -0.024 | 0.116 |
|  |  | (0.036)** | (0.034) | (0.054)* |
|  | **Religiosity** | 0.008 | 0.013 | 0.009 |
|  | ***(Scale)*** | (0.001)** | (0.002)** | (0.002)** |
|  | **Language spoken at home** | -0.104 | -0.013 | -0.097 |
|  |  | (0.013)** | (0.013) | (0.020)** |
|  | **ESS round 2 / 2004** | -0.067 | -0.051 | -0.011 |
|  | ***(Ref ESS 1/2002)*** | (0.029)* | (0.033) | (0.035) |
|  | **ESS round 3** **/ 2006** | -0.072 | -0.071 | -0.059 |
|  |  | (0.029)* | (0.033)* | (0.035)+ |
|  | **ESS round 4** **/ 2008** | -0.044 | -0.048 | -0.031 |
|  |  | (0.027) | (0.032) | (0.033) |
|  | **ESS round 5** **/ 2010** | -0.079 | -0.087 | -0.070 |
|  |  | (0.027)** | (0.031)** | (0.033)* |
|  | **ESS round 6** **/ 2012** | -0.038 | -0.024 | -0.017 |
|  |  | (0.028) | (0.032) | (0.034) |
|  | **ESS round 7** **/ 2014** | -0.071 | -0.127 | -0.023 |
|  |  | (0.027)* | (0.032)** | (0.033) |
|  | **ESS round 8** **/ 2016** | -0.083 | -0.150 | -0.076 |
|  |  | (0.028)** | (0.033)** | (0.034)* |
|  | **ESS round 9** **/ 2018** | -0.046 | -0.117 | -0.031 |
|  |  | (0.027)+ | (0.031)** | (0.033) |
|  | **Intercept** | 2.627 | 2.851 | 2.594 |
|  |  | (0.069)** | (0.067)** | (0.079)** |
| **Random effects** | **Country** | -1.521 | -1.881 | -1.562 |
|  |  | (0.162)** | (0.169)** | (0.165)** |
|  | **Period** | -2.599 | -2.828 | -2.760 |
|  |  | (0.067)** | (0.132)** | (0.120)** |
|  | **Individual** | -0.400 | -0.389 | -0.361 |
|  |  | (0.002)** | (0.005)** | (0.005)** |
| ***N*** |  | 215,970 | 21,602 | 20,644 |


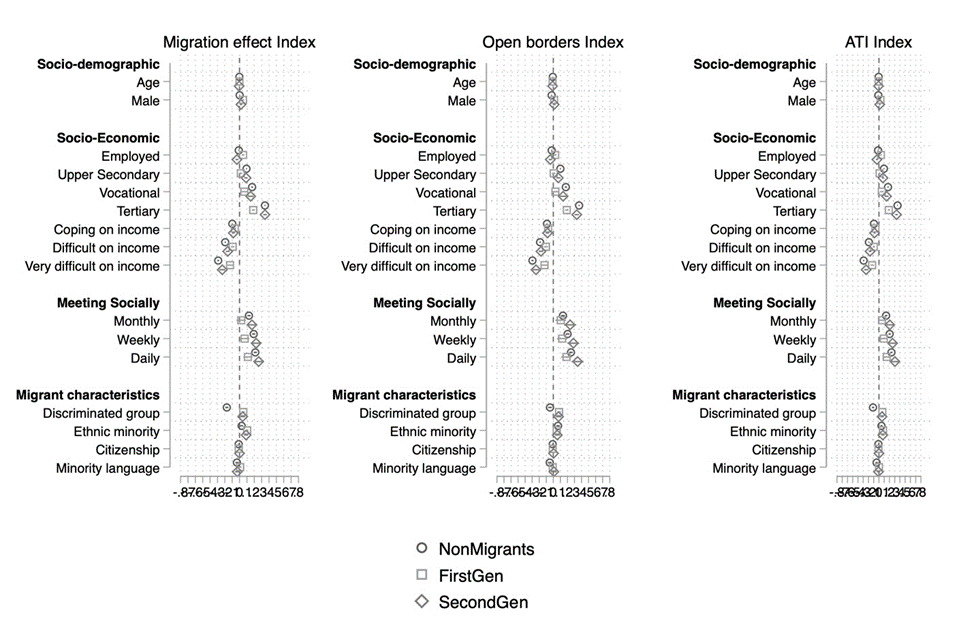


## Figure A1. Determinants of attitudes to immigration within the three samples with different operationalization of the dependent variables

## Table A17. Correlation analysis of six measures of attitudes towards immigrants

|  | **Cultural contribution** | **Economic contribution** | **General contribution** | **Same race** | **Different race** | **Poor country** |
| --- | --- | --- | --- | --- | --- | --- |
| **Cultural contribution** | 1.0000 |  |  |  |  |  |
| **Economic contribution** | 0.6224* | 1.0000 |  |  |  |  |
| **General contribution** | 0.6965* | 0.6500* | 1.0000 |  |  |  |
| **Same race** | 0.4382* | 0.4606* | 0.4557* | 1.0000 |  |  |
| **Different race** | 0.5244* | 0.5145* | 0.5439* | 0.7365* | 1.0000 |  |
| **Poor country** | 0.4961* | 0.4881* | 0.5160* | 0.6413* | 0.7891* | 1.0000 |

## Table A18. Factor analysis (including factor loadings and rotated factor loadings) of six measures of attitudes towards immigrants

Factor analysis/correlation Number of obs = 243,304

Method: iterated principal factors Retained factors = 5

Rotation: (unrotated) Number of params = 15

| **Factor** | **Eigenvalue** | **Difference** | **Proportion** | **Cumulative** |
| --- | --- | --- | --- | --- |
| Factor1 | 3.59585 | 2.98532 | 0.8405 | 0.8405 |
| Factor2 | 0.61052 | 0.55740 | 0.1427 | 0.9832 |
| Factor3 | 0.05313 | 0.03621 | 0.0124 | 0.9956 |
| Factor4 | 0.01691 | 0.01496 | 0.0040 | 0.9996 |
| Factor5 | 0.00195 | 0.00214 | 0.0005 | 1.0000 |
| Factor6 | -0.00019 | . | -0.0000 | 1.0000 |

LR test: independent vs. saturated: chi2(15) = 8.8e+05 Prob>chi2 = 0.0000

Factor loadings (pattern matrix) and unique variances

| **Variable** | **Factor1** | **Factor2** | **Factor3** | **Factor4** | **Factor5** | **Uniqueness** |
| --- | --- | --- | --- | --- | --- | --- |
| **Cultural contribution** | 0.7439 | 0.3420 | -0.0449 | -0.0416 | 0.0316 | 0.3249 |
| **Economic contribution** | 0.7203 | 0.2836 | 0.1060 | 0.0675 | -0.0047 | 0.3849 |
| **General contribution** | 0.7714 | 0.3604 | -0.0427 | -0.0244 | -0.0270 | 0.2718 |
| **Same race** | 0.7348 | -0.2858 | 0.1565 | -0.0242 | 0.0063 | 0.3532 |
| **Different race** | 0.8705 | -0.3590 | -0.0462 | -0.0528 | -0.0108 | 0.1083 |
| **Poor country** | 0.7942 | -0.2696 | -0.1068 | 0.0816 | 0.0068 | 0.2785 |

Factor analysis/correlation Number of obs = 243,304

Method: iterated principal factors Retained factors = 5

Rotation: oblique promax (Kaiser on) Number of params = 15

Rotated factor loadings (pattern matrix) and unique variances

| **Variable** | **Factor1** | **Factor2** | **Factor3** | **Factor4** | **Factor5** | **Uniqueness** |
| --- | --- | --- | --- | --- | --- | --- |
| **Cultural contribution** |  | 0.8019 |  |  |  | 0.3249 |
| **Economic contribution** |  | 0.7081 |  |  |  | 0.3849 |
| **General contribution** |  | 0.8227 |  |  |  | 0.2718 |
| **Same race** | 0.7840 |  |  |  |  | 0.3532 |
| **Different race** | 0.9135 |  |  |  |  | 0.1083 |
| **Poor country** | 0.7643 |  |  |  |  | 0.2785 |

(blanks represent abs(loading)<.3). **Cronbach Alpha results:** α = 0.8892

## Table A19. Multilevel hierarchical regression coefficients for the index “attitude toward the effects of immigration” for the three separate samples

|  |  | **Non-migrants** | **Migrants** | **Second generation** |
| --- | --- | --- | --- | --- |
| **Index: attitude toward the effects of immigration** | **Age** | -0.002 | -0.001 | -0.005 |
|  |  | (0.000)** | (0.001) | (0.001)** |
|  | **Age squared** | 0.000 | -0.000 | 0.000 |
|  |  | (0.000) | (0.000)+ | (0.000) |
|  | **Male** | 0.002 | 0.047 | 0.020 |
|  |  | (0.003) | (0.008)** | (0.008)* |
|  | **In paid work** | -0.010 | 0.048 | -0.035 |
|  |  | (0.003)** | (0.009)** | (0.010)** |
|  | **Secondary education** | 0.094 | 0.017 | 0.089 |
|  | ***(Ref Elementary)*** | (0.003)** | (0.011) | (0.011)** |
|  | **Vocational education** | 0.171 | 0.065 | 0.151 |
|  |  | (0.007)** | (0.019)** | (0.020)** |
|  | **Tertiary education** | 0.345 | 0.186 | 0.346 |
|  |  | (0.004)** | (0.011)** | (0.013)** |
|  | **Coping on income** | -0.098 | -0.064 | -0.084 |
|  | ***(Ref Satisfied)*** | (0.003)** | (0.010)** | (0.010)** |
|  | **Difficult on income** | -0.194 | -0.093 | -0.160 |
|  |  | (0.004)** | (0.012)** | (0.013)** |
|  | **Very difficult on income** | -0.290 | -0.128 | -0.234 |
|  |  | (0.007)** | (0.017)** | (0.020)** |
|  | **Monthly socialising** | 0.128 | 0.022 | 0.168 |
|  | ***(Ref Never)*** | (0.011)** | (0.032) | (0.036)** |
|  | **Weekly socialising** | 0.192 | 0.070 | 0.225 |
|  |  | (0.011)** | (0.032)* | (0.036)** |
|  | **Daily socialising** | 0.214 | 0.114 | 0.260 |
|  |  | (0.011)** | (0.032)** | (0.036)** |
|  | **Discriminated** | -0.172 | 0.051 | 0.043 |
|  | ***(Ref Non-discrim)*** | (0.011)** | (0.012)** | (0.017)* |
|  | **Minority** | 0.029 | 0.103 | 0.089 |
|  | ***(Ref Non-minor)*** | (0.011)** | (0.010)** | (0.014)** |
|  | **Citizenship** | -0.011 | -0.011 | 0.000 |
|  | ***(Ref Non citizen)*** | (0.003)** | (0.001)** | (0.002) |
|  | **Roman Catholic** | -0.070 | -0.073 | -0.114 |
|  | ***(Ref No-religion)*** | (0.004)** | (0.012)** | (0.012)** |
|  | **Protestant** | -0.048 | -0.065 | -0.036 |
|  |  | (0.004)** | (0.016)** | (0.016)* |
|  | **Eastern Orthodox** | -0.041 | -0.089 | -0.067 |
|  |  | (0.016)** | (0.016)** | (0.021)** |
|  | **Other Christian religions** | 0.024 | -0.068 | 0.044 |
|  |  | (0.013)+ | (0.026)** | (0.036) |
|  | **Other Non-Christian religions** | 0.119 | -0.071 | -0.023 |
|  |  | (0.023)** | (0.038)+ | (0.048) |
|  | **Islam** | 0.360 | 0.081 | 0.156 |
|  |  | (0.040)** | (0.015)** | (0.021)** |
|  | **Eastern Religions** | 0.210 | -0.013 | 0.083 |
|  |  | (0.030)** | (0.028) | (0.045)+ |
|  | **Religiosity** | 0.009 | 0.012 | 0.009 |
|  | ***(Scale)*** | (0.001)** | (0.002)** | (0.002)** |
|  | **Language spoken at home** | -0.035 | 0.008 | -0.030 |
|  |  | (0.011)** | (0.011) | (0.017)+ |
|  | **ESS round 2 / 2004** | -0.045 | -0.050 | -0.011 |
|  | ***(Ref ESS 1/2002)*** | (0.028) | (0.033) | (0.032) |
|  | **ESS round 3** **/ 2006** | -0.016 | -0.016 | 0.018 |
|  |  | (0.029) | (0.033) | (0.033) |
|  | **ESS round 4** **/ 2008** | -0.004 | -0.010 | 0.034 |
|  |  | (0.027) | (0.032) | (0.031) |
|  | **ESS round 5** **/ 2010** | -0.030 | -0.044 | 0.005 |
|  |  | (0.027) | (0.031) | (0.031) |
|  | **ESS round 6** **/ 2012** | 0.011 | 0.015 | 0.051 |
|  |  | (0.028) | (0.032) | (0.031) |
|  | **ESS round 7** **/ 2014** | -0.012 | -0.066 | 0.031 |
|  |  | (0.027) | (0.032)* | (0.031) |
|  | **ESS round 8** **/ 2016** | 0.005 | -0.069 | 0.025 |
|  |  | (0.028) | (0.033)* | (0.031) |
|  | **ESS round 9** **/ 2018** | 0.064 | -0.015 | 0.072 |
|  |  | (0.027)* | (0.031) | (0.030)* |
|  | **Intercept** | 2.476 | 2.739 | 2.478 |
|  |  | (0.055)** | (0.059)** | (0.064)** |
| **Random effects** | **Country** | -1.850 | -1.999 | -1.869 |
|  |  | (0.165)** | (0.170)** | (0.167)** |
|  | **Period** | -2.600 | -2.704 | -2.762 |
|  |  | (0.066)** | (0.105)** | (0.105)** |
|  | **Individual** | -0.583 | -0.580 | -0.557 |
|  |  | (0.002)** | (0.005)** | (0.005)** |
| ***N*** |  | 208,913 | 20,589 | 19,892 |

## Table A20. Multilevel hierarchical regression coefficients for the index “attitude toward open borders” for the three separate samples

|  |  | **Non-migrants** | **Migrants** | **Second generation** |
| --- | --- | --- | --- | --- |
| **Index: attitude toward open borders** | **Age** | -0.007 | -0.010 | -0.010 |
|  |  | (0.000)** | (0.002)** | (0.002)** |
|  | **Age squared** | 0.000 | 0.000 | 0.000 |
|  |  | (0.000)** | (0.000)* | (0.000)* |
|  | **Male** | -0.024 | 0.013 | 0.013 |
|  |  | (0.003)** | (0.010) | (0.010) |
|  | **In paid work** | -0.023 | 0.026 | -0.042 |
|  |  | (0.004)** | (0.011)* | (0.012)** |
|  | **Secondary education** | 0.100 | 0.006 | 0.072 |
|  | ***(Ref Elementary)*** | (0.004)** | (0.013) | (0.014)** |
|  | **Vocational education** | 0.178 | 0.030 | 0.139 |
|  |  | (0.008)** | (0.023) | (0.024)** |
|  | **Tertiary education** | 0.366 | 0.191 | 0.333 |
|  |  | (0.005)** | (0.013)** | (0.015)** |
|  | **Coping on income** | -0.090 | -0.074 | -0.083 |
|  | ***(Ref Satisfied)*** | (0.004)** | (0.012)** | (0.012)** |
|  | **Difficult on income** | -0.188 | -0.105 | -0.175 |
|  |  | (0.005)** | (0.015)** | (0.016)** |
|  | **Very difficult on income** | -0.296 | -0.125 | -0.246 |
|  |  | (0.008)** | (0.021)** | (0.024)** |
|  | **Monthly socialising** | 0.137 | 0.106 | 0.239 |
|  | ***(Ref Never)*** | (0.014)** | (0.039)** | (0.044)** |
|  | **Weekly socialising** | 0.203 | 0.127 | 0.285 |
|  |  | (0.014)** | (0.038)** | (0.043)** |
|  | **Daily socialising** | 0.250 | 0.187 | 0.345 |
|  |  | (0.014)** | (0.038)** | (0.043)** |
|  | **Discriminated** | -0.047 | 0.081 | 0.075 |
|  | ***(Ref Non-discrim)*** | (0.014)** | (0.014)** | (0.020)** |
|  | **Minority** | 0.067 | 0.048 | 0.057 |
|  | ***(Ref Non-minor)*** | (0.013)** | (0.012)** | (0.017)** |
|  | **Citizenship** | -0.009 | -0.008 | 0.003 |
|  | ***(Ref Non citizen)*** | (0.004)* | (0.001)** | (0.002)+ |
|  | **Roman Catholic** | -0.078 | -0.069 | -0.133 |
|  | ***(Ref No-religion)*** | (0.005)** | (0.014)** | (0.015)** |
|  | **Protestant** | -0.051 | -0.020 | -0.056 |
|  |  | (0.005)** | (0.019) | (0.019)** |
|  | **Eastern Orthodox** | -0.077 | -0.099 | -0.096 |
|  |  | (0.019)** | (0.019)** | (0.025)** |
|  | **Other Christian religions** | 0.116 | -0.006 | 0.103 |
|  |  | (0.015)** | (0.031) | (0.043)* |
|  | **Other Non-Christian religions** | 0.130 | -0.011 | -0.041 |
|  |  | (0.028)** | (0.045) | (0.057) |
|  | **Islam** | 0.187 | 0.045 | -0.012 |
|  |  | (0.050)** | (0.018)* | (0.026) |
|  | **Eastern Religions** | 0.292 | -0.046 | -0.025 |
|  |  | (0.037)** | (0.034) | (0.055) |
|  | **Religiosity** | 0.008 | 0.003 | 0.010 |
|  | ***(Scale)*** | (0.001)** | (0.002)+ | (0.002)** |
|  | **Language spoken at home** | -0.051 | -0.008 | 0.008 |
|  |  | (0.014)** | (0.013) | (0.020) |
|  | **ESS round 2 / 2004** | -0.034 | 0.022 | -0.008 |
|  | ***(Ref ESS 1/2002)*** | (0.045) | (0.036) | (0.044) |
|  | **ESS round 3** **/ 2006** | -0.048 | -0.031 | -0.020 |
|  |  | (0.045) | (0.036) | (0.045) |
|  | **ESS round 4** **/ 2008** | -0.004 | 0.014 | 0.029 |
|  |  | (0.043) | (0.034) | (0.043) |
|  | **ESS round 5** **/ 2010** | -0.003 | -0.003 | 0.054 |
|  |  | (0.043) | (0.034) | (0.042) |
|  | **ESS round 6** **/ 2012** | 0.016 | 0.010 | 0.050 |
|  |  | (0.044) | (0.035) | (0.043) |
|  | **ESS round 7** **/ 2014** | 0.037 | -0.002 | 0.044 |
|  |  | (0.043) | (0.034) | (0.043) |
|  | **ESS round 8** **/ 2016** | 0.079 | 0.004 | 0.084 |
|  |  | (0.044)+ | (0.036) | (0.043)+ |
|  | **ESS round 9** **/ 2018** | 0.125 | 0.055 | 0.130 |
|  |  | (0.042)** | (0.034) | (0.042)** |
|  | **Intercept** | 2.683 | 3.013 | 2.668 |
|  |  | (0.071)** | (0.070)** | (0.079)** |
| **Random effects** | **Country** | -1.628 | -1.786 | -1.651 |
|  |  | (0.168)** | (0.168)** | (0.168)** |
|  | **Period** | -2.132 | -2.682 | -2.352 |
|  |  | (0.064)** | (0.116)** | (0.096)** |
|  | **Individual** | -0.364 | -0.393 | -0.358 |
|  |  | (0.002)** | (0.005)** | (0.005)** |
| ***N*** |  | 214,729 | 21,074 | 20,356 |

## Table A21. Multilevel hierarchical regression coefficients for the index of all six measures of attitudes for the three separate samples

|  |  | **Non-migrants** | **Migrants** | **Second generation** |
| --- | --- | --- | --- | --- |
| **Index of 6 immigration attitudes measures** | **Age** | -0.005 | -0.005 | -0.008 |
|  |  | (0.000)** | (0.001)** | (0.001)** |
|  | **Age squared** | 0.000 | 0.000 | 0.000 |
|  |  | (0.000)** | (0.000) | (0.000)* |
|  | **Male** | -0.011 | 0.029 | 0.015 |
|  |  | (0.003)** | (0.008)** | (0.008)+ |
|  | **In paid work** | -0.016 | 0.039 | -0.038 |
|  |  | (0.003)** | (0.009)** | (0.010)** |
|  | **Secondary education** | 0.097 | 0.012 | 0.077 |
|  | ***(Ref Elementary)*** | (0.003)** | (0.010) | (0.011)** |
|  | **Vocational education** | 0.173 | 0.055 | 0.145 |
|  |  | (0.007)** | (0.018)** | (0.020)** |
|  | **Tertiary education** | 0.355 | 0.188 | 0.337 |
|  |  | (0.004)** | (0.011)** | (0.013)** |
|  | **Coping on income** | -0.094 | -0.067 | -0.084 |
|  | ***(Ref Satisfied)*** | (0.003)** | (0.010)** | (0.010)** |
|  | **Difficult on income** | -0.191 | -0.097 | -0.170 |
|  |  | (0.004)** | (0.012)** | (0.013)** |
|  | **Very difficult on income** | -0.291 | -0.129 | -0.244 |
|  |  | (0.007)** | (0.017)** | (0.020)** |
|  | **Monthly socialising** | 0.137 | 0.060 | 0.207 |
|  | ***(Ref Never)*** | (0.012)** | (0.032)+ | (0.036)** |
|  | **Weekly socialising** | 0.205 | 0.094 | 0.260 |
|  |  | (0.011)** | (0.031)** | (0.035)** |
|  | **Daily socialising** | 0.239 | 0.149 | 0.306 |
|  |  | (0.011)** | (0.031)** | (0.036)** |
|  | **Discriminated** | -0.113 | 0.066 | 0.059 |
|  | ***(Ref Non-discrim)*** | (0.011)** | (0.011)** | (0.017)** |
|  | **Minority** | 0.049 | 0.078 | 0.074 |
|  | ***(Ref Non-minor)*** | (0.011)** | (0.009)** | (0.014)** |
|  | **Citizenship** | -0.010 | -0.009 | 0.001 |
|  | ***(Ref Non citizen)*** | (0.003)** | (0.001)** | (0.002) |
|  | **Roman Catholic** | -0.073 | -0.073 | -0.123 |
|  | ***(Ref No-religion)*** | (0.004)** | (0.011)** | (0.012)** |
|  | **Protestant** | -0.050 | -0.041 | -0.043 |
|  |  | (0.004)** | (0.015)** | (0.015)** |
|  | **Eastern Orthodox** | -0.057 | -0.092 | -0.085 |
|  |  | (0.016)** | (0.016)** | (0.021)** |
|  | **Other Christian religions** | 0.069 | -0.045 | 0.074 |
|  |  | (0.013)** | (0.026)+ | (0.036)* |
|  | **Other Non-Christian religions** | 0.128 | -0.026 | -0.025 |
|  |  | (0.023)** | (0.037) | (0.048) |
|  | **Islam** | 0.279 | 0.061 | 0.075 |
|  |  | (0.041)** | (0.014)** | (0.021)** |
|  | **Eastern Religions** | 0.251 | -0.035 | 0.028 |
|  |  | (0.030)** | (0.028) | (0.044) |
|  | **Religiosity** | 0.008 | 0.007 | 0.009 |
|  | ***(Scale)*** | (0.001)** | (0.001)** | (0.002)** |
|  | **Language spoken at home** | -0.044 | 0.001 | -0.009 |
|  |  | (0.011)** | (0.011) | (0.017) |
|  | **ESS round 2 / 2004** | -0.040 | -0.003 | -0.008 |
|  | ***(Ref ESS 1/2002)*** | (0.034) | (0.030) | (0.034) |
|  | **ESS round 3** **/ 2006** | -0.030 | -0.014 | 0.002 |
|  |  | (0.035) | (0.030) | (0.034) |
|  | **ESS round 4** **/ 2008** | -0.003 | 0.017 | 0.036 |
|  |  | (0.033) | (0.029) | (0.033) |
|  | **ESS round 5** **/ 2010** | -0.018 | -0.012 | 0.034 |
|  |  | (0.033) | (0.028) | (0.032) |
|  | **ESS round 6** **/ 2012** | 0.012 | 0.025 | 0.054 |
|  |  | (0.033) | (0.029) | (0.033) |
|  | **ESS round 7** **/ 2014** | 0.013 | -0.023 | 0.043 |
|  |  | (0.033) | (0.029) | (0.033) |
|  | **ESS round 8** **/ 2016** | 0.042 | -0.024 | 0.060 |
|  |  | (0.033) | (0.030) | (0.033)+ |
|  | **ESS round 9** **/ 2018** | 0.095 | 0.030 | 0.106 |
|  |  | (0.032)** | (0.028) | (0.032)** |
|  | **Intercept** | 2.574 | 2.858 | 2.574 |
|  |  | (0.058)** | (0.059)** | (0.065)** |
| **Random effects** | **Country** | -1.798 | -1.929 | -1.805 |
|  |  | (0.167)** | (0.167)** | (0.166)** |
|  | **Period** | -2.401 | -2.851 | -2.667 |
|  |  | (0.065)** | (0.114)** | (0.101)** |
|  | **Individual** | -0.589 | -0.631 | -0.580 |
|  |  | (0.002)** | (0.005)** | (0.005)** |
| ***N*** |  | 204,083 | 19,807 | 19,414 |
